# Hierarchical optimization predicts plasticity in the macaque inferior temporal cortex following object training

**DOI:** 10.1038/s41467-026-74816-0

**Published:** 2026-07-08

**Authors:** Lynn K. A. Sörensen, James J. DiCarlo, Kohitij Kar

**Affiliations:** 1 McGovern Institute for Brain Research, Dept. of Brain and Cognitive Sciences, Massachusetts Institute of Technology, Cambridge, USA; 2 Center for Brains, Minds and Machines, Massachusetts Institute of Technology, Cambridge, USA; 3 MIT Quest for Intelligence, Cambridge, USA; 4 Centre for Integrative and Applied Neuroscience, York University, Toronto, Canada; 5 Department of Biology, Centre for Vision Research, York University, Toronto, Canada

**Keywords:** Object vision, Cortex

## Abstract

How does the primate brain coordinate plasticity when learning to discriminate new objects? We measured consequences of object learning on inferior temporal (IT) cortex, a key waypoint supporting object recognition in the ventral visual stream, in male macaques. Neural activity in task-trained monkeys’ IT showed increased object selectivity, enhanced linear separability across objects, and more object-invariant representations compared to task-naïve monkeys. To model these differences, we developed a computational framework using anatomically-mapped artificial neural network (ANN) models of the ventral stream with various learning algorithms. Simulations revealed that gradient-based, performance-optimizing updates of ANN internal representations accurately approximated observed IT cortex changes. These models predict novel training-induced phenomena in the IT cortex, including changes independent of object identity and IT’s alignment with behavior. This convergence between empirical measurements and model predictions suggests ventral stream plasticity follows task optimization principles well-approximated by gradient descent, enabling accurate predictions about visual plasticity and generalization to test images.

## Abstract (web summary)

Whether sensory cortex reorganizes during object learning in adults remains unclear. Here the authors show that object training induces modest, task-aligned changes in macaque IT cortex that are well predicted by neural networks trained on the same task.

## Introduction

The ability to detect and discriminate different objects is a fundamental cognitive ability that enables primates to navigate and interact with their environment effectively. However, the kinds of objects that need to be mastered may be context-dependent (e.g., identifying safe fruits to eat in a new place). As a result, identifying new kinds of objects – even in adulthood – is pivotal for adapting swiftly to situational demands and thriving in unfamiliar environments. Recent years have seen much progress in computational modeling of the neural circuits that represent incoming visual images to, in turn, support the learned discrimination of objects (see ref. ^1^ for a recent overview). However, the field does not yet have computational models that explain if and how the ventral visual stream – and circuits beyond it – are modified by adult object learning.

Understanding how the brain changes to learn is a key goal in cognitive and systems neuroscience (e.g., ref. ^2^). However, studying the brain’s plasticity associated with a specific acquired behavior is a challenging problem that spans many spatial and temporal scales^3,4^. Despite much progress in developing various learning algorithms that optimize the task performance of ANNs^5–11^ and simulations on the type of neural measurements needed for distinguishing among algorithms^12^, the field currently lacks anatomically-precise mechanistic hypotheses and multi-level neural and behavioral measurements^2–4,6^ to advance our understanding of task-specific plasticity.

We reasoned that one promising strategy for identifying plasticity is to focus on measurements in brain areas with a well-established link to behavior. The inferior temporal (IT) cortex is one such area, as it has been extensively linked to core object recognition, the ability to rapidly recognize objects at the center of gaze^13^. IT responses exhibit selectivity to specific types of objects^14–17^ and object-like textures^18^, are predictive of human categorization behavior^19^, and causally influence categorization behavior across a variety of tasks^20–22^. While IT is thus a critical sensory waypoint for rapid object categorization behavior, it remains unclear whether changes in IT are necessary to enable the learning of new objects.

Indeed, there is evidence that plasticity in IT may not be necessary for discriminating new objects. For example, various object and categorical distinctions can be retrieved from the responses of IT neurons in monkeys unfamiliar with these specific objects or with categorization tasks^23–25^. Moreover, these IT-decoded choices can fully align with human decisions on the same tasks^19^. This suggests that the behavioral change associated with object learning could be explained as a new linear readout from IT. Thus, any plasticity associated with object learning would occur downstream of IT (i.e., closer to motor outputs^26^), in areas such as the caudate nucleus^27,28^, perirhinal cortex^29^ or in prefrontal cortices (^30–33^, but see ref. ^34^). Consequently, the higher visual cortex is deemed relatively stable after reaching adulthood^35–37^. Indeed, several studies have observed either small or no changes in IT with category training^35,38,39^. Consistent with this, a recent study modeling trial-by-trial object learning in humans^40^ demonstrated that specific learning trajectories are well-captured by dynamically updating linear decoders reading from a non-plastic IT-like feature representation of an ANN. Together, this suggests that combining versatile visual representations, as those present in IT, with plasticity in how this information is read out is sufficient for explaining how primates learn new object distinctions. From this perspective, task-specific plasticity in IT may be unnecessary or even counterproductive for task learning.

However, this view conflicts with numerous studies reporting training-induced changes in higher-level visual cortex response properties, including IT. For example, training macaques to categorize or discriminate objects can increase single-neuron selectivity for trained object discriminations relative to non-trained ones^25,31,41–44^, though some studies report small or no significant changes^35,38,39^. Even extensive training on orientation discrimination with faint gratings, stimuli typically associated with earlier visual processing, produces behaviorally relevant changes in posterior IT^45,46^. Similarly, human neuroimaging studies have revealed increased (and faster) selectivity for trained category distinctions in the visual ventral cortex^33,47–53^. However, these effects may depend on task engagement^32,54,55^. Even though there is substantial variability in the reported effect sizes^56^, and the impact of these effects on behavior is often difficult to establish, these findings raise the possibility that IT responses undergo changes to support new object discrimination behaviors, suggesting that plastic sensory representations may be necessary for object learning.

**Fig. 1:**
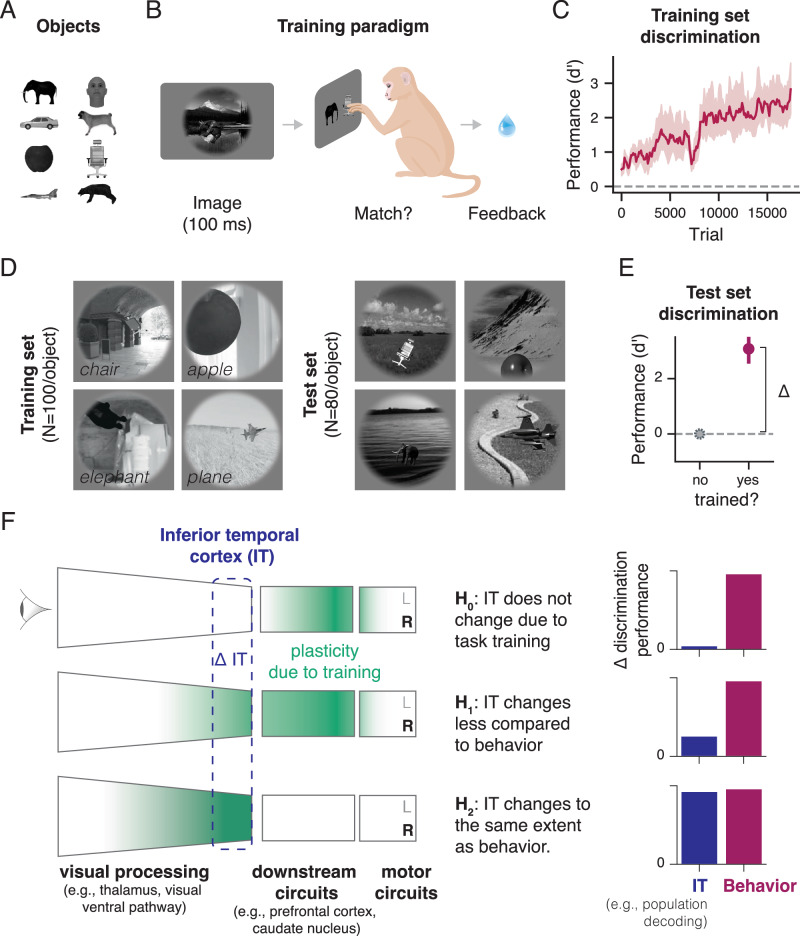
Does object training induce plasticity in sensory networks?. (**A**) During training, monkeys learned to match object images (with randomly generated views and backgrounds, N_images_ = 800) to one of eight possible objects (elephant, person, car, dog, apple, chair, plane, bear). **B** We trained naïve adult macaques to distinguish eight possible objects in a delayed-match-to-sample task, receiving rewards for correct choices trial-by-trial. Monkey visualization adapted from ref. ^131^ via SciDraw, licensed under CC-BY 4.0 (https://creativecommons.org/licenses/by/4.0/). **C** Object discrimination performance improves gradually during training. Performance is estimated using a sliding window of 500 trials for each object and averaged across subjects. The x-axis indicates the starting trial of each window. Shaded areas show 95% confidence intervals of the subject mean bootstrapped across the eight objects. See Supplementary Fig. S2 for details. **D** Example images from the training and test image datasets. Test images were not presented during training and were generated using new combinations of parameters (Supplementary Fig. S1). **E** After training, task-trained monkeys were proficient at performing this task on a new image set (N_images _= 640). We show the mean and 95% confidence interval (depicted as error bars) of the pooled behavior of all three subjects. Δ refers to the difference between the trained and naïve group. We assume chance performance (*d’* = 0) for the task-naïve group, and the dashed outline serves illustrative purposes. **F** How are sensory-motor circuits adapted to accommodate such new behavioral demands? Here, we focus on the inferior temporal cortex (IT) and formulate three hypotheses based on prior work. One hypothesis (H_0_) is that IT does not change and that any plasticity facilitating performance on the new task happens downstream of IT (and closer to motor outputs). This view considers IT to be a downstream readout region that no longer undergoes changes after development. Alternatively (H_1_), IT might change to support learning, yet those changes are not commensurate with those observed in behavior. Finally (H_2_), IT might reflect any change that is observable in behavior. A deeper shade of green in the drawn circuit schematic represents more plasticity to achieve the same performance. Source data files are provided for (**C**, **E**).

How might IT plasticity, in principle, support learning in the adult brain? Considering IT as part of a larger network of brain regions involved in object learning, rather than in isolation, it is crucial to examine how IT changes concurrently with other up- and downstream areas (Fig. 1F). This perspective aligns with viewing IT as part of a distributed optimization process, where changes across the network progressively reduce a loss function to optimize performance on a novel task (e.g., refs. ^2,6,57^). While there may be radically new tasks for which the entire network may need to adapt to optimize performance, in the adult brain, learning likely often recycles and slightly adapts pre-existing representations to produce new behaviors in more familiar settings. For instance, learning a new object is unlikely to induce changes in motor circuits or the retina but may necessitate adaptations in intermediate neural processing stages to ensure robust and reliable behavioral outputs. While there is extensive evidence that IT is a key node for object discrimination behavior, we do not yet know where IT lies on this continuum between stability and adaptability.

Focusing on IT and its link to behavior, we can discern three broad hypothesis classes (Fig. 1F). One possibility is that training-induced changes occur entirely downstream of IT (such as in the caudate nucleus or prefrontal cortex). In this scenario, IT is not expected to change (*H*_*0*_*: No plasticity*) despite animals successfully mastering new objects. Accordingly, IT’s responses already afford an effective readout of the trained objects in line with the idea that IT holds task-general representations. A second possibility is that IT changes with object learning, but only to a moderate degree. According to this view, IT holds more task-related information after training in service of the observed behavioral improvements (*H*_*1*_*: Moderate plasticity*), but the most significant changes might occur outside of IT. Finally, a third possibility is that IT is the key locus for change during discrimination tasks and thus any change observed in behavior is mirrored in IT (*H*_*2*_*: Peak plasticity*). In these latter two scenarios, learning new objects would thus alter IT’s representations to a different extent when compared to behavior.

Prior studies addressing these hypotheses have relied on single or fewer electrodes in small samples of animals, limiting the ability to detect training-induced changes in IT that may be subtle, distributed across many neurons^56^, and shared across individuals.

To address these limitations, we systematically tested these hypotheses within large-scale neural recordings performed across the task-trained and naïve macaque IT cortex. Examining two cohorts of adult macaques (3 subjects per cohort), each implanted with multiple chronic recording arrays, we could not only re-examine prior findings across hundreds of recording sites but also quantify differences at the population level. Our results revealed modest but robust differences in IT representations linked to object discrimination training, supporting *H*_*1*_: IT recordings from trained animals demonstrated more object-selective responses at the level of individual recording sites and improved separability for the trained objects at the population level compared to training-naïve IT recordings.

How do these differences in IT emerge, and are they indeed in service of the learned behavior? To answer these questions, we next developed an image-computable and anatomically-mapped modeling framework to test whether these training-related differences are consistent with performance-optimizing changes in a hierarchical sensory network. Using extensive ANN simulations, we demonstrate that the observed differences in IT are fully aligned with gradient-based updates to IT-like representations. Finally, we leveraged these functional models to predict two novel training-induced phenomena in the IT cortex, advancing our understanding of how IT supports learning to discriminate new objects.

## Results

As outlined above, in this study, we ask how plasticity across the macaque IT cortex enables learning of new object discrimination tasks. We aim to provide a systems-level answer to this question by examining changes in IT activity and the monkeys’ behavior in tandem with those observed in sensory-computable, mechanistic, architecturally referenced, and testable (SMART; see ref. ^1^) models of the ventral stream and object recognition behavior. To achieve this, we first compare the IT activity between groups of task-naïve and task-trained monkeys across an array of single-site and population-level metrics. Next, we ask whether updating the parameters of a leading set of SMART models of the ventral stream with a set of alternative plasticity mechanisms can account for these task-training-relevant neural differences. Finally, we leverage these models to make novel predictions about IT’s reconfiguration due to task training for untrained stimulus dimensions.

### Task training enhances task-relevant information in IT

Numerous studies have investigated how plasticity in IT is linked to category and object training (for a comprehensive review, see ref. ^56^). However, while some studies suggest that training-induced plasticity increases the selectivity of IT responses^25,31,41,43,44^, others only observed minor differences in selectivity^38^. Moreover, the magnitude of selectivity varies notably across studies^56^, highlighting the need to revisit this question with large-scale neural recordings^58^. Prior studies on IT’s plasticity have primarily focused on analyses of single-neuron properties, with limited attention to changes at the population level (but see ref. ^59^ for an analysis of information sparseness and object familiarity and a recent study by ref. ^60^). Consequently, it remains unclear how local changes in selectivity translate into increased information at the population level, which is available to downstream readout regions. To address these significant lacunae, we conducted tightly controlled experiments across two groups of monkeys (task-trained vs. naïve) and provided an in-depth analysis of how the neural representations vary between these groups as a function of task training.

**Fig. 2:**
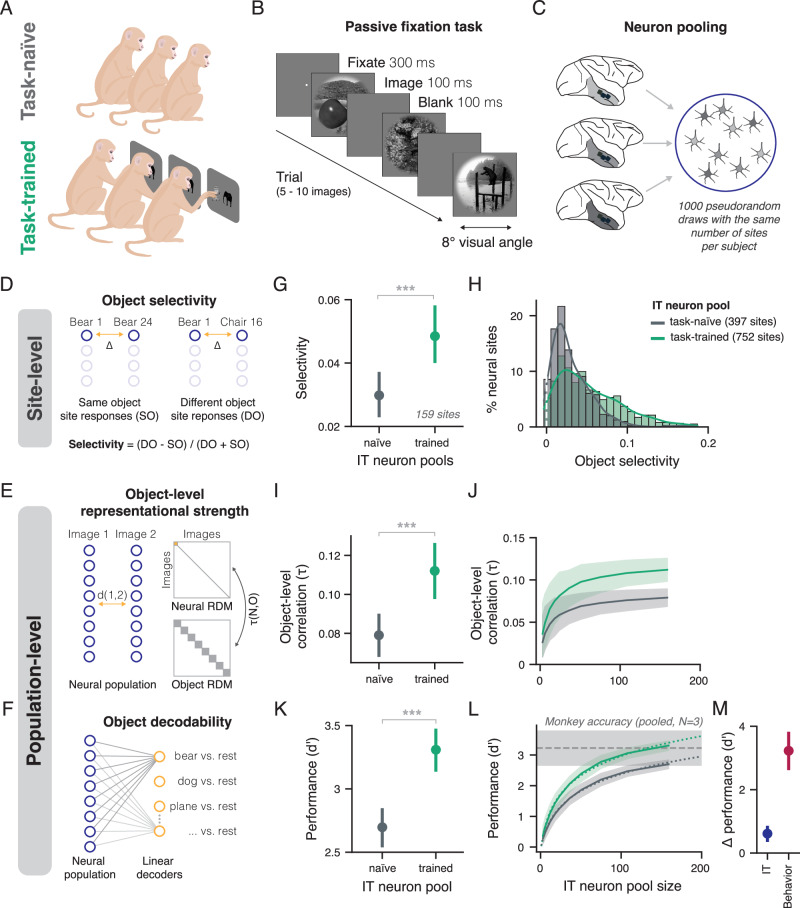
Object training enhances object-discriminating information in IT responses. **A** We recorded IT responses from task-naïve and -trained monkeys (N_subjects _= 3). Monkey visualization adapted from ref. ^131^ via SciDraw, licensed under CC-BY 4.0 (https://creativecommons.org/licenses/by/4.0/). **B** During neural recordings, subjects passively held fixation while viewing image sequences. **C** We pooled neural sites within a group using pseudo-random draws, ensuring an equal number of sites per monkey and comparable reliability across groups. **D** We assessed training-induced differences in IT using three metrics: Single-site object selectivity captures the extent to which a site’s response is modulated by specific objects. **E** Representational strength indicates whether a neuron pool’s representational geometry is well explained by object membership. **F** Object decodability assesses how readily the trained objects can be linearly separated in the neuron pools. **G** Trained IT pools exhibit a higher median selectivity compared to task-naïve pools. The error bars depict the 95% confidence interval (CI) of the median across draws. **H** This difference is also apparent in the distribution of object selectivity in the unpooled recording sites. **I** Trained IT pools have more object-related structure compared to naïve pools. **J** This effect is more pronounced in larger neuron pools. **K** Trained responses hold more discriminating information about the trained objects compared to naïve responses, yielding more accurate object decodes in held-out images. **L** The sampled IT population sizes are sufficient to approximate the trained monkey’s behavioral performance level. The horizontal shaded area shows the 95% CI of the trained monkeys’ mean accuracy (pooled, across 3 subjects) for the same images. We fit a log function to the trained IT decodes (dotted green line) to identify the crossing point of the trained decodes with the average trained monkeys’ behavioral accuracy (gray horizontal dashed line). **M** Comparison between differences in discrimination performance at the level of IT and behavior. IT differences show the same data as in K. Unless stated above, error bars and shaded areas depict the 95% confidence interval (CI) of the mean across draws and neuron pool comparisons are performed using 1000 draws with 159 sites for each group. *** represents *p* < 0.0001 in a two-sided two-sample bootstrap test. Source data are provided for G-M.

We compared groups of task-naïve and task-trained monkeys (*N* = 3 each). Both groups learned to hold fixation to enable neural recordings. The task-trained group, in addition, was trained to discriminate objects (Fig. 1A, D) using operant conditioning in a delayed-match-to-sample task (see Fig. 1B for a schematic). Familiarity with the task structure alone was insufficient to solve this task: Subjects, who were already familiar with the task structure, displayed low performance at the onset of the object training (*d’* = 0.50) and only gradually improved their discrimination abilities over the course of the training (*d’* = 2.82, Fig. 1C and Supplementary Fig. S2), thus indicative of object-specific learning. After training, all task-trained subjects demonstrated high-performance levels on unseen images (Fig. 1E). To assess the effects of task training on IT, we compared multi-unit IT responses recorded from task-naïve subjects with those of task-trained ones (Fig. 2A). All recordings were performed using chronically implanted Utah arrays (2-3 arrays per monkey; see Supplementary Fig. S3 for array locations). During recording, monkeys passively fixated on a sequence of naturalistic images, each containing one of eight objects (Fig. 2B). We presented the images in a random order, briefly (100 msec), and at the center of gaze (central 8°). To achieve comparable recording quality in both groups, we pooled IT sites (with statistically indistinguishable response reliabilities; see “Methods”) from each group (task-trained vs. naïve) by performing 1000 pseudo-random draws that ensured an equal number of recording sites per monkey (Fig. 2C). Finally, we analyzed the obtained IT neuron pools for differences in task-related information using three complementary metrics: single-site object selectivity, object representational strength, and linear object decodability.

As a first step, we compared both groups with regard to site-level response selectivity, a standard analysis for assessing training-induced neural changes^25,31,41,44^. To replicate earlier findings, we computed site-specific selectivity by comparing the average pairwise response differences between images displaying the same object to those showing different objects (see “Methods” and Fig. 2D for a schematic). In line with earlier studies, sites from task-trained responses showed a consistently higher degree of selectivity than those from the task-naïve pools (Fig. 2G, H). This training-induced difference corresponded to a 63% increase in median selectivity (Trained: 0.048 (median), Naïve: 0.029, Difference: 0.019, CI_Difference_ (95%) = [0.008, 0.029], *p* < 0.001). This suggests a notable modulation of local firing rates due to task training and replicates prior claims in our larger dataset.

To address whether these site-level changes were also reflected in how task-related information is represented at the population level, we performed two complementary analyses. First, we performed representational similarity analysis (RSA^61^) to track whether task training was associated with an increased clustering according to the trained objects. Second, we fitted linear decoders to estimate the overall information about the trained objects for downstream readout.

We chose RSA for quantifying training-induced changes since it allows for quantifying changes in local geometry with regard to a specific conceptual model (i.e., the grouping of different views of the same objects). Moreover, RSA does not require any fitting procedure and thus provides an estimate of task-related information in the native data space (Fig. 2E). In agreement with the selectivity analysis, we found that task-trained IT pools were more object-specific (i.e., showed lower dissimilarities within object views as compared to between different objects) than task-naïve pools (Fig. 2I, Trained: 0.112 ± 0.007 (mean ± SD), Naïve: 0.079 ± 0.005, Difference: 0.033 ± 0.009, CI_Difference_ (95%) = [0.015, 0.05], *p* < 0.001). This difference was enhanced in larger neuron pools (Fig. 2J) and corresponded to a 41% increase in the largest neuron pools (159 sites).

Finally, we asked whether these differences in local geometry translate into an overall increase in information available for downstream regions via linear read-out. We assessed this by training linear classifiers to predict the object labels (using cross-validation) based on the neural responses from both groups (Fig. 2F). We observed that object predictions from the trained IT populations showed a significantly higher accuracy compared to naïve IT populations (Fig. 2K, Trained: 3.31 ± 0.08 (mean ± SD), Naïve: 2.70 ± 0.08, Difference: 0.612 ± 0.108, CI_Difference_ (95%) = [0.398, 0.811], *p* < 0.001). Given that we could only record from a limited sample of neural sites (with respect to the total number of neurons in IT), we approximated how our estimated decoding accuracy differences scale with the number of sampled sites. As for representational strength, we observed that the object information increased in larger neuron pools (Fig. 2L, 22.8% increase at 159 sites). Of note, despite the significant difference between the two groups, we observed that both IT populations could reach the behavioral performance levels shown by the trained monkeys (73 and 142 sites for the naïve and trained sites, respectively). This suggests a quantitative scaling effect of training that enhances the readout for downstream regions rather than a qualitative change due to task training (i.e., an effect through which performance only becomes possible with training). In other words, in both pools, IT holds sufficient information to guide the observed behavior, but in the trained IT recordings, the same level of performance is achieved using fewer sites. Taken together, these results complement the representational strength analysis and point to the fact that task training affected the encoding of visual information in IT at the population level, enabling a more efficient readout of task-relevant information for downstream areas.

Do these training-induced differences exceed what would be expected from interindividual variation alone? That is, could the observed effects be driven by differences between a few subjects, irrespective of their training history? To test whether our observed training differences are robust to interindividual variation, we implemented two additional analyses. First, we qualitatively replicated each of our training effects for each animal. Supplementary Fig. S6 demonstrates that the overall direction of effects is largely consistent with the group-level results shown in Fig. 2, particularly for the decoding metric, though there is variability across subjects for the other metrics. Second, we assessed these qualitative observations statistically using a hierarchical permutation bootstrapping test. This test not only captures the variability between groups but is also sensitive to variability within groups (see “Methods” - Statistical Analysis). We observed that the effects linked to task training were significantly greater than what would be expected from interindividual variation for selectivity (*p* = 0.003, Supplementary Fig. S7A), representational strength (*p* = 0.041, Supplementary Fig. S7B), and object decoding (*p* = 0.002, Supplementary Fig. S7C). These results support that the observed differences are attributable to task training rather than pre-existing individual variation, validating our approach of pooling data across subjects within each condition.

**Fig. 3:**
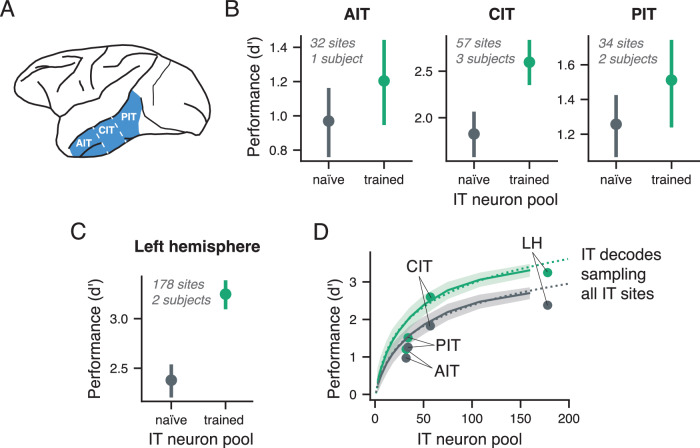
Training effects in object decoding scale with dataset size, not anatomical location. Are the differences between trained and naïve monkey IT responses due to differences in the anatomical locations of the recording arrays? **A** To investigate this possibility, we classified the arrays from all subjects as either posterior, central or anterior IT (PIT, CIT, AIT, respectively). **B** Recordings from trained animals in all subregions of IT yield higher decoding accuracies compared to recordings from naïve subjects. As in our main analysis (cf. Figure 2K), recordings were pseudo-randomly sampled to counterbalance for differences in reliability and subject counts between groups. **C** Are differences due to imbalanced sampling across the hemispheres? Repeating our analysis only in the left hemisphere, we again observe an advantage of trained IT responses. **D** The difference in effect sizes across analyses is driven by the number of available sites. Lines show decoding accuracies obtained when subsampling the entire IT dataset (irrespective of an array’s anatomical location), and the dashed line shows a fitted function to that data. The site counts and mean estimates from panels (**B**, **C**) are annotated for comparison. In all panels, we show the mean and 95% confidence interval (depicted as error bars and shaded areas) of the mean across draws and all pool comparisons are performed using 1000 draws with the annotated number of sites. Source data are provided for (**B**, **C**).

Thus far, we pooled all available IT recordings within each group, irrespective of anatomical location, to maximize our signal-to-noise ratio. However, this pooling might introduce unintended differences between the task-naïve and trained groups if the recorded sites differ anatomically. Given that neural responses vary systematically along the posterior-to-anterior axis of IT^62–64^ our observed group differences could result from anatomical variation rather than task training itself. To directly test for this possibility, we subdivided the recording arrays into distinct IT subregions (AIT, CIT and PIT, Supplementary Fig. S3) and repeated our object decoding analyses separately within each subregion. We observed that responses in all subregions contained more information on average, about object identity in the task-trained compared to the naïve group (Fig. 3B). Still, the quantitative differences were only statistically different in central IT where many recording sites were available (AIT: Difference = 0.234 ± 0.159, CI_Difference_ (95%) = [− 0.056, 0.534], *p* = 0.16, 24.2% increase; CIT: Difference = 0.770 ± 0.166, CI_Difference_ (95%) = [0.446, 1.070], *p* < 0.001, 42.2% increase; PIT: Difference = 0.254 ± 0.149, CI_Difference_ (95%) = [− 0.050, 0.537], *p* = 0.112, 20.2% increase). In addition, hemispheric differences in array locations could be another driver of training-unspecific differences in our results. Repeating our analysis for the left hemisphere only, we again observed a pronounced increase in task-related information in the task-trained IT responses (Fig. 3C, Difference = 0.870 ± 0.103, CI_Difference_ (95%) = [0.676, 1.070], *p* < 0.001, 36.6% increase). Lastly, we investigated whether variability in effect sizes across IT subregions was attributable to differences in the number of available recording sites. Indeed, all anatomically-constrained training effects were well predicted by the effect of the number of sites in the entire dataset, suggesting that the number of sites, rather than anatomical location, was the stronger determinant of observed training effect sizes (Fig. 3D).

**Fig. 4:**
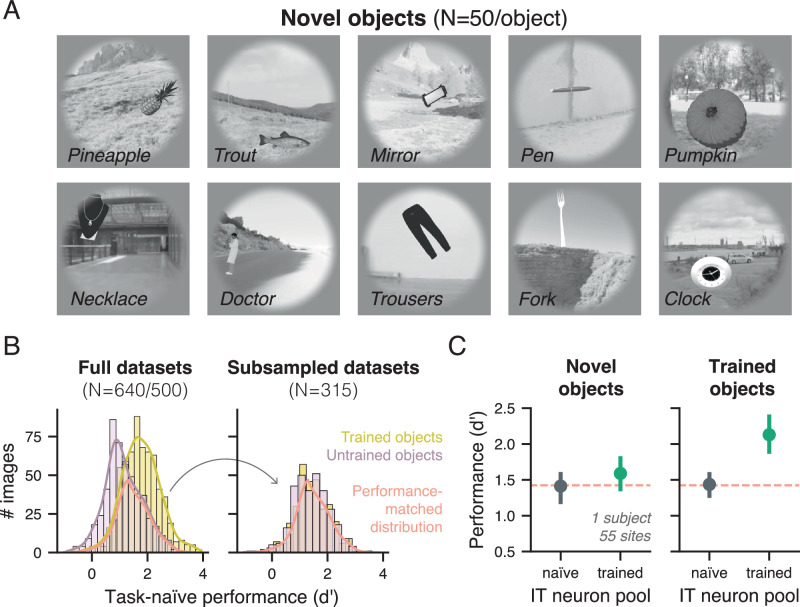
Task training does not increase decoding for novel objects. **A** To test whether training-related differences also affect the information about novel objects, we repeated our decoding analysis using a different image dataset consisting of 10 novel objects (50 views each) that were not part of the training objects. Since the original task-naïve subjects were not tested with these novel objects, we recorded data from a new task-naïve individual (monkey P; see “Methods”) for this comparison. For the task-trained condition, we therefore also selected one individual from the trained group and matched the reliability of both datasets based on our standard procedure (see “Methods”). **B** Controlling for dataset difficulty. To enable a comparison between datasets, we matched the difficulty of both stimulus sets based on the decoding accuracy in the naïve responses. This yielded reduced datasets (N_images _= 315 for each stimulus set) matched in their naïve decoding performance and dataset size. **C** Decoding performance for trained versus novel objects. Even at the single-subject level, we observe improved decoding for the trained objects in the task-trained recordings. In contrast, we do not observe better decoding for the novel objects due to task training. Error bars show the 95% CI of the mean based on bootstrapping across neuron pools (1000 draws). Source data are provided for (**C**).

To what extent are the observed training effects specific to images depicting the objects used during training? Repeating the decoding analysis using another image dataset displaying novel objects (10 objects, 50 images each, Fig. 4A) and another task-naïve subject (monkey P), we see that objects not featured during training did not elicit significantly different decoding accuracies (1 subject per group, 55 sites, Difference (mean ± SD) = 0.176 ± 0.147, CI_Difference_ (95%) = [− 0.118, 0.474], *p* = 0.212, 12.47% increase, Fig. 4C, left panel). In contrast, the dataset showing the trained objects to the same subjects did produce significant differences in such matched conditions (Difference (mean ± SD) = 0.693 ± 0.147, CI_Difference_ (95%) = [0.411 − 0.976], *p* < 0.001, 48.3% increase, Fig. 4C, right panel).

In sum, task-trained IT responses displayed more object-selective site responses, more task-aligned representations at the encoding stage, and more linearly decodable information available for downstream readout. These differences are thus inconsistent with the view that IT does not change with task training (i.e., we can reject *H*_*0*_). How do these differences at the level of IT, compared to those observed in behavior? If training-induced behavioral differences are directly passed down from IT, we would expect these differences to have similar magnitudes (*H*_*2*_). Instead, Fig. 2M demonstrates that the differences at the level of IT are dwarfed by those apparent in behavior (and thus reject *H*_*2*_).

### Training hierarchical sensory models as hypotheses of IT plasticity

Our empirical results, as shown in the previous section, demonstrate that while task training induces task-relevant changes in IT responses, those differences appear insufficient to account for the differences in behavior (Fig. 2M). These findings falsify two initial hypotheses (Fig. 1F), which predicted either no changes in the IT cortex upon task learning (*H*_*0*_) or equivalent change to that observed in behavior (*H*_*2*_). If IT changes upon learning, what determines the magnitude of these differences? What are the constraints under which such moderate differences may be observed in a neural network serving the function of object recognition? How do these task-relevant differences in IT and the differences in behavior (a.k.a. learning) co-occur? To address these questions, we propose training (SMART) ANN models as an alternative hypothesis space to rigorously link differences in behavior to those observed in IT. Thus, we aim to provide quantitative predictions of the magnitude of IT’s training-induced plasticity along task-relevant metrics consistent with the observed task performance.

**Fig. 5:**
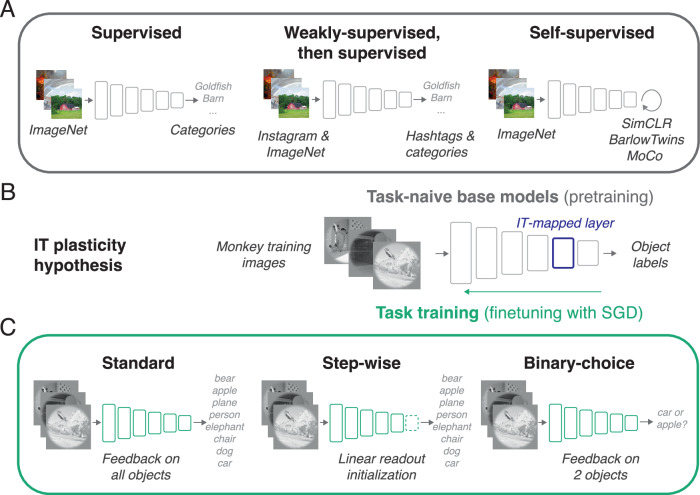
Task-optimized ANNs as IT plasticity hypotheses. We propose task optimization in ANNs as an alternative hypothesis space for IT plasticity. **A** Specifically, task-naïve IT may be readily approximated by various deep convolutional neural networks pre-trained on large-scale image databases with supervised, semi-supervised, and self-supervised objectives. We refer to these as task-naïve base models. **B** To obtain functionally relevant model hypotheses, we optimize each of these base models on the images and objects used during monkey training. Before optimization, we identify the model layer that best encodes IT responses in the task-naïve group (IT-mapped layer, Supplementary Fig. S9) and establish how many layer units are needed to approximate the information contained in a single IT recording site (Supplementary Fig. S10). (**C**) We apply three training strategies to every base model and repeat this procedure 20 times with various hyperparameter combinations (learning rate, regularization strength, batch size, etc.). The standard training strategy refers to replacing the final model layer with a randomly initialized layer with 8 object nodes and then finetuning all model weights simultaneously. For the step-wise training, we first train the linear readout layer (with the remaining network weights frozen) and then finetune the remaining network weights. This mimics a rapid and slow phase of plasticity. For binary-choice training, we mimic the sparse binary feedback in the monkey’s delayed match-to-sample task by updating the model weights for the target class and a randomly chosen distractor class.

We assumed that a deep convolutional neural network pre-trained on a large-scale image database could serve as a proxy for a task-naïve monkey’s visual system (referred to as base models), akin to evolutionary and developmental influences providing strong priors for the adult visual system. We adopted 13 pre-trained ANNs (base models) with different model architectures and optimization objectives, including supervised, weakly supervised, and self-supervised training objectives (Fig. 5A, see “Methods”). For each of these base models, we performed a two-step IT-to-model layer mapping procedure. First, we identified the model layer in all base models that best encoded task-naïve IT responses (see Supplementary Fig. S9 for the chosen layer and position in every base model). Second, we estimated how many ANN units in that layer corresponded to a single neural recording site (Supplementary Fig. S10). This gave an estimated number of units in a model’s IT-mapped layer that needed to be randomly sampled to obtain a similar level of decodable information as present in the task-naïve IT recordings.

With this initial mapping between task-naïve IT and base model in place, we then trained (i.e., finetuned) these base models using the same training images and object labels used to train the monkeys on these tasks (100 images per object, 800 images in total). To account for the possibility that certain learning strategies may be more plausible than others, we included three different training strategies: a standard finetuning approach, a step-wise finetuning strategy with an optimized initialization of the object readout layer, and a binary choice training that mimicked the choice and feedback structure of the delayed-match-to-sample task used during monkey training (Fig. 5C, see “Methods” for further details). During training, all model weights were free to change (i.e., no frozen parameters). In total, we trained 660 models while varying various hyperparameters (from 11 base models since 2 base models already failed during the task-naïve IT mapping stage).

### Task-optimized ANNs reproduce IT’s task-training-induced differences

**Fig. 6:**
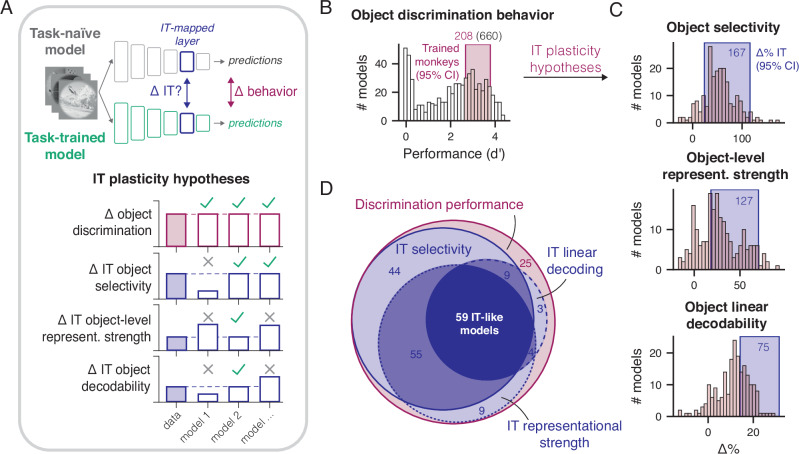
Various task-optimized ANNs reproduce IT’s task-training differences. **A** upper panel, After training, all models showed performance improvements. How did these changes relate to the changes in their IT-mapped layer? (**A**, lower panel) To create a parallel to the empirical data, we only include models that performed on par with the trained monkeys after training (magenta-outlined bars). We then analyze the IT-mapped layers for changes due to task training by comparing the task-trained models to their respective task-naïve base models. Specifically, we compare the IT-mapped layers (task-naïve/pretrained vs. task-trained) using the same metrics as those adopted during neural data analysis (Fig. 2). This step provides specific predictions of how any model’s IT-mapped layer’s activations were modified during task training to optimize its task performance. We expect a good model to reproduce the magnitude of IT changes for all metrics (i.e., three check marks). **B** Only some task-trained models perform on par with trained monkeys. Magenta bands show the 95% CI of the pooled monkey performance on held-out test images (*N* = 640). Training-specific results are shown in Supplementary Fig. S12A. We only evaluate models with matching performance as relevant models of IT plasticity. **C** For all metrics, we see a significant proportion of models displaying change magnitudes akin to those in IT recordings. The x-axis shows the mean estimate of changes across unit draws for every model (*N* = 208). Most, but not all, models show an increase in these object-related metrics due to task training (i.e., distribution is shifted to the right of 0). Blue shaded areas depict the 95% confidence interval of observed changes in IT (across 1000 draws). The annotated numbers describe how many models matched the neural data in their magnitude and direction of change for a given metric. **D** Agreement across metrics. For every model, we ask whether the same model matches the neural data for multiple metrics. By design, all models match the object discrimination performance (magenta circle). The darkest shade of blue shows the proportion of models matching all metrics. The medium and lighter shades of blue depict the proportion of models matching two or only a single metric. Source data are provided for (**B**–**D**).

As a first step, we reasoned that any eligible model of IT plasticity should perform on par with the trained monkeys (Fig. 6A). Therefore, we tested all trained models for generalization on the monkey’s test images (*N* = 640, not included in the model’s training dataset). We found that all training approaches and most base models yielded models that performed equally well as the pooled trained monkeys (Fig. 6B and Supplementary Fig. S12A). This provided us with 208 model hypotheses and excluded under- and over-performing models from further analyses (~ 32% remaining, 208/660 models). We considered these models as plausible accounts of how the observed changes in behavior could be implemented by a system learning a new task.

For each of the considered models, we then applied the same three metrics used to characterize changes in the IT recordings (i.e., change in selectivity, representational strength, and decoding, cf. Figure 2). A good model of IT plasticity should ideally match the changes in all three metrics (Fig. 6A, bottom panel). On the other hand, a model that does not resemble IT along any of the metrics is likely not a good model of IT plasticity. Finally, since our evaluated metrics possibly capture different aspects of training-related changes (e.g., local vs. global changes), some models may only resemble IT in one metric but not in another. Such a pattern might reflect that each of these metrics might characterize different aspects of the neural differences.

For all metrics, a substantial proportion of tested models showed the same level of changes upon object learning in their IT-mapped layers as observed in IT (Fig. 6C, 80, 61, and 36% for object selectivity, representational strength, and decodability, respectively). Inspecting the overall model distribution suggests a general alignment in terms of magnitude with IT changes. This alignment was particularly pronounced for changes in selectivity. This suggests that training ANNs on the same task mastered by the task-trained monkeys and observing the comparable levels of task proficiency, yields, in most cases, models that mimic the task-related differences observed in IT cortices.

Were there any models that exhibited IT-like changes across all metrics? Among all 208 assessed models, we identified 59 models that were indistinguishable from the neural data for all tested metrics (referred to henceforth as “IT-like” models). Moreover, Fig. 6D highlights how one metric can be considered more sensitive to identifying IT-like models. For example, observing a match in object decoding appeared to be a good predictor for a match in the remaining two metrics. On the other hand, many models resembled IT’s changes in selectivity, yet these models did not necessarily also match IT’s changes in population-level metrics.

To test whether the observed changes are specific to the features of IT-mapped layers, we performed a control analysis where we replaced the IT-like ANN layer with other earlier and late control layers (with less IT-like encoding, sampled throughout the network for a single architecture). For selectivity and decodability, and less so for representational strength, we observed that IT-like layers most aptly captured the neural differences (Supplementary Fig. S11A–C).

Can we identify factors that explain why one model better accounted for the changes in IT than another? To investigate this, we returned to the variations introduced during task training (i.e., base models and task training strategies, Fig. 5). We assessed whether any training strategy or base model was associated with a higher proportion of IT-like models (i.e., a match in all three metrics). Despite the differences in how the networks were finetuned, we observed that all three training strategies produced IT-like models in a relatively comparable proportion (Supplementary Fig. S12B), thus suggesting that the larger changes associated with task optimization using gradient descent led to the IT-likeness. In the tested base models, we further observed that ResNet-style architectures were more effective at producing IT-like models compared to VGG-style and AlexNet architectures, pointing to a potential benefit of skip connections for producing IT-like models. We considered base models with different pre-training objectives, reasoning that models pre-trained using a self- or weakly-supervised objective could be a more appropriate starting point for IT-like task training. In particular, adult naïve monkeys will have received very little explicit (i.e., supervised) feedback about object categories^65^. Thus, adopting a base model exposed to a similar feedback regime as the task-naïve monkeys could be critical for explaining the observed differences in IT. Against our expectations, we observed that self-supervised and supervised pre-trained models produced relatively comparable proportions of IT-like models (except ResNet50 pre-trained with MoCov2). For the two tested weakly-supervised models, we could not identify a unit-mapping scaling that approximated naïve IT recording sites. These results indicate that the exposure to category signals during pre-training does not result in less IT-like models. Collectively, this analysis suggests IT-like models emerge in an interplay between a base model’s naïve state, the applied training strategy, the random initialization of the output layer and the hyperparameters used in training.

**Fig. 7:**
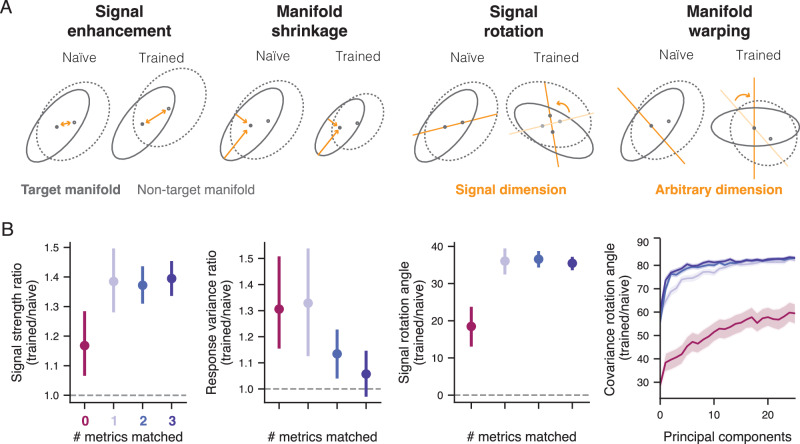
Identifying geometric properties that distinguish IT-like models. **A** Schematic illustration of learning mechanisms on object manifolds. To understand what distinguishes models that match IT plasticity patterns from those that do not, we analyzed different geometric factors driving manifold changes during training using metrics from ref. ^66^. Training can increase task-specific information through four mechanisms: (1) Signal enhancement (SE): increasing the distance between target and non-target manifold centroids; (2) Manifold shrinkage (MS): reducing manifold size, thereby tightening clusters of instances from the same object; (3) Signal rotation (SR): rotating manifolds to change the relative orientation of centroids; (4) Manifold warping (MW): altering the covariance structure across dimensions. Solid ellipses represent target object manifolds; dashed ellipses represent non-target manifolds (including all other objects). **B** Comparison of geometric metrics across models grouped by IT alignment (x-axis: number of IT metrics matched, from 0 to 3). All four metrics distinguish models matching no IT metrics from those matching some or all. SE, SR, and MW primarily separate IT-unlike from IT-like models, showing relatively binary patterns. MS reveals a more graded relationship: IT-like models maintain approximately constant manifold variance across training (MS ratio ≈ 1), whereas less IT-like models show increased manifold size. We show the mean and error bars as CI (95%) of the mean estimates across models (N_0match_ = 25, N_1match_ = 56, N_2match_ = 64, N_3match_ = 59 models for each level of matched metrics). Source data are provided for (**B**).

To further probe what distinguishes IT-like models from the alternatives, we conducted geometric analyses of the representational manifolds before and after task training (Fig. 7). Following metrics proposed by Cheng et al.^66^, we quantified changes in four distinct geometric properties that can each independently contribute to increased task-relevant information: (1) signal enhancement (increasing distance between target and non-target manifold centroids); (2) manifold shrinkage (tightening the clustering of images within each object category); (3) signal rotation (repositioning the manifold centroids relative to each other); and (4) manifold warping (altering the covariance structure across dimensions, Fig. 7A). We compared these metrics across models grouped by their degree of IT-likeness (i.e., the number of IT metrics matched). All four metrics qualitatively distinguished IT-unlike models (matching 0 metrics) from IT-like models (matching all 3 metrics). That is, signal enhancement, signal rotation and manifold warping appeared associated with meeting at least one IT metric. Manifold shrinkage showed the most systematic relationship with IT-likeness, revealing graded differences across intermediate levels (Fig. 7B). Specifically, the most IT-like models maintained relatively stable manifold variance between their naïve and trained states, whereas models matching fewer IT metrics exhibited increased variance after training, indicating that images formed larger, more dispersed clusters following task training. This finding provides a complementary perspective on our earlier results: increases in neural selectivity alone, achieved potentially by signal enhancement or rotation, are insufficient to reproduce all IT changes; rather, training must enhance discriminability via subtle and distributed changes across the population (e.g., via manifold shrinkage) to parallel the differences observed in IT.

### IT-like models best predict differences along untrained dimensions in IT

**Fig. 8:**
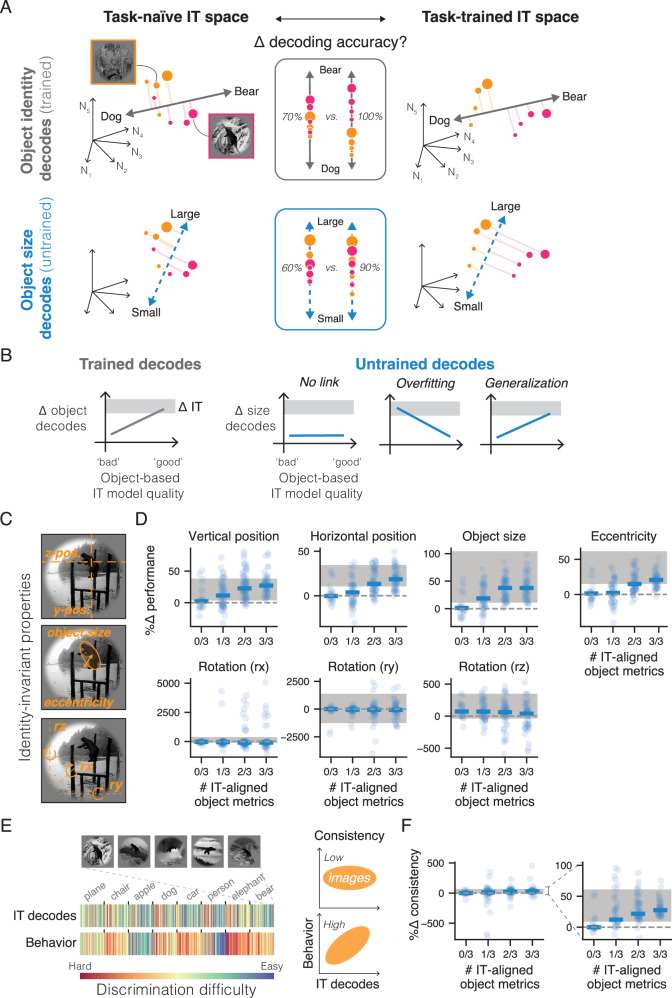
IT-like models generalize to changes along untrained readout dimensions. **A** We investigate how object training alters IT representations using linear probes. Any image is encoded in a high-dimensional IT space. Consider 8 images encoded by N neurons. Orange and pink circles represent dog and bear images, respectively, and circle size indicates object size. Arrows show readout dimensions for different decoding tasks. The upper panels show how object identity decoding improves after training. The lower panel applies an untrained size decoder. While size was not reinforced during training, the IT encoding of this dimension still might change. **B** We rank models according to their match with IT on object identity-related metrics (left). Can good models (models matching all metrics) predict changes for other untrained readout dimensions? We hypothesize three scenarios: (1) There could be no link, indicating that a model’s fit based on object metrics does not transfer to other dimensions. (2) There could be a negative link, indicative of overfitting, meaning that the best models are the worst for predicting differences in other dimensions. (3) A positive link suggests generalization to other untrained dimensions. **C** Beyond an object’s identity, images varied along properties such as an object’s location, its size, and pose. **D** Most IT-like models best predict identity-invariant changes. The x-axis spans four groups of models with increasing IT-likeness as assessed via a match to IT metrics (*N *= 25/56/64/59 models per level of matched metrics, cf. Figure 6D). Shaded gray bars show the 95% CI of the changes in IT. Dots represent individual models. Blue horizontal bars depict the median change across models. **E** Do training-related IT changes increase the alignment with image-specific (trained) behavior? To obtain image-specific difficulty estimates for the IT recordings, we fit linear decoders to the neural recordings and estimate image-level decoding accuracy (*d’*, see “Methods”). We can estimate the same metric based on the monkeys’ choices during a behavioral experiment. **F** IT-like models best predict increases in training-induced IT-behavior consistency. The right panel is a zoomed-in version. Figure elements are defined as in D. Source data are provided for (**D**, **F**).

Beyond a fit to the training-related differences, a good model of IT plasticity should also generalize and predict novel phenomena beyond the scope of training. In the previous section, we identified models fully mimicking the effects of object discrimination training on both behavior and IT responses. Yet, it remains possible that such IT-like models exclusively mimic object-specific changes (because they were selected for it, cf. Fig. 6) but fail to predict anything beyond the direct effects of training. A way to further assay the quality of our models is thus to probe whether IT-like models generalize to other domains or dimensions that were not reinforced during training. Examples of such domains are predicting an object’s position, eccentricity, rotation, and an image’s perceptual difficulty reflected in the trained monkeys’ behavior. Examining decoding differences along such dimensions is meaningful since the effects of object discrimination training (e.g., a larger separation between images belonging to separate objects, Fig. 8A, upper panel) are likely also to affect other stimulus-related dimensions (e.g., an object’s size, Fig. 8A, lower panel). Such untrained dimensions can thus serve as additional probes for examining if two systems underwent a functionally similar change (from the perspective of downstream readout regions from IT). By leveraging a variety of these training-unrelated decodes, we can thus put our identified IT-like models to the critical test: If such models do not only predict IT’s object-related differences but also generalize to a variety of other untrained decodes, this provides strong evidence that these models capture more general aspects of training-induced IT’s change (Generalization, Fig. 8B). Alternatively, suppose a model’s object-specific IT-likeness shows no correlation or even an inverse relationship with its ability to decode untrained image aspects. In that case, this suggests that the model captures only narrow, object-specific differences. This latter case could be considered a form of overfitting to the empirical benchmarks tested thus far (No link or overfitting, Fig. 8B). In what follows, we tested the ability of IT-like models to generalize to these untrained readout dimensions. We also evaluated less IT-like models as control conditions to understand whether a model’s generalization directly stems from its IT-likeness.

A prior study^67^ demonstrated that object-orthogonal properties, including an object’s location, size, position, and rotation from a canonical view (Fig. 8C), can be linearly decoded from task-naïve IT responses. The accuracy of these IT-based decodes was, in turn, correlated with that of human judgments, suggesting that IT may serve as a readout region for such judgments. This makes these dimensions ideal candidates for assessing generalization in the IT-like models. In particular, we expect that models with the closest alignment in object-related IT metrics will also most accurately predict the training-induced IT differences for each of these object-invariant dimensions. To test this, we leveraged that our experimental images were created by rendering a 3D object with varying poses, locations, and sizes (Supplementary Fig. S1). Consequently, we had access to the ground-truth values of these image properties and could thus quantify how accurately they could be decoded from IT responses. Concretely, we fit cross-validated linear decoders to the pooled responses in both groups (similar to object decoding) to measure linear decoding accuracy (see “Methods”).

For the IT recordings, we found that object training significantly increased identity-invariant information about an object’s position, size, and eccentricity (Vertical position: Difference (mean ± SD) = 0.100 ± 0.037, CI_Difference_ (95%) = [0.023, 0.168], *p* = 0.01, 20.5% increase; Horizontal position: Difference = 0.120 ± 0.030, CI_Difference_ (95%) = [0.066, 0.181], *p* < 0.001, 21.3% increase; Object size: Difference = 0.133 ± 0.048, CI_Difference_ (95%) = [0.035, 0.224], *p* < 0.001, 49.8% increase; Eccentricity: Difference = 0.155 ± 0.041, CI_Difference_ (95%) = [0.079, 0.241], *p* < 0.001, 34.13% increase) but not about its rotational attributes (Rotation rx: Difference (mean ± SD) = 0.034 ± 0.053, CI_Difference_ (95%) = [− 0.067, 0.142], *p* = 0.532, 28.5% increase; Rotation ry: Difference (mean ± SD) = 0.032 ± 0.050, CI_Difference_ (95%) = [− 0.062, 0.137], *p* = 0.536, 355.2% decrease; Rotation rz: Difference (mean ± SD) = 0.055 ± 0.048, CI_Difference_ (95%) = [− 0.035, 0.150], *p* = 0.254, 57.6% increase, Supplementary Fig. S13A). Good models of IT plasticity were thus expected to mimic differences for some dimensions (object position, size, and eccentricity), yet not for others (rotational properties). Then, we performed the same analysis in each of the models’ IT-mapped layers. We evaluated both IT-like models (i.e., matching all three object-related metrics) as well as less IT-like models with either two, one, or no match with any of the object-related metrics (Fig. 6D) to isolate the effect of IT-likeness. For the properties that significantly differed between task-naïve vs. -trained IT recordings (i.e., object location, size, and eccentricity), we observed that IT-likeness introduced significant differences between model groups (upper panel, Fig. 8D, Vertical position: *H(3)* = 38.34, *p* < 0.001, *ε²* = 0.173; Horizontal position: *H(3)* = 51.03, *p* < 0.001, *ε²* = 0.235; Object size: *H(3)* = 45.03, *p* < 0.001, *ε²* = 0.206; Eccentricity: *H(3)* = 50.83, *p* < 0.001, *ε²* = 0.234) with IT-like models always producing a larger increase in accuracy compared to IT-unlike models. Moreover, an increasing degree of IT likeness was associated with a better match with the empirically observed differences. For properties that did not differ between the task-trained vs. task-naïve neural recordings (i.e., rotations), we observed that all model groups provided good approximations of the null effect (lower panel, Fig. 8D). We also did not observe a significant difference between IT-like and IT-unlike models for these properties (Rotation rx: *H*(3) = 7.51, *p* = 0.057, *ε²* = 0.022; Rotation ry: *H(3)* = 0.32, *p* = 0.957, *ε²* = −0.013; Rotation rz: *H(3)* = 3.74, *p* = 0.290, *ε²* = 0.003). This pattern of results supports the notion that IT-like models capture more general training-induced differences that extend beyond object-related metrics.

Within the IT-like models, how specific is the ability to generalize to the IT-mapped layer? To test this, we repeated our analysis comparing the IT-mapped layer to an earlier and a later control layer within one base model group (Supplementary Fig. S11). For the majority of object-invariant properties, we indeed find that IT-mapped layers tend to better predict IT’s differences along untrained dimensions than the control layers (Supplementary Fig. S11D).

In addition to these object identity-invariant properties, a prior study demonstrated that an image’s behavioral difficulty is readily decodable from IT responses^19^. That is, the degree to which an image’s object is linearly distinguishable from IT responses is linked to the behavioral difficulty of picking the correct target canonical view when presented alongside various distractor objects (Fig. 8E). While we have shown in our prior analysis that there is an overall increase in mean decoding accuracy (Fig. 2K), we do not yet know how the link between image-by-image decoding patterns differs with task training and how these IT-decoded image-by-image patterns, in turn, compare to behavioral patterns. Importantly, we can adopt this behavioral metric as another readout dimension to further probe the IT-like models for generalization. As with the object identity-invariant properties, we again expect that IT-like models should most accurately predict any effects of task training, whereas less IT-like models are not expected to predict these differences.

In the IT recordings, we observed a significant increase in behavioral consistency between task-trained and task-naïve IT recordings (Trained: 0.56 ± 0.03 (mean ± SD), Naïve: 0.43 ± 0.03, CI_Difference_ (95%) = [0.045, 0.226], *p* = 0.002, 32.4% increase, Supplementary Fig. S13B). Thus, image-level behavior was better predicted using task-trained compared to task-naïve IT recordings. Next, we tested how models with varying levels of IT-likeness can predict these differences in behavioral consistency. We found that there were indeed significantly different predictions according to a model’s IT-likeness (right panel, Fig. 5F, H(3) = 29.24, *p* < 0.00001), with IT-like models predicting the most accurate increases in IT-behavior consistency and IT-unlike models predicting no increase. In line with the object identity-invariant properties, this thus provides additional evidence that the evaluated IT-like models capture more general differences that extend beyond changes in the encoding of object-related information, reflecting both encoded image latents as well as fine-grained behavioral characteristics.

While IT-likeness was the strongest factor shaping these results, we also noticed that all IT-likeness groups featured several outlier models (Fig. 8F, left panel). An exploratory analysis revealed that such extreme values were exclusively linked to models trained using binary choice feedback but not using the other two training approaches (Supplementary Fig. S14A). Thus, while the IT-mapped layer changes did not distinguish between training strategies (cf. Fig. 6), at the behavioral level, binary-choice training produced qualitatively different image-by-image behavior despite comparable mean performance levels. A follow-up analysis further indicated that both standard and step-wise training produced highly similar image-by-image behavioral patterns. These two groups were also highly similar to the trained monkeys. In contrast, binary choice-trained models formed a distinct cluster (Supplementary Fig. S14B). Overall, this means that against our expectation, binary-choice training did not produce more monkey-like behavioral patterns, and instead, the other training strategies were better suited for obtaining models that resemble monkey behavior, on average, and at an image-by-image grain.

## Discussion

How does plasticity in the adult brain enable the learning of new objects? Taken together, our results indicate that object discrimination training induces modest but functionally meaningful changes in IT, while leaving a substantial portion of behavioral learning to be explained by downstream circuits. To understand how these changes in IT are connected to the behavioral improvements after training, we used task-optimized SMART (models of the ventral stream and object recognition behavior (see ref. ^1^) and show that both the direction and magnitude of these IT changes arise naturally from IT’s position within a hierarchical visual system adapting to support new object discrimination demands.

In the remainder of the Discussion, we use this systems-level framework to explain why IT plasticity is intermediate rather than dominant, to reconcile variability across prior IT learning studies, to account for training-induced changes beyond object identity, and to identify the representational constraints that distinguish IT-like from non–IT-like computational models.

### Adopting a systems-level perspective clarifies IT’s role in object learning

A key insight from our study is that object discrimination training changed IT responses to a moderate extent, yet the magnitude of these differences did not fully account for the behavioral improvements observed after training (Fig. 2M). This pattern of results rejected two initial hypotheses (Fig. 1F): (1) That IT does not change at all, since IT’s responses already held sufficient visual representations to support performance on the trained task (*H*_*0*_), and (2) that IT responses serve as a direct readout for behavior and, in turn, IT would reflect any changes apparent in overt behavior (*H*_*2*_). Our hypotheses can be loosely mapped to prior work on the effect of training on the encoding and readout of sensory inputs, typically focusing on early visual processing (e.g., ref. ^68^). In this view, *H*_*0*_ corresponds to a learning process that leaves the encoding unaltered and only achieves behavioral changes via changes to the down-stream readout (e.g., ref. ^69^). Conversely, *H*_*2*_ describes a process that emphasizes changes in the encoding that enable a linear readout for the trained behavior (e.g., ref. ^70,71^). Contextualizing IT’s difference with those in behavior enabled us to show that neither of these scenarios applied: IT’s responses were enriched in favor of the trained objects, indicative of a change to IT’s encoding, while at the same time, not differing to the same extent as behavior. These results support a middle ground, in line with *H*_*1*_: IT undergoes functional encoding changes, but most plasticity occurs downstream of IT.

Several of our observations suggest that the encoding changes in the IT cortex were functionally relevant for the animals’ trained behavior. First, training specifically increased linearly decodable information for trained objects compared to novel objects (Fig. 4), arguing against training-nonspecific, epiphenonemal changes. Second, trained IT responses became more consistent with the animals’ behavioral choices at an image-by-image level during passive viewing (Supplementary Fig. S13B), suggesting that the changes in IT could also shape behavioral decisions. Third, the slow, gradual nature of the behavioral learning curves (Supplementary Fig. S2)—which occurred despite subjects already being fully acquainted with the task aligns with theoretical accounts linking slow learning dynamics to changes in cortical encoding rather than rapid rule learning (e.g., ref. ^72^). This account predicts that the encoding changes we observed should be sensitive to differences in training curricula and show limited generalization to new scenarios^73^.

Our finding that IT undergoes moderate functional changes raises a natural question: why did IT encoding change to the degree that we observed and why not more or less? Prior IT training studies (for review, see ref. ^56^) span a wide range of stimulus statistics, task structures, and training demands, making it difficult to isolate which factors determine the magnitude of plasticity. For instance, stimulus complexity has ranged from simple, yet highly controlled feature configurations^38,39,41,45^ to complex objects^25,35,48^, morphed objects^31,42,51,74^ and complex patterns^43^, though nearly all presented stimuli on blank backgrounds at fixed positions. We here employed particularly complex stimuli — 3D objects rendered on varied scene backgrounds in random positions, rotations, and scales, adding more diversity to the existing stimulus type considered in prior studies. While our paradigm was a standard delayed-match-to-sample object discrimination task with a two alternative forced choice between the target and distractor, presented side-by-side on the choice screen (similar to ref. ^44^), many prior IT training studies adopted temporal versions of this paradigm requiring same/different judgments instead (e.g., refs. ^25,31,35,39^), or a go/no-go paradigm tied to category membership (e.g., ref. ^60^). Moreover, we deviated from prior studies by relying on a training paradigm that reinforced a large number of discriminations compared to a single binary discrimination as used in most prior studies (e.g., ref. ^31,41,60^). While it is conceivable that each of these design choices might affect the magnitude of learning-induced changes in IT, the existing diversity in prior findings makes it challenging to isolate which of these factors primarily determined the reported effect sizes. To address this challenge and understand what drives IT’s plasticity, we turned to computational modeling.

To better understand how such specific factors shape IT encoding, we developed a computational framework to simulate the plasticity needed at the level of the IT cortex to support the task performance shown by the trained monkeys. These simulations revealed that IT’s change magnitude across various metrics naturally followed from its functional role within the visual hierarchy. In other words, when we modeled IT as part of a larger network that gradually changes in a distributed fashion to optimize task performance, the models reproduced both the direction and magnitude of differences observed in IT responses. This perspective indicates that IT’s plasticity will depend on the training stimuli, the task demands (i.e., the similarity between objects, the number of discriminations), IT’s functional state before training, and a subject’s task performance after training. This view roughly aligns with proposals of perceptual learning in IT as part of a hierarchy of representations^57,75,76^, but critically we here turn this proposal into a testable hypothesis grounded in image-computable models and task performance.

Based on our simulations, we reason that differences in the visual features of the training stimuli, their linear decodability in IT before training, and an animal’s trained performance after training may have contributed to the diversity of effects sizes reported in prior studies (see ref. ^56^ for a review), with some studies reporting sizable changes with task training^25,31,41–44,77^ and others finding small or absent changes^35,38,39,78^. Accounting for these factors when comparing studies, combined with generating training-specific computational predictions, can help reveal the circumstances that drive or limit IT plasticity during task training in future studies.

At first glance, our findings of modest IT plasticity may seem at odds with studies reporting that human discrimination behavior is already fully accounted for by task-naïve IT decodes^19^ and that behavioral learning is well accounted for by leveraging general-purpose visual representations^40^. How can both be true? Our analyses revealed an increase in information efficiency in task-trained compared to task-naïve IT responses (Fig. 2L). This clarifies that task-trained responses do not fundamentally differ from task-naïve ones, yet subtle differences in IT’s encoding associated with task training might enable a more efficient downstream readout. Consequently, a sparser downstream readout would be sufficient to achieve the same behavioral performance. While decoding analyses like the ones used here are only a proof-of-principle that information is suitably formatted for downstream readout, it is also likely that such downstream populations do not have access to the entirety of IT responses or even the subset of populations we sampled for this study. Understanding the role of anatomical sparsity for these downstream projections is a promising direction for improving current computational models. Another critical question is whether training-induced changes in IT’s encoding are narrowly tuned to the specific distinction being reinforced (object identity) or whether they more broadly affect IT’s representational geometry. Our findings suggest the latter: training not only changed the linear separability of the trained objects but also affected other visual latents represented within the same neural populations, including spatial position and other identity-invariant features (Fig. 8). This pattern aligns with prior work showing that IT simultaneously represents multiple visual latents^67^ and recent evidence that training on different visual latents can yield convergent representations aligned with ventral-stream activity, partly because optimizing for one latent implicitly facilitates learning about others present in the world^79^. Critically, ANNs trained through distributed optimization across the network hierarchy naturally reproduced these multi-faceted changes, demonstrating that ANNs are an appropriate testbed for capturing IT-like encoding and its distributed changes during training. This perspective enriches prior views of perceptual learning by showing that changes are not limited to the trained dimension but rather reshape hierarchical representations that simultaneously encode multiple visual dimensions, making them amenable to a variety of downstream readouts.

At the core of the computational problem solved by the brain and ANNs during object learning is that of invariance, the ability to identify objects over a large range of viewing conditions^13^. In our study, successful performance required invariance to an object’s position, size, rotation, and, critically, its scene background. While invariance to intrinsic object properties is well-documented in the ventral stream^15,25,80–85^ and commonly encountered during natural viewing, our task imposed an additional demand: segmenting objects from highly variable backgrounds that, unlike natural viewing contexts (e.g., ref. ^86^), provided no informative cues about object identity. Prior computational work suggests that ANNs implicitly learn object-scene segmentation during object recognition training, even on natural images^87^, and that such segmentation strategies contribute to their ability to predict neural responses^88–90^. To what extent did this segmentation challenge contribute to the training-induced changes we observed? While our study was not designed with appropriate control conditions to directly test whether training improved general segmentation abilities, we performed a control analysis using novel objects (not part of the training set) displayed on backgrounds similar to those encountered during training. This analysis revealed no significant decoding benefit for these novel objects (Fig. 4), suggesting that training did not yield general improvements in background-invariant representations. However, this does not rule out object-specific segmentation processes that may have contributed to improved discrimination of the trained objects. Future studies with explicit segmentation controls could further clarify to what extent segmentation demands shaped the distributed learning processes we have identified.

### Beyond IT: The role of downstream networks in object learning

How does behavioral change emerge during object training, enabling primates to go from chance-level performance to higher accuracies? The modest changes we observed in IT, combined with evidence that object-relevant information is already present in naïve IT, suggest that learning may primarily involve optimizing how IT representations are mapped to task-relevant decisions. In our delayed-match-to-sample task, this mapping requires several computations: maintaining the sample image in working memory, comparing it with test stimuli, and translating the comparison into a motor response (left/right choice). These computations likely engage multiple brain regions beyond IT. At present, we lack task-computable SMART models for these downstream subnetworks. Notably, the IT-mapped layers in the evaluated base models were typically in the last third of the model (Supplementary Fig. S9) and not at the final layer. An exciting avenue for follow-up research could be to expand current models beyond the visual ventral stream. Indeed, previous work provides important clues about these downstream networks. The prefrontal cortex, particularly vlPFC, plays a crucial role in rule learning and working memory^30,91,92^. Moreover, direct manipulation of vlPFC activity causally impacts discrimination behavior and modulates IT responses^93^. The perirhinal cortex may help transform IT object representations into more categorical formats^29^. In addition, structures like the caudate nucleus show learning-related changes during category training^26^. A promising direction for future research is to study the impact of categorical vs. visual similarity on the magnitude of neural changes in and beyond IT. Such an analysis would clarify the extent to which our proposed framework generalizes to and captures plasticity induced across diverse task structures. Indeed, our observation that binary-choice training (learning about a single random distractor class on a given trial) resulted in markedly different image-by-image performance patterns compared to other training approaches (which processed feedback about all distractor classes, Supplementary Fig. S14) already highlights the importance of task structure for modeling primate behavior. Which computations are necessary for acquiring such task structure during the early phases of training is an exciting avenue for future work. In line with prior findings (e.g., ref. ^31^,), our modeling work also predicts that these downstream areas of IT should demonstrate higher degrees of plasticity (as compared to IT) due to their closer connection to generating trained task behavior. How distributed, task-specific, and engagement-dependent such downstream systems are in the brain is an important avenue for future research. Extending our SMART modeling framework^1^ to include downstream networks could help bridge this gap, generating concrete hypotheses about how different brain regions coordinate plasticity during learning.

### Understanding what makes models match IT Plasticity

Our analysis revealed that approximately 30% of all performance-matched models – that could perform the behavioral tasks with the same accuracy as trained monkeys – fully reproduced the training-induced differences observed in the macaque IT responses. This finding raises a crucial question: what determines whether a model will capture the hallmarks of object training in IT? Contrary to our initial hypotheses, we found that no single factor could reliably predict a model’s ability to match IT changes. While certain architectures and training strategies showed higher success rates (Supplementary Fig. S12), the relationship between model design and IT-like plasticity proved more complex than anticipated. For example, we included self-supervised pre-trained models in our set of base models, reasoning that a model’s prior exposure to categorical feedback during its pre-training may be an important determinant of how such a system responds to the introduction of object feedback^65^. However, we found no systematic advantage of self-supervised over supervised pre-training (Supplementary Fig. S12). These results challenge the assumption that specific, biologically motivated design choices will automatically improve a model’s alignment with the brain (or its changes with training). Instead, they highlight that IT-like plasticity emerges from complex interactions between a model’s initial state, architecture, and learning dynamics.

If modelers’ intuitions about the right design choice offer no direct guidance, how can we progress in developing useful computational models of the brain to motivate future experiments? Our findings underscore the critical importance of empirical benchmarks in this computational modeling effort^94^. By requiring models to simultaneously match behavioral performance and neural differences, we established stringent criteria for evaluating different computational hypotheses. This approach revealed that IT-like object learning systems can arise from various combinations of factors, with successful models sharing similar functional properties despite architectural differences. The predictive power of these models extended beyond our initial benchmarks to novel phenomena (Fig. 8), validating their capture of fundamental principles underlying IT plasticity. However, important questions remain about the apparent diversity of successful models. Do they truly implement equivalent solutions to the object learning problem, or are our current benchmarks insufficient to distinguish between different computational strategies? We started to tackle this question by performing a geometric manifold analysis, asking whether there are any properties that set apart IT-like models from less IT-like ones. Interestingly, we found that models that maintained the size of their object manifolds over training were the most like IT. This finding is in accordance with another study on perceptual learning that also identified reductions in manifold size as the key driver for improving information during learning^66^. This speaks to the importance of coordinated, subtle and distributed changes within biological systems and learning principles that prioritize invariance within classes over a separation between classes. While, to our knowledge, this study is among the largest investigations of such effects in non-human primates, future work with larger samples and more targeted model-separation-driven measurements may help discriminate between competing model classes. Such research could further constrain the space of viable computational theories and potentially reveal previously unrecognized principles of neural plasticity.

### Gradient descent as a window into neural plasticity

Our success in modeling IT plasticity using gradient descent raises important questions about the relationship between artificial and biological learning. While gradient descent may not mirror biological learning mechanisms, our results suggest it captures fundamental principles about how hierarchical networks optimize behavior. Our network simulations suggest that a model of IT’s plasticity is readily obtained by combining SMART models with task optimization via gradient descent to mimic object training. We here viewed gradient descent as an effective tool to simulate a normative perspective on the observed neural changes. In other words, if one wants to understand how a distributed system *should be* adapted in service of task performance, gradient descent provides the optimal solution to achieve this refs. ^3,4^. From this perspective, the success of our modeling approach may not be surprising. Viewing our results through this lens, we found that IT plasticity appears to follow principles of efficient task optimization, where changes in sensory representations cannot be understood in isolation from behavioral demands. The magnitude and distribution of observed plasticity reflect both computational constraints and network architecture. However, the relationship between gradient-based learning in artificial networks and plasticity in biological circuits requires careful consideration. There remains a possibility that the changes achieved via gradient descent, even though they are optimal, are not achievable in biological brains. While gradient descent computes optimal weight updates using non-local information propagated backward through the network, biological circuits face more severe constraints^6,7^. Neural plasticity likely operates with more constrained mechanisms than artificial networks, such as predominantly local information at synapses^95^, temporally sparse and delayed feedback signals^10^, and circuit motifs that may restrict information flow^6,9^. For example, the structure of neural circuits could limit how task-optimizing error updates may be approximated and propagated throughout the system, affecting the distribution and magnitude of changes in IT. Remarkably, our results suggest these biological constraints may not critically impact the pattern of plasticity at the scale of IT responses and behavior. We found that multiple learning algorithms, operating under different constraints, could produce changes matching empirical observations. This implies that the observed IT plasticity may reflect general principles of hierarchical learning rather than specific implementation details. Nevertheless, it is conceivable that these factors will become important when modeling inter-areal coordination, learning dynamics over time, individual differences in learning trajectories, and the transfer of learning to novel tasks. Future work combining our normative modeling approach with more biologically detailed implementations may help bridge the gap between artificial and biological learning mechanisms.

### Limitations and future directions

While our study provides insights into how object learning shapes IT responses, several important limitations suggest promising directions for future research. A central limitation is our reliance on between-subject comparisons rather than tracking learning-induced changes within individual subjects. Though a within-subject design would be ideal for understanding how object learning modifies neural circuits, two key challenges justified our between-subject approach. First, characterizing learning-induced plasticity within individuals requires precise measurement of baseline states and careful separation of true plasticity from measurement variability. Current techniques for longitudinal neural recordings face significant challenges in maintaining stable measurements across extended periods, making it difficult to definitively attribute response changes to learning rather than recording instability or sampling differences^96,97^. While recent advances in chronic recording methods and statistical frameworks for analyzing individual variability^98^ show promise, the field still lacks robust approaches for tracking fine-grained neural changes over learning timescales. Second, our between-subject design enabled the collection of a larger dataset (six subjects) with better statistical power than would be possible in a within-subject study given current technological constraints. This approach revealed consistent training-induced changes across subjects, providing strong evidence for systematic plasticity in IT during object learning shared across individuals. Nevertheless, future studies combining improved longitudinal recording methods with our computational framework could reveal important insights about how individual learning trajectories change in tandem with IT, which our current between-subject design could not address.

Beyond tracking individual changes, understanding object learning will require examining how multiple brain regions coordinate plasticity. A key strength of our approach was leveraging the well-established quantitative link between IT responses and object recognition behavior^19,99^. This link provided a crucial advantage: we could systematically evaluate plasticity by measuring how well neural changes predicted behavioral improvements. Unlike many studies of neural plasticity where the relationship between neural changes and behavior remains unclear, our understanding of IT’s role in visual recognition allowed us to test specific hypotheses about how learning modifies neural circuits in the service of behavior. Future work should leverage similar brain-behavior relationships to examine how regions like the prefrontal cortex^93^ and perirhinal cortex^29^ interact with IT during different kinds of learning (e.g., at the level of objects and categories). Our modeling framework could help generate specific predictions about plasticity in these regions, guiding experimental design and data analysis. Moreover, a promising avenue for refining our understanding of how the observed differences in IT are implemented is to refine the spatial and functional scale of the mapping between IT recordings and ANN activations, for example, by mapping out correspondences to excitatory and inhibitory neurons (e.g., ref. ^100^).

Another crucial direction is understanding how learning unfolds across different timescales and its interplay with passive exposure via unsupervised learning^101–105^ as well as with pre-existing perceptual expertise^106–108^. Our study examined the end state of object training, but important questions remain about how representations evolve during learning. How do unsupervised learning, passive exposure and existing perceptual expertise facilitate the observed difference in IT responses? How do early and late stages of learning differ? What is the interplay with other nearby structures, such as the hippocampus in different stages of training? It also remains an open question of how dependent the effects described here were on the presentation duration of the stimulus and our chosen analysis window: Indeed, prior work suggests even more pronounced effects during later windows, evoked by longer image presentations^109,110^. Answering these questions will require new experimental paradigms that can track neural changes at multiple temporal scales while maintaining measurement stability, as well as computational models that can express these different hypotheses.

## Methods

### Subjects

The non-human subjects in this study were 7 male rhesus monkeys (Macaca mulatta; 5–10 years of age). Since this study was primarily concerned with sensory processing, the subject’s sex was not considered in the study design. We grouped these monkeys as trained (monkeys M, N, and B) and naïve (monkeys C, T, and S) with respect to a set of object discrimination tasks. We collected data from an additional task-naïve monkey P for an additional control analysis (Population-level decoding of untrained objects, Fig. 4).

We could perform a rigorous comparison across such a large cohort since all included monkeys also participated in other studies (data and methods from Monkey C and T have been described in ref. ^19^; those from Monkey N, M, and S in ref. ^111^; Monkey N and B in ref. ^93^; and Monkey M and N in ref. ^99^). All surgical and animal procedures were performed in accordance with National Institutes of Health guidelines and the Massachusetts Institute of Technology Committee on Animal Care.

### Naturalistic object stimuli

We generated synthetic, yet naturalistic object images using free ray-tracing software (http://www.povray.org, see Fig. 1D for examples. Each image was rendered from a 3D object model (purchased from TurboSquid) into a 2D projection while varying an object’s position (horizontal and vertical position), rotation (in the horizontal, vertical, or depth plane), and size (Supplementary Fi.g S1A). We then added the rendered object view to a randomly chosen natural image background (indoor and outdoor scenes obtained from Dosch Design, www.doschdesign.com). All resulting images were gray-scaled and had a resolution of 256 × 256 pixels.

The image dataset contained 8 possible objects (bear, elephant, face, apple, car, dog, chair, plane, Fig. 1A). The training image dataset consisted of 100 images per object (800 images in total), whereas the test image dataset consisted of 80 images per object (640 images in total). Training image parameters were sampled from a wider range than for the test images (see Supplementary Fig. S1B for the sampled distributions). Training images were thus more varied than test images in their position, rotational properties and size.

Finally, the canonical views for each of the 3D objects were defined in prior work^67^ to align with the common views of that object (Fig. 1A). In brief, animals were shown facing forward, with their head upright. The car was shown with the car grille forward and its tires on the bottom. The chair has its legs facing downward while the seat is facing forward. The person is looking straight at the viewer with the top of the head oriented upward. The apple was shown with its stem on top, from an arbitrarily picked rotation (due to its rotational symmetry around the vertical axis). The plane’s cockpit faced, in an upright position.

As part of a control analysis (Fig. 4), we developed a second image dataset containing 10 additional objects (pineapple, necklace, trout, doctor, mirror, trousers, pen, fork, pumpkin, clock) with 50 generated images each using the same procedure as for the main dataset. The generation process for these images was identical to the main image set.

### Monkey training and behavioral tasks

#### Fixation training and the passive viewing task

We trained all monkeys to maintain fixation on a central fixation dot using operant conditioning with liquid rewards. Specifically, monkeys received rewards for fixating within a square fixation window (2 or 2.5°). We monitored eye movements using video-based eye tracking (SR Research EyeLink II and 1000).

Monkeys initiated a trial of the passive fixation task by looking at a fixation dot. After initiating fixation (300 ms), we presented a sequence of 5–10 object images, each shown for 100 ms at the center of gaze (8°) and followed by a gray blank screen of 100 ms (see Fig. 2A for a schematic). If the subject maintained fixation inside the trained fixation window for the entire duration of the image sequence, the image sequence was followed by a fluid reward and an inter-trial interval of 500 ms. All neural data was collected during this passive viewing task to create similar viewing conditions for both groups.

#### Object training and active binary object discrimination task

The object-trained group was additionally trained to perform a binary (match-to-sample) object discrimination task in their home cages using a touch screen (Fig. 1B). The full procedure is described in refs. ^112,113^ and details on the code and hardware are available at https://github.com/dicarlolab/mkturk and ref. ^114^. In brief, subjects initiated a trial by touching a central white circle centered on the bottom third of the screen. This triggered the presentation of a sample image for 100 ms presented at the center of the screen (subtending approx. 6–8°). A choice screen immediately followed the sample image. The choice screen contained two object images in their canonical view (the same canonical images were presented across all trials, Fig. 1A). One of these objects (Target) matched the object in the sample image. The other (Distractor) was chosen randomly from the seven remaining objects. Monkeys were allowed to respond immediately by touching anywhere within the pixel boundaries of the choice object images. Selecting the correct image resulted in the delivery of a fluid reward, whereas an incorrect choice resulted in a 3 s timeout. The training phase contained 100 rendered images per object (*N* = 800 images). At the start of object training, subjects were already familiar with the match-to-sample task structure from prior training using different stimulus sets, but were naïve to the specific object identities used in the present study. Thus, while the animals had extensive experience with trial initiation, sample presentation, two-alternative choice screens, and reward contingencies, they had no prior exposure to the trained objects (cf. Supplementary Fig. S2C). Subjects gradually improved their performance over the course of the training (Supplementary Fig. S2A), which likely reflected object-specific learning rather than acquisition of the task rules themselves, and they transitioned to the testing phase once their training performance saturated (for details on this criterion, see ref. ^113^). This criterion was reached after 23/9 sessions (across 44/12 days) with a total number of 27034/17808 trials for monkey N and M, respectively. Training data for monkey B was not recorded. During the testing phase (i.e., *active binary object discrimination task*), monkeys saw novel images of the trained objects (*N* = 640 images, 80 per category) in a head-fixed setup using their fixations to make their choices instead of their hands. Specifically, behavior was tested while monkeys viewed images on a 24-inch LCD monitor (1920 × 1080 at 60 Hz, positioned 42.5 cm in front of the animal). To initiate a trial, monkeys fixated a white dot (0.2°) for 300 ms. A sample image was then presented centrally for 100 ms (8°), followed by a blank screen for 100 ms and the choice screen. During the choice period, monkeys could freely move their eyes (for up to 1500 ms). Monkeys made a choice by fixating on the selected image for at least 400 ms. If the monkeys selected the target object, they were rewarded with a fluid reward. A trial was aborted if a subject broke fixation any time before the choice screen (i.e., if a subject’s gaze was further away than 2° from the central fixation dot).

### Large-scale multi-electrode recordings in the inferior temporal cortex

#### Surgical implants and chronic micro-electrode arrays

All monkeys were surgically implanted with a head-post under aseptic conditions and with up to three 10 × 10 microelectrode arrays (Utah arrays, Blackrock Microsystems) per hemisphere in IT in a separate surgery. Each array contained 96 electrodes (excluding corner electrodes), each 1.5 mm long and with a 400 μm spacing between electrodes. The placement of arrays was guided by the visible sulcus pattern during surgery (for the locations per subject and hemisphere, see Supplementary Fig. S3 for the initial group of monkeys and Supplementary Fig. S8 for monkey P). For subjects with recordings from both hemispheres, arrays were first implanted and recorded in one hemisphere, and after approximately a year, these arrays were explanted, and new arrays were implanted in the other hemisphere.

#### Electrophysiological recordings

All neural activity was filtered using a 54-order finite-impulse-response band-pass filter (300-6000 Hz), yielding a 55-tap filter that attenuates frequencies outside the passband. Most data contained multi-unit activity. Spike events were detected after data collection. In particular, a spike event (multi-unit) was detected when the voltage (falling edge) exceeded more than three standard deviations of the filtered raw voltage values. Firing rates in response to a specific image were obtained by averaging the peristimulus time histogram spike counts (10 ms bins) between 70 and 170 ms after image onset. We chose this time window, building on prior work showing that responses in this window feature a linear link with choice behavior^19^ and that these responses can be reliably predicted by ANNs (e.g., ref. ^115^).

In both groups, arrays sampled a variety of regions along the posterior-to-anterior IT axis (Supplementary Fig. S3). Rather than focusing on potential anatomical differences, we here focused on the inferior-temporal cortex as a whole, treating each site as a random sample from the overall IT population. This choice was informed both by prior studies linking IT responses to object recognition behaviors (e.g., ref. ^19^), as well by statistical considerations. In particular, we reasoned that combining all measurements across IT would give us the highest signal-to-noise ratio, an aspect we deemed particularly important given the reported variability of effect sizes in the literature^56^.

During recording sessions, we monitored the monkeys’ eye movements using video-based eye tracking. At the start of a session, monkeys performed an eye-tracking calibration task by making a saccade to spatial targets and maintaining fixation for 500–700 ms. We repeated this procedure if a drift in the eye-tracking signal was noticed during a session.

#### Recording timelines

In the trained group, neural data were collected across 41, 57, and 10 recording sessions for Monkeys N, M, and B, respectively. In each session, monkeys viewed a set of 1320 unique images, with images repeated multiple times, resulting in approximately 2000–8000 total image presentations per recording day. Recordings in trained animals began after completion of object training, with neural data collection starting approximately 8 months (Monkey N) and 6 months (Monkeys M and B) after training.

In the task-naïve group, recordings were collected across multiple days rather than structured training sessions. Monkey S contributed data from 3 recording days with 640 unique images per session, while Monkeys C and T contributed data from 65 and 68 recording days, respectively, with 2560 unique images per session. As in the trained group, images were repeated within sessions, yielding approximately 2000–8000 image presentations per day.

### Neural and behavioral data analyses

#### Behavioral metrics

We quantify behavioral performance as the image-level discriminability reflecting how well a given object can be distinguished from all other possible objects in a given image (similar to refs. ^93,99^ and defined in ref. ^112^). Specifically, discriminability for an image *i* is defined as:

$${d^{\prime} }_{i}=z({HR}(i))-z({FAR}(i)),$$

where the hit rate (HR) is the proportion of trials for which an image *i* was correctly classified as object *c*. The false alarm rate (FAR) for object *c* refers to the average proportion of trials in which any image of another object was incorrectly assigned to object *c*. Finally, *z* refers to the inverse of the cumulative Gaussian distribution.

To estimate the reliability of this image-level metric for the monkey’s behavioral data, we compute the Spearman-Brown-corrected split-half reliability across 20 repetitions for random splits of trial repetitions per image.

#### Neural recording quality metrics and pooling

We aimed to assess group-level differences between task-trained and -naïve recordings at the population level. To ensure that potential differences did not stem from other factors, such as a difference in the number of sites in every group or reliability, we analyzed the recording sites in pseudo-random pools drawn as bootstraps from all recording sites (within a given group). We thereby assume that any recording site is a sample from the underlying population response and test the hypothesis that this population is changed due to task training. For every draw, we first resampled all recording sites (with replacement) for all subjects. Then, we constructed a single pool using these resampled sites for every group (naïve or trained). To construct the pools, we sampled an equal number of sites per subject (without replacement) as well as sites with an evoked split-half reliability response of at least 0.3 (mean across 1000 repetitions, spearman-brown corrected). This procedure could still produce group differences in reliability since the reliability distributions differ across datasets (Supplementary Fig. S4A). To create IT pools comparable in reliability, we, therefore, sampled recording sites using a weighting based on the untrained reliability distribution (estimated using a Gaussian kernel density estimate; see Supplementary Fig. S4B). We repeated this sampling procedure 1000 times, resulting in 1000 neuron pools for each group with comparable reliability (median: − 0.002, see Supplementary Fig. S4C). Within these pools, neural changes were quantified on the repetition- and trial-averaged firing rates of the first 36 image repetitions. Additional control analyses demonstrate that the choice of reliability criterion did not qualitatively affect our main results (Supplementary Fig. S5).

#### Site-level object selectivity

To investigate whether the examined neural recordings differed in how strongly any site modulated its response to the trained objects, we computed an object selectivity index for every recording site (similar to ref. ^31^). For a given site *u* in a pool, we computed the pairwise absolute differences for every pair of images. Next, we averaged the absolute pairwise distances between images from the same object $({S{O}_{u}})$ and those of different objects $({D{O}_{u}})$. To obtain an index of object selectivity ${S}_{u}$, we finally compared the difference between those two averages and normalized this difference by their sum:

$${S}_{u}=\frac{D{O}_{u}-S{O}_{u}}{D{O}_{u}+S{O}_{u}}$$

To compare task-trained and -naïve distributions, we summarized all selectivity indices using the median within a pool, thus providing 1000 estimates of selectivity in every group.

#### Population-level object representational similarity

We adopted representational similarity analysis (RSA^61^) to assess whether the relative representation of a given object is altered by training. Computing the neural representational distances and comparing them to an ideal object-level model provides a useful additional perspective to linear decoding techniques since it enables us to inspect whether images with the same object show an increased tendency to cluster beyond their overall separability (from other objects).

We first computed the pairwise distances of responses across all sites and images using the Euclidean distance to estimate object-level representational similarity. This yielded a symmetric representational dissimilarity matrix across images for every pool. In a second step, we computed the second-order correlation of the lower triangle (excluding the diagonal) with an object-level RDM (assigning full dissimilarity to images of different objects and zero dissimilarity to those of the same object). Since the object-level RDM is ordinal, we adopted Kendall’s tau for the second step with a correction for rank ties to avoid inflated estimates^116^.

#### Population-level object decoding

To estimate how much information can be linearly decoded from IT neural populations by downstream neurons receiving inputs from IT, we used linear classifiers, which have previously been shown to sufficiently link IT population responses to human behavior^19^.

We optimized linear classifiers (one-vs-all) on the image-evoked time-averaged rates in a given pool to predict the objects in the shown images. Specifically, we fit logistic regression classifiers with L2-regularization using a 3-fold cross-validation procedure with stratified random splits (across objects). This procedure provided held-out object predictions for all images. The inputs to the regression models were z-scored based on the training data split. We repeated this procedure 20 times (for different data splits) and computed image-level *d’* based on all predictions (see *Behavioral metrics)*. We adopted the mean performance (image-level *d’*) across repetitions in further analyses. Regression models were implemented in scikit-learn^117^ adopting default parameters.

#### Population-level decoding of object-orthogonal properties

In addition to object-related information, we assessed group-level differences in the available information about object-orthogonal properties (object location, eccentricity, size, and rotation, similar to ref. ^67^). Note that the properties were also used during the image generation process, and therefore, ground-truth values were available for all properties.

To estimate information about object-invariant properties, we followed the same approach as for object decoding with two key differences: To accommodate the prediction of continuous properties, we used Ridge Regression (implemented in scikit-learn^117^ and adopting default parameters), and performance was quantified as the Pearson correlation between the held-out predictions (cross-validation) and the ground-truth image properties.

#### Population-level object decoding of untrained objects

To understand whether differences in IT were specific to the trained objects, we created an additional image dataset with 10 novel, untrained objects (50 images each, Fig. 4A). This dataset was not seen by the initial task-naïve monkey cohort. We therefore recorded from a new task-naïve subject (monkey P) for this specific analysis. We analyzed the neural recordings in exactly the same fashion as for the other subjects (55 sites passed the 0.3 reliability criterion for this subject) and adopted the same analysis as for the other population-level object decoding analyses.

#### IT-to-Behavior consistency

We probed the alignment between monkey image-level performance (behavior, *b*) and IT decodes (neural, *n*) by estimating the image-level consistency between behavior and neural responses. Specifically, we computed the Pearson correlation between the image-level behavioral and IT object *d’* and then normalized this correlation by the geometric mean of their spearman-brown-corrected split-half reliabilities:

$${\rho }_{{consistency}}(b,n)=\frac{\rho (n,b)}{\sqrt{\rho ({n}_{1},{n}_{2})\rho ({b}_{1},{b}_{2})}}$$

We repeated this procedure 20 times with different split halves of trials (i.e., repetitions of an image) to estimate the reliability of this measure.

### Simulation of monkey training using artificial neural networks

We built a variety of simulations of the monkey’s training to ask whether any changes observed empirically between trained and naïve IT responses are consistent with updates to current computational models of the visual ventral stream.

#### Base models

As our base models of task-naïve monkey IT and behavior, we considered image-computable artificial neural networks optimized on large-scale image databases previously shown to be predictive of IT responses and discrimination behavior (e.g., Brain-Score platform^118^,). To reflect that the pre-training of these models might influence their fit to the empirical data, we included models optimized with supervised training on ImageNet (ResNet18, 34, 50, 101, 152, VGG16, 19, and AlexNet), weakly-supervised pre-training followed by supervised training on ImageNet (resnext101_32x8d_wsl, resnext101_32x16d_wsl) and self-supervised training on ImageNet (ResNet50 trained with MoCov2, barlowTwins and SimCLR). All supervised and weakly-supervised base models were retrieved from Pytorch^119^, whereas self-supervised models were obtained from VISSL^120^.

#### Aligning base models to task-naïve IT responses

To ensure that potential changes observed between task-naïve and -trained IT responses are comparable in scale and feature complexity in the considered base models, we first aligned the candidate models (before training) with the untrained IT sites. To achieve this, we identified the layer in all considered base models with the highest encoding capabilities of IT responses using standard procedures in the brain-score framework. Specifically, we computed a cross-validated PLS regression between a layer’s activation features and IT responses, providing a predictivity score for all evaluated layers. In all analyses, we simply picked a base model’s layer with the highest score as our IT-mapped layer (see Supplementary Fig. S9 for an base model overview, incl. the layer scores).

It is an open question how a single unit in an ANN model layer relates to a single recording site in a monkey’s IT cortex. Previous work on linking ANNs to neural responses has adopted various approaches (e.g., adopting all layer units and performing dimensionality reduction, e.g., ref. ^121^,). Here, we attempted to mimic the sampling process of the neural recordings as closely as possible, rather than assuming access to the information of all layer units. To this end, we assessed at what scale (units per recording site) the decodable information from randomly drawn units in a base model’s IT-mapped layer is comparable to the average decoding accuracy observed in task-naïve IT recording sites (on average across 300 pools, 159 sites per pool).

To achieve this, we first sampled object decodes (see *Population decode accuracies*) for different scaling factors in the IT-mapped layer using a log-scale sampling (1–100 unit scaling, equivalent to 159–15900 randomly sampled units). In a second step, we then fit these data points using an x-shifted log function, where *a*, *b*, and *c* were free parameters:

$$\hat{d^{\prime} }={a} \, {log}({unitScaling}+b)+c$$

Fitting this function to model simulations using different scaling factors gave us a continuous function between the number of randomly sampled units in the IT-mapped layer and its decodable information (*d’*, see Supplementary Fig. S10A). Finally, using this fitted function per IT-mapped base model layer, we identified the scaling factor equivalent to the task-naïve IT recording decodes (Supplementary Fig. S10B). If a base model’s IT-mapped layer failed to reach this criterion within the sampled range, we excluded that base model from further analysis. We found that none of the weakly-supervised IT-mapped layers (resnext101-32x8d-wsl and −32x16d-wsl) reached performance levels on par with task-naïve IT decodes. As a result, we excluded these base models from the training simulations.

#### Training strategies

How monkey training compares to training an artificial neural network is an open scientific question that touches on many current issues, including what mechanisms support different forms of plasticity in the brain or whether back-propagation is a proxy of biological learning. In this work, we sought to make this comparison tractable by focusing our efforts on currently available training approaches capable of yielding performance improvements in object classification for the training images seen by the trained monkeys.

We matched the monkey and model training parameters wherever possible and included a wide range of parameters when the analogy was less clear. To mimic the monkey’s training, we only included images the monkeys have seen during training (*N* = 800). Furthermore, we optimized any models for 15 epochs, equivalent to an exposure of 1500 repetitions per object, previously shown to be a typical timescale for monkeys to learn new objects^113^. For any choices where we could not draw an analogy to the monkey training, such as the learning rate, batch size, regularization, and the optimizer’s momentum, we set up a search space with parameter ranges informed by common choices in machine learning, reasoning that this would produce a variety of performant models with diverse parameter configurations. We adopted a Bayesian hyperparameter search implemented via the weights and biases framework for 20 repetitions^122^.

Finally, we adopted a few standard practices for optimizing neural networks: we optimized all models using a cross-entropy loss function, a stochastic gradient descent optimizer, and a step decay learning rate scheduler (reducing the learning rate every six epochs by a factor of 0.1). We dedicated 30% of the training images as a validation set. This enabled us to choose the best model across training epochs for further evaluation. Beyond these choices (common to all trained models), we devised three training strategies:

*Standard finetuning*. We replaced the output layer of a pre-trained model with a randomly initialized fully connected layer. During training, we provided 1-hot feedback across all eight training classes. The resulting error gradients could modify any weight in the pre-trained network.

*Step-wise finetuning*: This approach was identical to standard finetuning, with the only difference being that we initialized the fully connected layer with an optimized linear regression readout. This approach yields higher-performing finetuning results and improved generalization by circumventing large errors early in training^123^. This training choice was motivated by the observation that common training paradigms typically span multiple days and thus may feature both within and between-session improvements^56^.

*Binary choice:* This strategy mimicked the feedback structure in a delayed match-to-sample task with two choices used for monkey training. In contrast to the feedback provided during supervised training specifying both the target as well as the absence of all distractors (1-hot encoding, information on all seven distractors), monkeys only received feedback on one target and one distractor class in a given trial. To simulate this form of sparser feedback, we modified the gradients such that a model only received feedback on the target and one randomly chosen distractor class for a given image.

All considered candidate models were optimized 20 times for every training strategy, resulting in 660 trained models (11 models, 3 strategies, 20 repetitions).

#### Model selection

We selected trained models for a detailed comparison with the empirical data if the trained model reached a behavioral performance level (average *d’* across images) on par with the trained monkeys (i.e., within the 95% confidence interval of the hierarchically pooled monkey behavior).

#### Quantifying changes in candidate models

To assess the changes in the IT-mapped layers, we adopted the same three metrics used during neural data analysis (i.e., selectivity, linear decoding, and representational similarity). All metrics were computed for 100 randomly drawn unit pools. For site-level selectivity, we adopted the average response of drawn units (at the specified site-to-unit scaling) as a proxy of site-level selectivity.

#### Comparing model and neural differences

To effectively compare neural differences to those of the candidate models, we first computed the relative difference for all metrics (*ϕ*) in both models and the neural data:

$$\%{{\Delta }}_{{\phi }}=\left(\frac{{{\phi }}_{{trained}}}{{{\phi }}_{{na}\ddot{\iota}{ve}}}-1\right) * 100$$

This estimates the difference (Δ*%*) for every pair of draws (from the task-trained and -naïve pools, respectively) for the neural data and the IT-mapped layers. We averaged across the repeated draws (from the IT-mapped layer) to obtain a model’s (*Δ%*) estimate. We considered models as IT-like if the (*Δ%*) estimate across all metrics fell within the 95% confidence interval of the observed changes in the IT data (Fig. 6C). In turn, models that only matched a subset of metrics were less IT-like, and models that matched none of the metrics were referred to as IT-unlike.

#### Quantifying learning-induced geometric changes in IT-mapped layers

To characterize how learning altered activations in the IT-mapped layers, we adopted metrics proposed by Cheng et al.^66^, quantifying four geometric principles that may enhance task information within population responses. For each object, we computed the difference vector between target and non-target class centroids (Δf) and the pooled covariance matrix (Σ) across both classes, separately for task-naïve and task-trained model activations. Signal Enhancement (SE) was measured as the ratio of trained to naïve difference vector magnitudes:

$${SE}=\frac{{|{\Delta } \, f}_{{trained}}|}{{|{\Delta } \, f}_{{na\ddot{\iota}ve}}|}$$

Manifold shrinkage (MS) was quantified as the ratio of the median response variances, reflecting changes in the spread of within-class response distributions:

$${MS}=\frac{{median}({diag}({\varSigma }_{{trained}}))}{{median}({diag}({\varSigma }_{{na}\ddot{\iota}{ve}}))}$$

Signal Rotation (SR) was computed as the angle θ between naïve and trained difference vectors, calculated from their dot product after unit normalization, with angles wrapped to range [0°, 90°]:

$${\theta }=arccos \left(\frac{{{\Delta } \, f}_{{trained}}\cdot {{\Delta } \, f}_{{na}\ddot{\iota}{ve}}}{{|{\Delta } \, f}_{{tra}i{ned}}|\times {|{\Delta } \, f}_{{na}\ddot{\iota}{ve}}|}\right)$$

Manifold Warping (MW) was assessed by performing eigendecomposition on both covariance matrices and computing the angles between the corresponding eigenvector *i* pairs, again wrapping angles to [0°, 90°]:

$${\theta }_{i}=\arccos ({\varepsilon }_{{{\rm{i}}},{tra}{{\rm{i}}}{ned}}\cdot {\varepsilon }_{{{\rm{i}}},{{na}\ddot{\iota}{ve}}})$$

We reported MW separately for each principal component to capture how the covariance structure reorganization varied across dimensions. Units with mean activations below 0.001 in either condition were excluded prior to all analyses to ensure numerical stability. Per model, we report the averaged metrics across all objects.

### Statistical analyses

To test for differences between task-trained and -naïve IT pools, we adopt two increasingly conservative approaches: First, to test for differences between groups (1000 draws sampled pools with replacement), we performed a two-sample bootstrap test by computing the differences in the bootstrapped distributions and derive a *p*-value from the probability of zero under that difference distribution (i.e., we construct the confidence interval of the difference distribution and check whether it contains zero). We report the absolute and relative difference of our standardized metrics (e.g., selectivity or *d*’) as a measure of effect size. Second, to account for different sources of within and between-subject variability during our neural pooling procedure (Supplementary Fig. S7), we also performed a hierarchical permutation test comparing differences observed in the empirical data with those expected from pools sampled with permuted group assignment (similar to a hierarchical randomization approach by ref. ^124^). To implement this, we created all possible combinations of groups (while ignoring the experimental conditions) and performed our standard neuron pooling procedure for these groups. This gives a null distribution of differences for the randomly created groups, which we then adopt to interpret our empirically observed differences (i.e., the difference between task-naïve vs. trained pools). Due to the computational demand of this second approach, we reduced the number of draws for any group to 200.

To compare model differences to those in the empirical data, we tested whether the average estimated differences of a model fell within the 95% confidence interval of the empirical data (across draws/bootstraps). This approach was applied to both the behavioral and IT-based metrics.

To test for the effect of IT-likeness on a model’s similarity in change, we perform a non-parametric one-way analysis of variance by ranks (Kruskal-Wallis test, *H*). Effect sizes are reported as ε² = (*H* - k + 1) / (n - k) with k referring to the number of conditions and n to the overall sample size.

We did not make assumptions of normality in our analyses and adopted an alpha level of 0.05 to evaluate statistical significance throughout the project.

### Software

All analyses were performed in Python 3.10. ANN models were implemented and trained using PyTorch (v1.12.1^119^) with torchvision (v0.13.1) and CUDA (v12.4). Analyses relied on Numpy (v1.26.4^125^), Pandas (v2.2.3^126^), SciPy (v1.14.1^127^), scikit-learn (v1.5.2^117^), and Pingouin (v0.5.5^128^) for statistical tests. Data visualization was performed using matplotlib (v3.10.0^129^) and seaborn (v0.13.2^130^). Hyperparameter optimization and model training were tracked using Weights & Biases (wandb; v0.19.2^122^).

### Reporting summary

Further information on research design is available in the Nature Portfolio Reporting Summary linked to this article.

## Supplementary information

Supplementary Information

Reporting Summary

Transparent Peer Review file

## Source data

Source Data

## Data Availability

All data has been deposited in the following Open Science Framework repository (https://osf.io/dcgze/). Source data are provided in this paper.

## Code availability

All code can be accessed on the Open Science Framework repository (https://osf.io/dcgze/), on GitHub (https://github.com/lynnsoerensen/ObjectTraining_IT_ANNs) and on Zenodo (10.5281/zenodo.19955396).
