# Supplementary material for: Hierarchical optimization predicts plasticity in the macaque inferior temporal cortex following object training
Source: Nat Commun. 2026 Jul 8;17:8434. doi: 10.1038/s41467-026-74816-0 (PMC13478300; doi:10.1038/s41467-026-74816-0)
Supplement: Supplementary file 1 [file 41467_2026_74816_MOESM1_ESM.pdf]

Supplementary figures

Hierarchical Optimization predicts Plasticity in the Macaque Inferior Temporal Cortex following Object Training

Authors

Lynn K. A. Sörensen, James J. DiCarlo, and Kohitij Kar

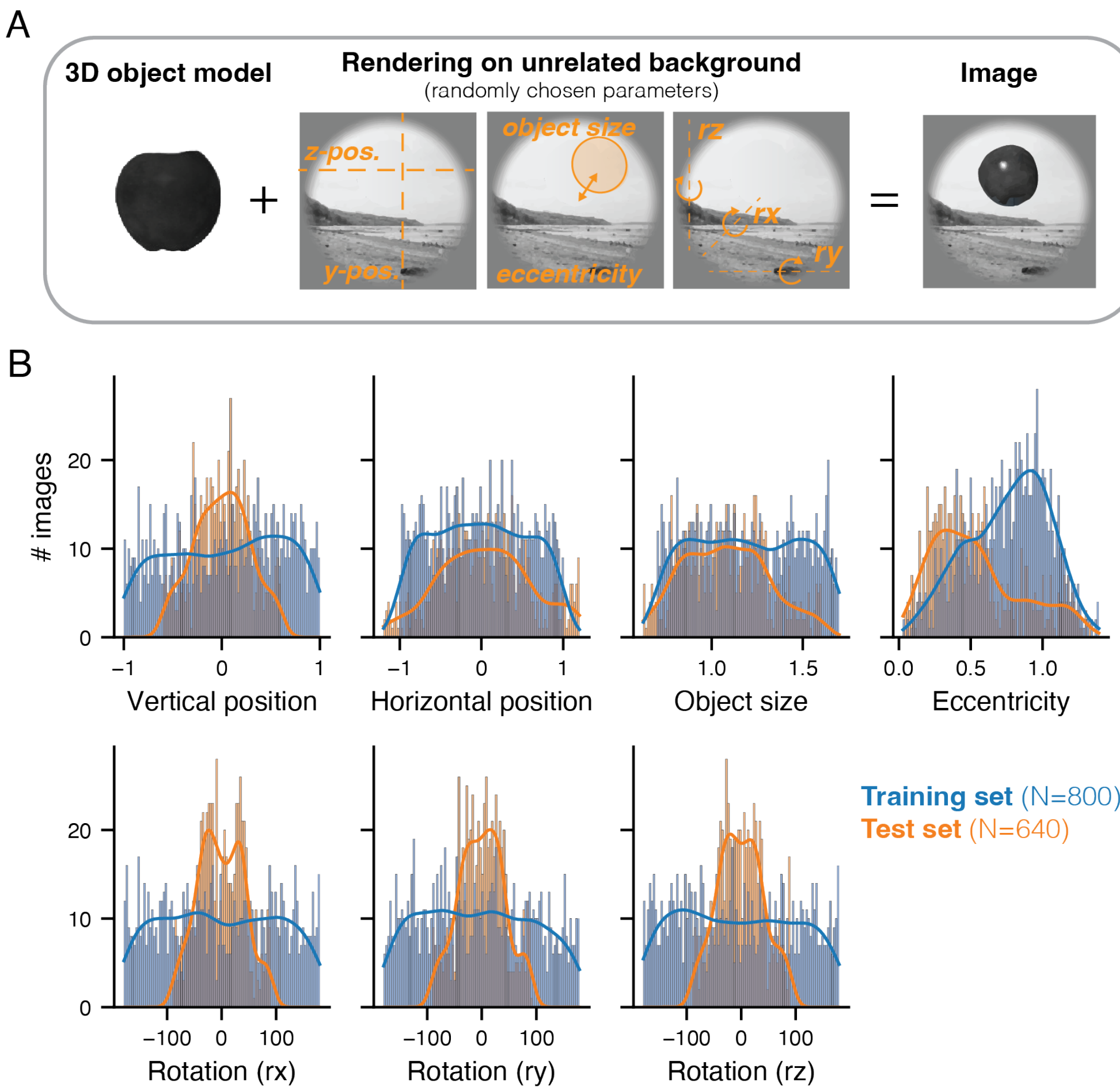

Figure S1. Image dataset generation. *(A) Training and test image datasets were generated by rendering a 3D object model on a randomly chosen scene background. The 3D object model was rendered using a randomly chosen set of parameters (within a specified range), yielding a novel object image. (B) Comparison of the training and test image sets across the rendering parameters. Test images were sampled from a narrower parameter range compared to training images. Every image (incl. training and test sest) was generated from a unique combination of parameters and there was no link between an object's identity and its rendering parameters.*

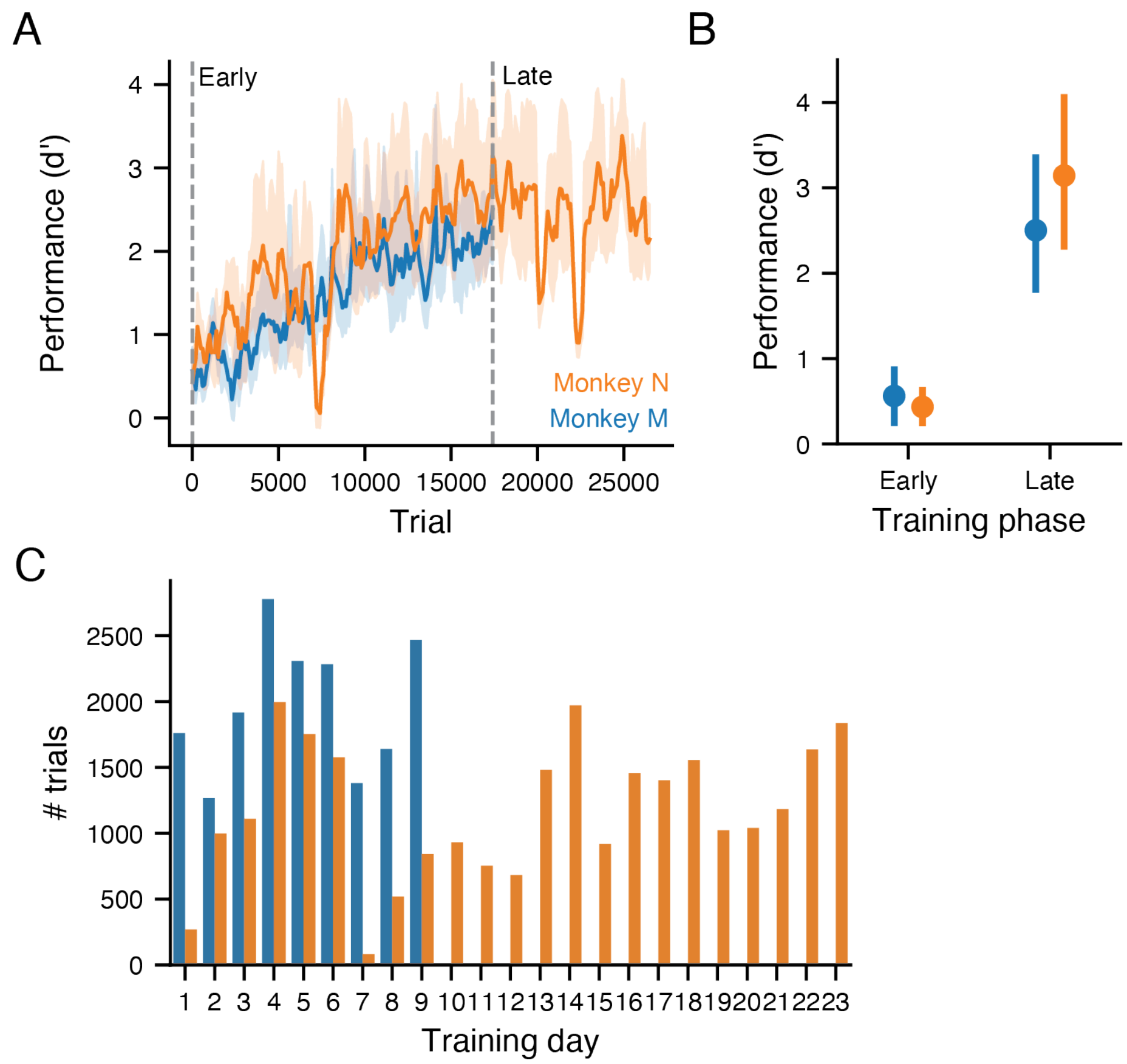

Figure S2. Object discrimination performance improves gradually during training. *(A) Learning curves for task-trained monkeys. Performance is estimated using a sliding window of 500 trials for each object; the x-axis indicates the starting trial of each window. Shaded areas show 95% confidence intervals of the mean bootstrapped across the eight objects. Dashed vertical lines mark the early and late training periods analyzed in panel B. (B) Comparison of early versus late training performance. Monkeys showed near-chance performance early in training but robust discrimination late in training (trial window: 17400-17900 for both subjects), demonstrating a substantial improvement over the training period. (C) Number of completed trials per session for each subject. Monkeys completed hundreds of trials per session from the start of training, demonstrating familiarity with the task structure despite initial unfamiliarity with the specific training objects (corresponding to low early performance in panel A).*

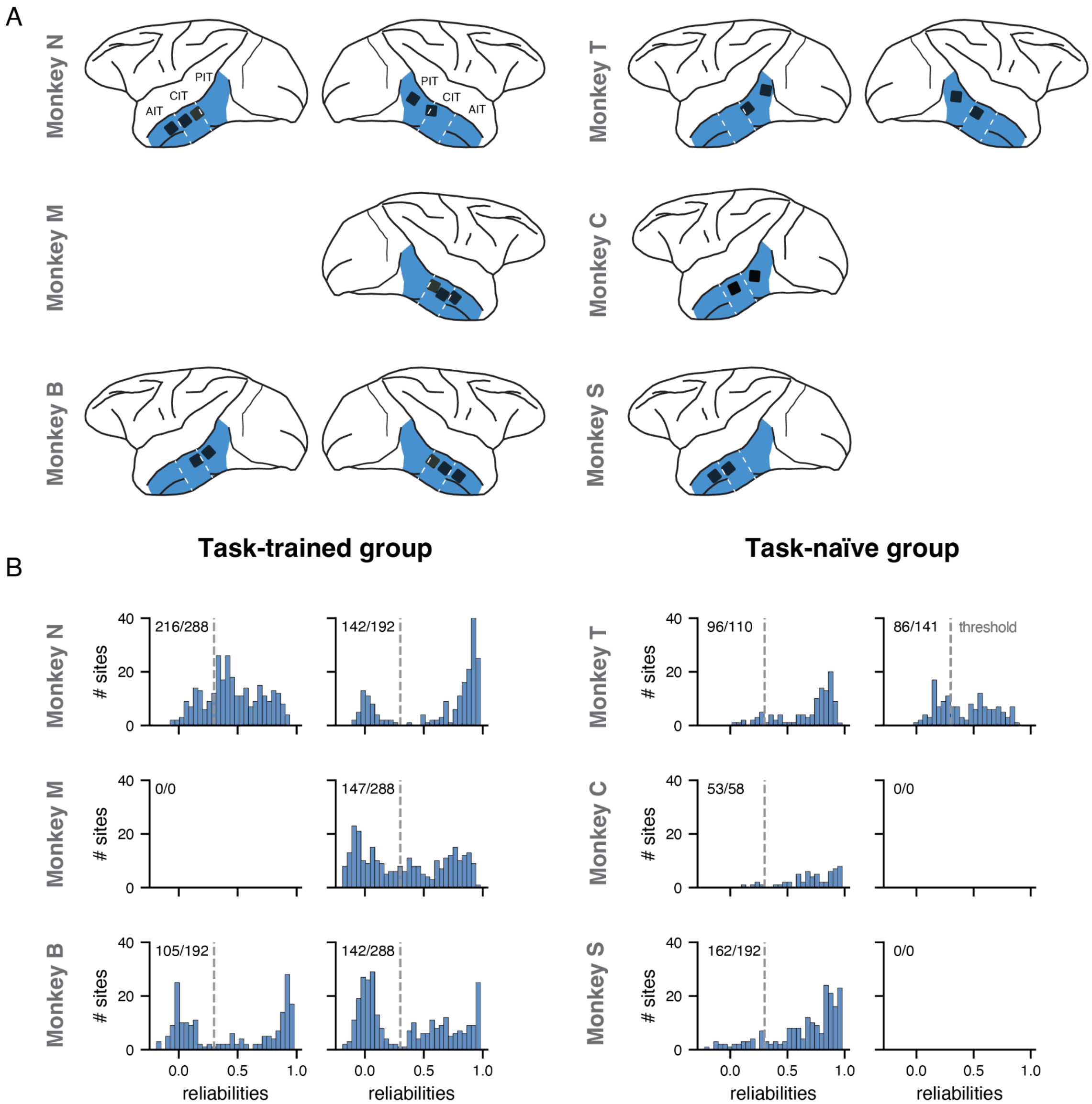

Figure S3. Micro-electrode array placements & electrode signal-to-noise ratio. *(A) All subjects had chronically implanted Utah arrays in various locations along the posterior-to-anterior axis in the inferior temporal cortex. Note that for subjects with bilateral arrays shown, recordings were performed consecutively in both hemispheres (see Methods for further details). Arrays were classified as AIT, CIT or PIT according to their position relative to the anterior and posterior middle temporal sulci (annotated by the grey dashed lines). For our main analysis, recording sites were pooled across IT subregions, treating all recording sites as random samples from an average IT population. (B) Signal-to-noise ratio of all recording sites per hemisphere and subject was computed as the split-half reliability across repetitions (see Methods for further details). The dashed line indicates the reliability criterion of 0.3 for site inclusion adopted in the main analysis. Each panel is annotated with the effective yield (i.e., sites that passed the reliability criterion) and the total number of available recording sites. The panel layout follows that of (A).*

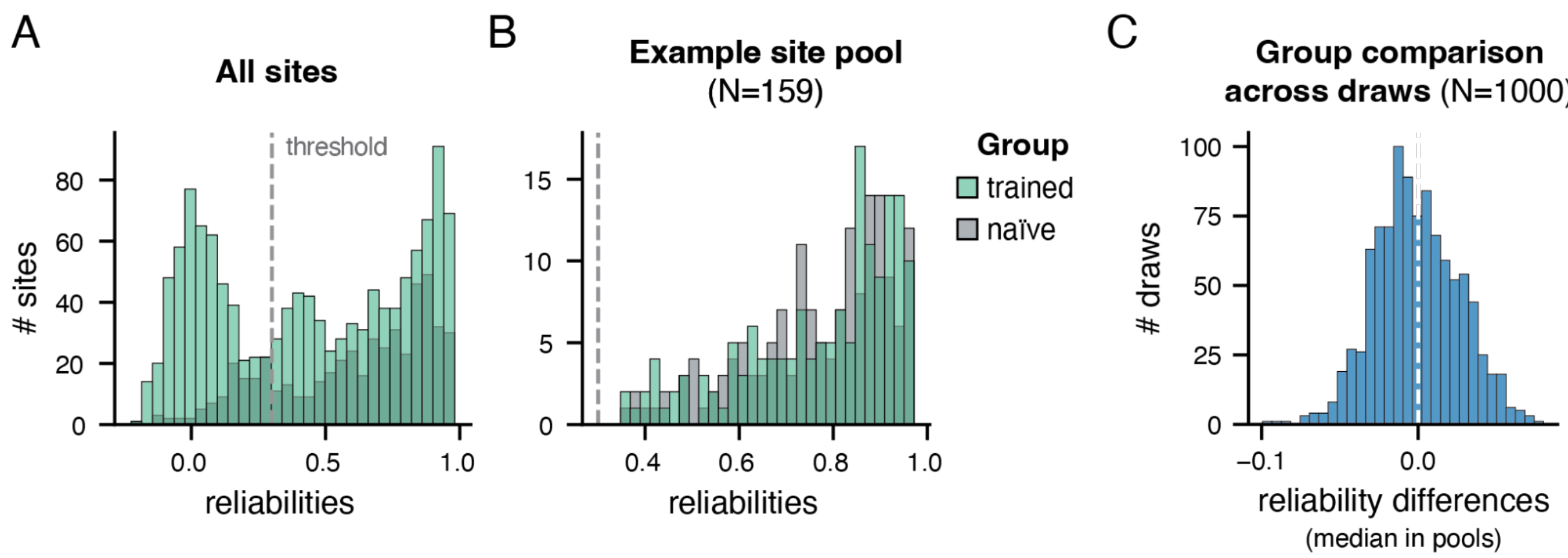

Figure S4. Matching site reliability across groups. *(A) The IT recordings collected from task-trained and -naïve subjects differed in the number of available sites and the reliability distribution across sites. We computed reliability as the correlation across averages from random split-halves of trial repetitions (N = 1000). The dashed line shows the applied reliability threshold of 0.3. (B) Our pseudo-random pooling procedure (see Methods) produces pools of recording sites corrected for reliability and number of sites per subject (53 sites/subject, thus 159 sites/pool). (C) Using this procedure, we obtain pools that are comparable in their reliability. A total of 1000 pseudo-random pools are shown resulting in a median of -0.002 difference in reliability between groups across all draws.*

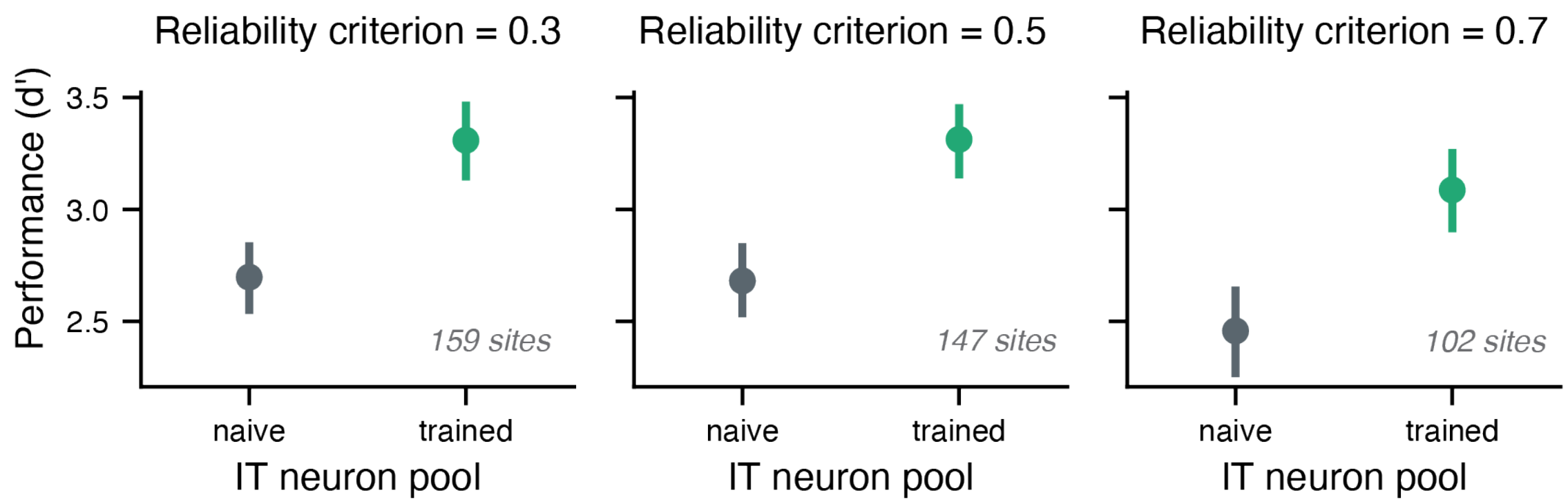

Figure S5. Training-induced decoding differences are not dependent on the choice of the reliability criterion. *Neuron pools were sampled from recording sites passing a given reliability criterion. To test whether our results depend on this criterion choice, we repeated the analysis using different reliability thresholds. Although increasingly stringent reliability criteria (left to right) progressively reduced the number of available sites (annotated in grey), training-induced differences in decoding accuracy remained significant across all thresholds (all $p < 0.001$, two-tailed two-sample bootstrap test). We show the mean and its 95% confidence interval across draws.*

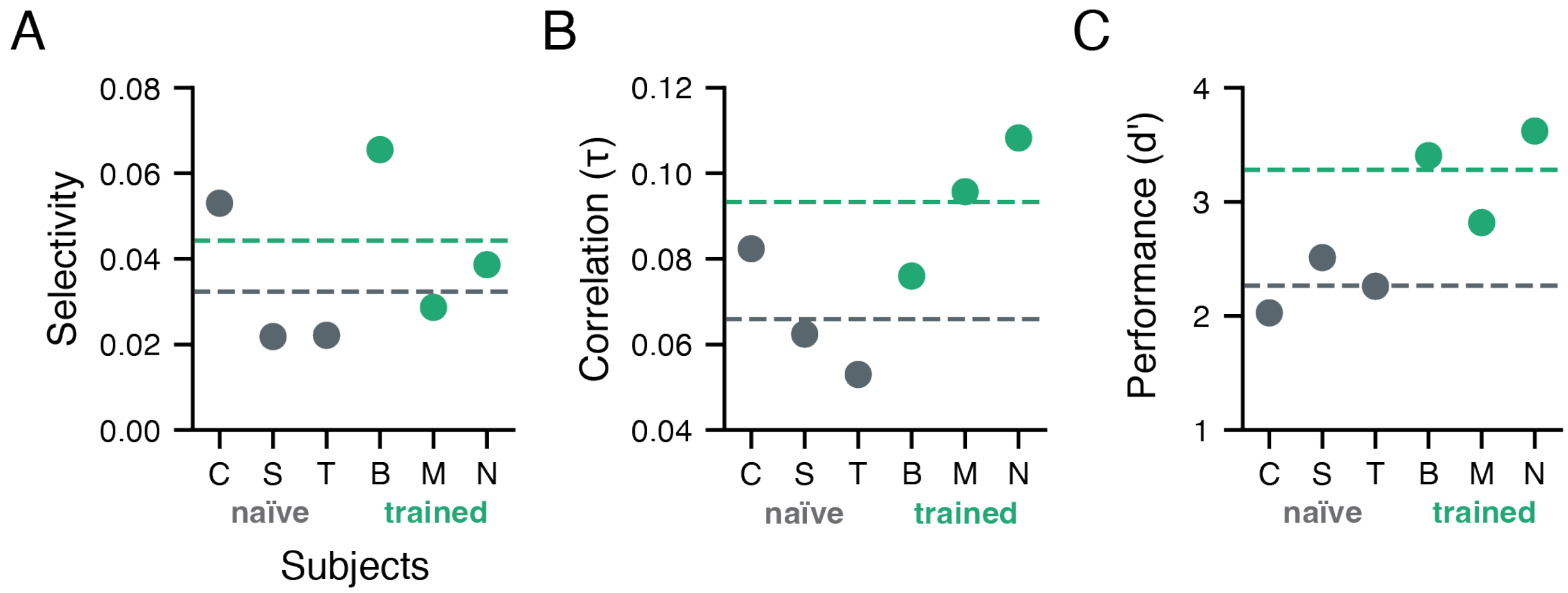

Figure S6. Single-subject level analyses. *To determine whether training effects are observable at the individual subject level, we computed our key metrics (A) site-level selectivity, (B) representational strength and (C) linear decodability using the full dataset available for each subject. We included only sites with split-half reliability higher than 0.3 but did not subsample or match for reliability differences between subjects. Gray and green dots show individual subject estimates for task-naïve and task-trained animals, respectively. Dashed lines show the mean across the three subjects in each condition. Task-trained subjects tend to show higher values than naïve subjects across all metrics, indicating robust training effects at the individual level.*

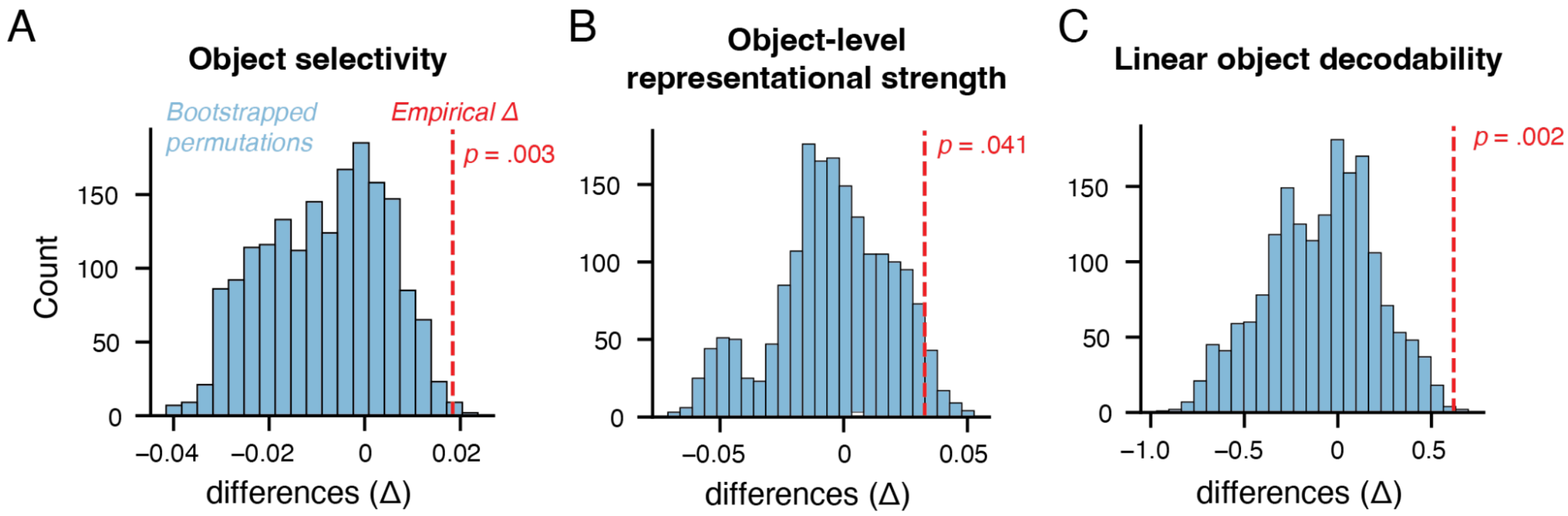

Figure S7. Hierarchical permutation bootstrap test. *To what extent are the observed differences driven by differences between a few subjects irrespective of their training history? To control for the effects introduced by interindividual variation in both groups, we sampled a bootstrapped null distribution (200 draws) while permuting the group membership of all monkeys (null distribution shown in blue) for all metrics (A-C). Relating the observed differences (empirical, red dashed line) to this null distribution revealed that all observed effects were larger than those expected from interindividual variation.*

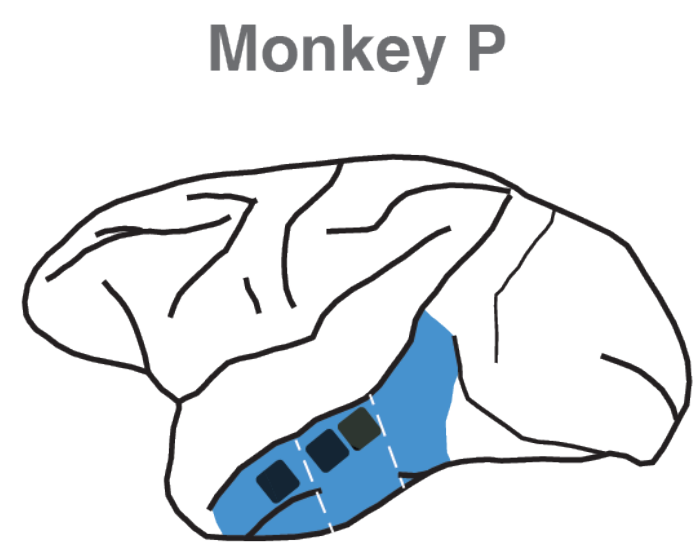

Figure S8. Micro-electrode array placements for Monkey P. *Data from Monkey P were recorded for an additional control analysis presented in Fig 4.*

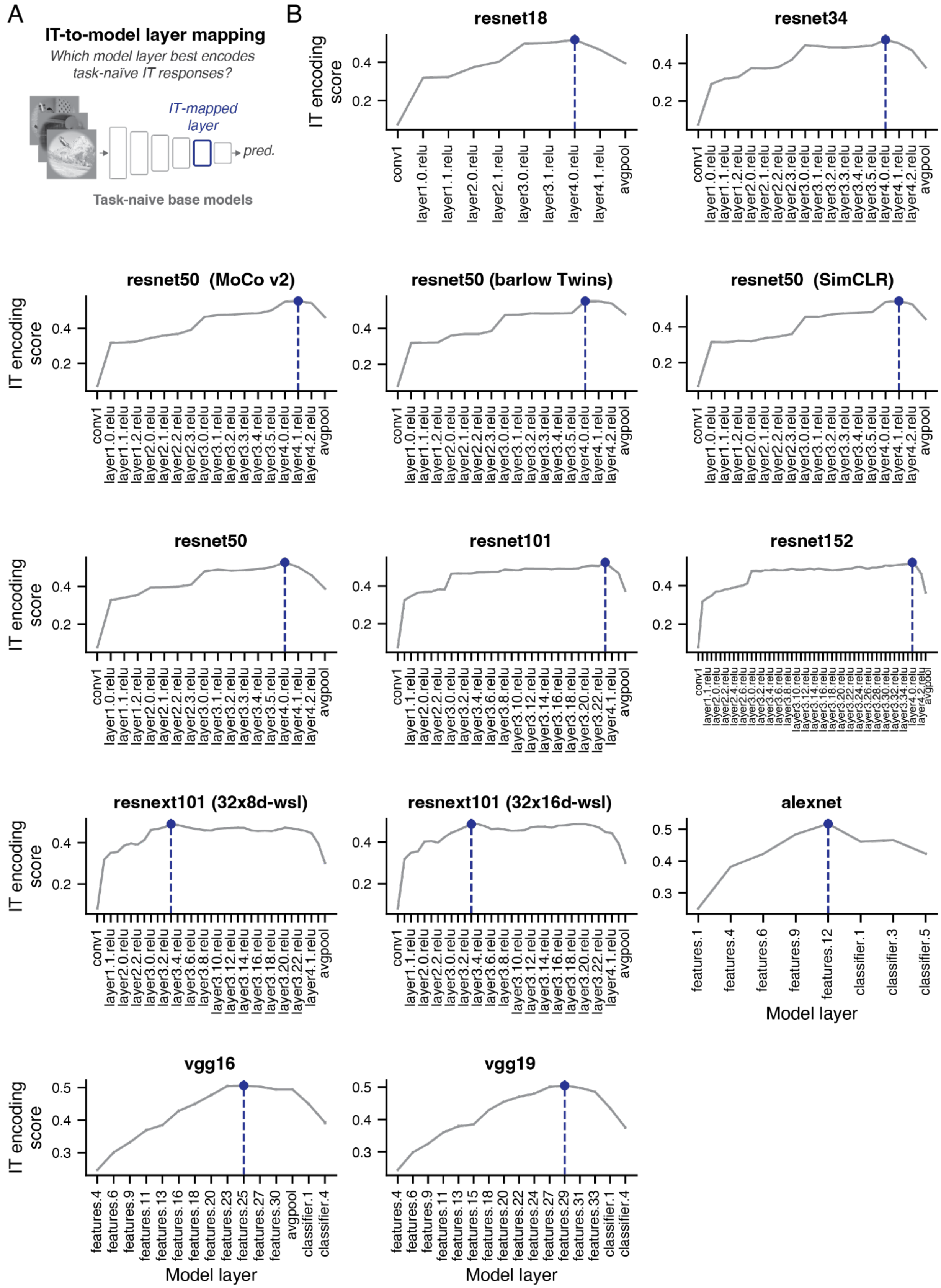

Figure S9. IT-layer mapping for base models. *(A) We selected the layer for comparison with the IT data*

by asking which layer in a given base model best encodes the responses in naïve IT recordings. (B) We evaluated this by adopting the approach taken by the Brain-Score platform. In particular, for any given layer, we fit a cross-validated partial least squares regression model to predict neural IT responses. We repeated this approach ten times, each yielding an IT encoding score for a given set of layer features. Here we show the mean IT encoding score across repetitions as a function of layer position within the model (x-axis). The final chosen IT-layer is annotated with the vertical dashed blue line. For visualization purposes, we reduced the number of annotated x-axis labels for all resnet101 and 152 models.

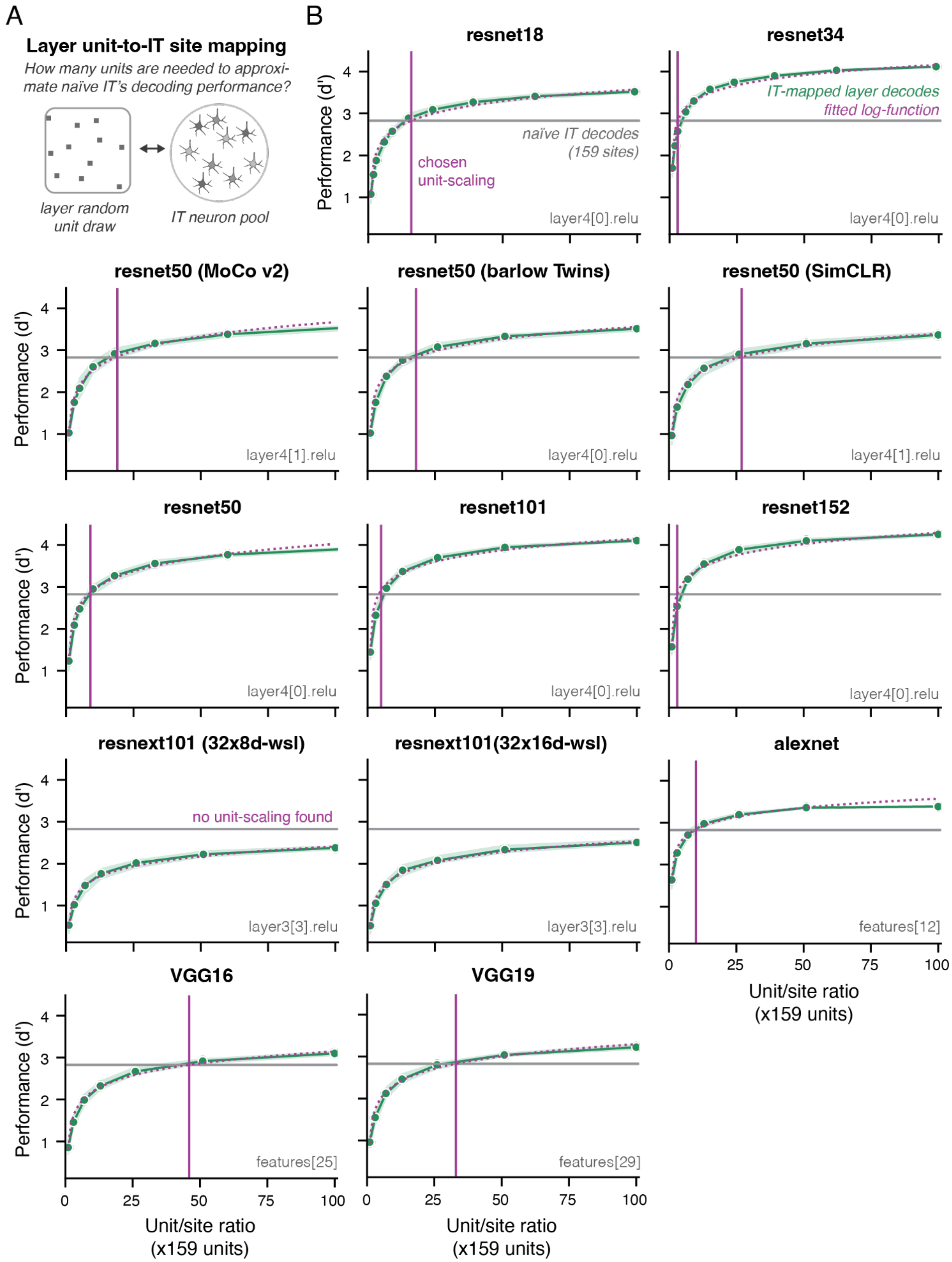
A
Layer unit-to-IT site mapping
How many units are needed to approximate naïve IT's decoding performance?
layer random unit draw
IT neuron pool
B
resnet18
resnet34
Performance (d')
chosen unit-scaling
naïve IT decodes (159 sites)
IT-mapped layer decodes
fitted log-function
layer4[0].relu
resnet50 (MoCo v2)
resnet50 (barlow Twins)
resnet50 (SimCLR)
layer4[1].relu
resnet50
resnet101
resnet152
resnext101 (32x8d-wsl)
resnext101(32x16d-wsl)
alexnet
no unit-scaling found
layer3[3].relu
features[12]
VGG16
VGG19
features[25]
features[29]
Unit/site ratio (x159 units)

Figure S10. Site-unit scaling for base models. *(A) For the chosen IT-mapped layer (cf. Fig S9), we assess how many layer units are necessary to approximate the average information in an IT recording site. (B) Decoding performance increases as a function of the number of randomly sampled units in an IT-mapped layer for all base models (one subplot each). Performance was estimated using 20 random draws and the error bars show the 95% confidence interval across draws. The x-axis refers to the unit-to-recording site ratio. A ratio of 1 means that 159 units have been randomly sampled. The gray line shows the average decoding performance obtained from task-naïve IT pools (cf. Fig 2K). We fit the neural decoding performance with a log function (shown as a dotted line in purple) and then find the intersection of that curve with the untrained performance of IT responses. We adopt the unit-site ratio at this intersection (purple vertical line) as the chosen unit-site scaling for a given base model.*

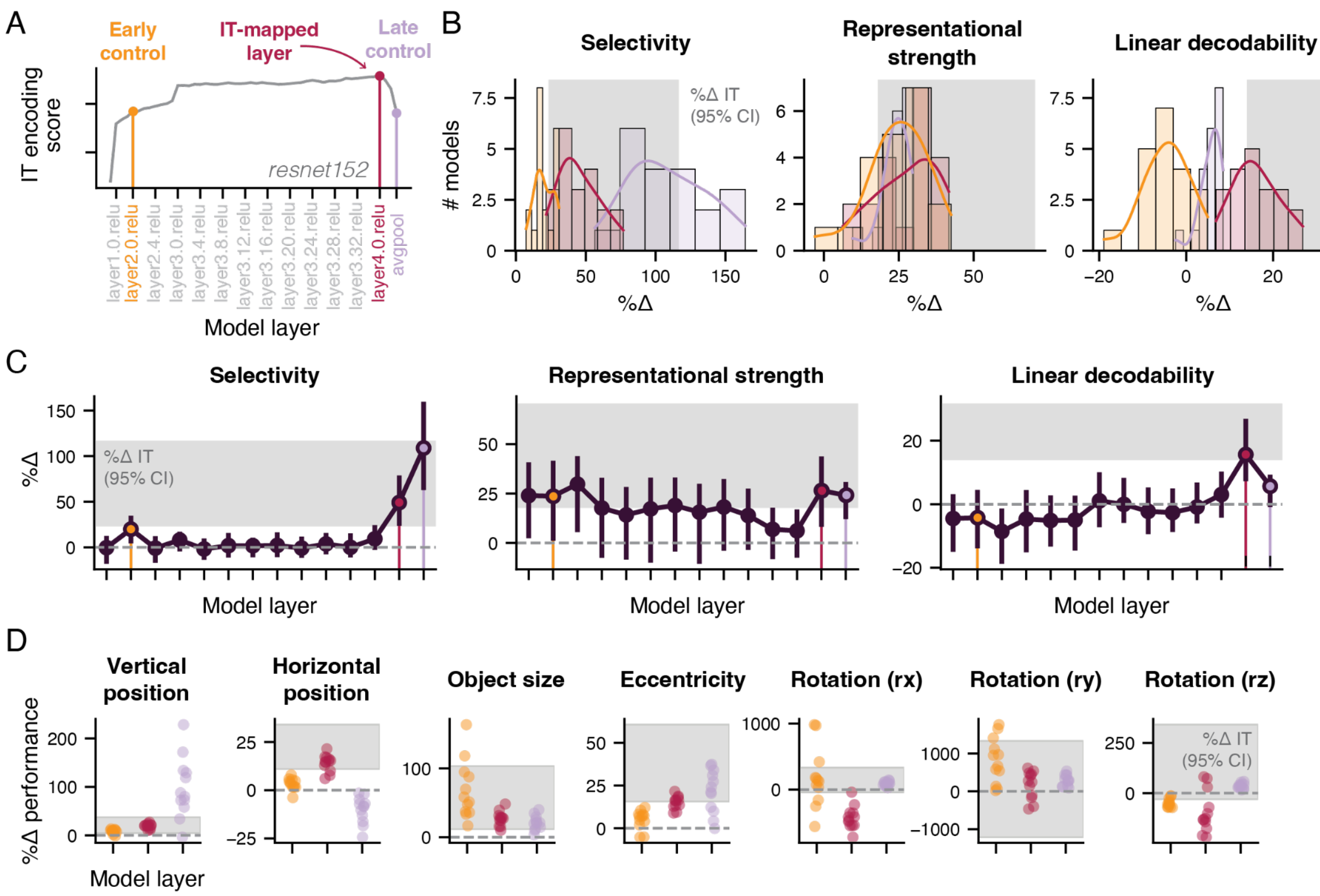

Figure S11. IT-like layers best capture neural IT differences. *(A) For our main analysis, we selected the model layer showing the best encoding of naïve IT responses (i.e., IT-mapped layer, red line). To validate this approach, we compared this IT-mapped layer to control layers for one base model (resnet152). All evaluated layers are annotated on the x-axis. For two control layers, we performed more extensive analyses: (1) a late control layer, the next deeper layer after the IT-mapped layer, which showed the worst IT encoding score (lilac line), and (2) an early control layer, an earlier layer matched to the late control in IT encoding score (orange line). All comparisons used the same 20 trained model instances of resnet152. (B) Whereas model changes from the IT-mapped layer (red histograms) fell mostly within the observed IT changes, model changes from the more extensively evaluated control layers (orange and lilac histograms) tended to fall outside of the empirically observed differences. (C) Examining layers across the entire model reveals that the IT-mapped layer best matches the neural data for the selectivity and decoding metric. For representational strength, earlier and later layers fit the neural data on par with the IT-mapped layer. The x-axis shows the annotated layers in (A). We plot the mean across 20 models and show the 95% confidence interval. (D) Is the ability to generalize to untrained dimensions specific to the IT-mapped layer? To assess this, we focused on models that fit all three IT metrics (N = 12) and adopted the early and late control layers for predicting object-invariant training differences (cf. Fig 8). We find that for most properties, the IT-mapped layer tends to better predict the observed IT differences compared to the control layers.*

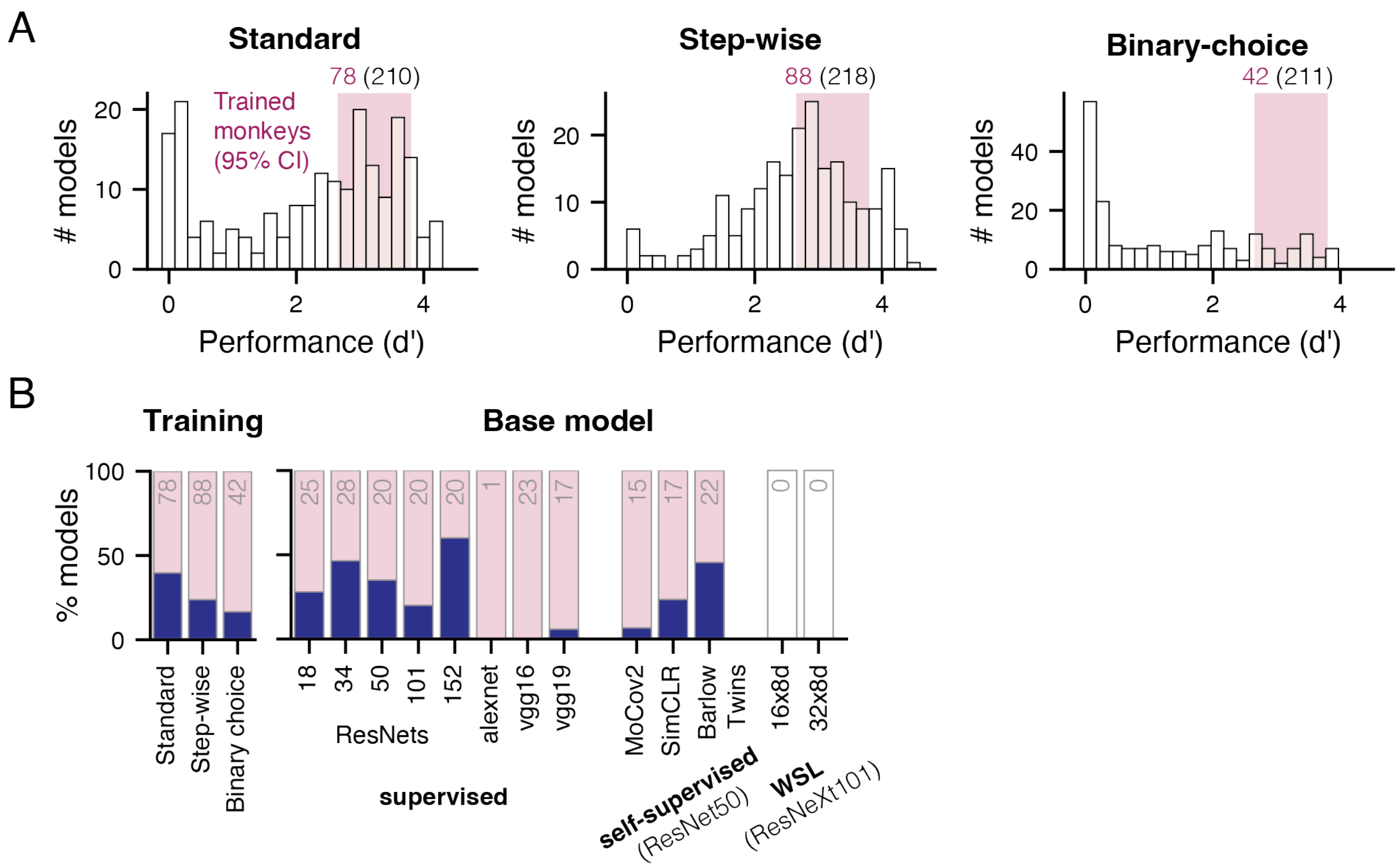

Figure S12. Model inclusion as a function of training parameters. *(A) We only considered models as hypotheses of IT plasticity that matched the performance of the trained monkeys (magenta shaded areas depict the 95% CI of the pooled monkeys' behavior). Binary choice training produced the fewest equally performant models, followed by standard and stepwise training. Each training consisted of 20 model iterations (with different combinations of hyperparameters) for the 11 base models for which we could establish an IT-layer mapping. A few model trainings failed, thus producing fewer than 220 models. (B) Which training aspects were associated with producing IT-like models? Every training strategy and most base models could generate models that matched IT along all metrics. The empty bar and annotated numbers represented the number of models that passed behavioral screening (A) and sufficient decoding (cf. Fig S10) to establish a mapping with IT responses. The filled blue bar shows the proportion of models that match all three metrics (i.e., selectivity, representational strength, and linear decodability).*

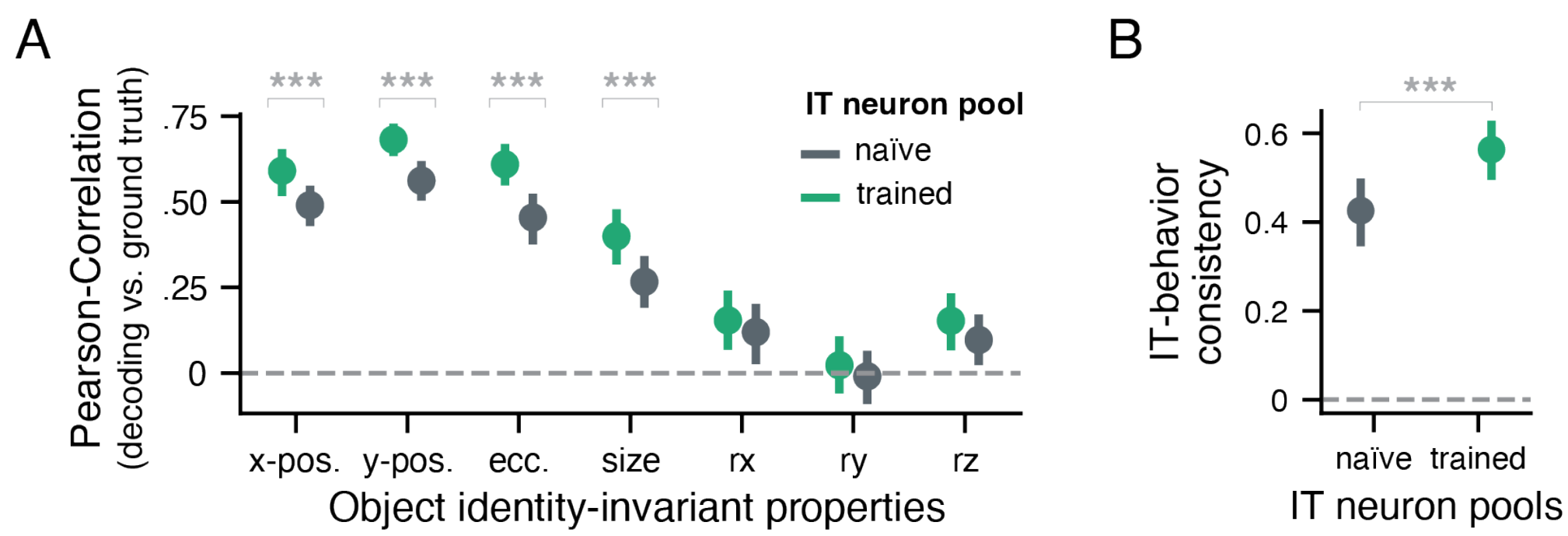

Figure S13. Task training introduces differences in object identity-invariant task decodes and IT-behavior consistency. *(A) Decoding accuracy for object identity-invariant image properties (x-axis) from task-naïve and task-trained IT responses. Accuracy is assessed via a correlation between the ground-truth image latents of the 640 test images and the held-out cross-validated decoder predictions (y-axis). (B) IT-behavior consistency between task-trained behavior and task-naïve or -trained IT-object decodes. IT-behavior consistency (y-axis) refers to the noise-corrected correlation between the image-specific performance estimates. In both panels, error bars depict the 95% confidence interval across 1000 pseudo-random draws from the respective neuron pools. All starred comparisons are statistically significant at p < 0.001 in a two-sided two-sample bootstrap test.*

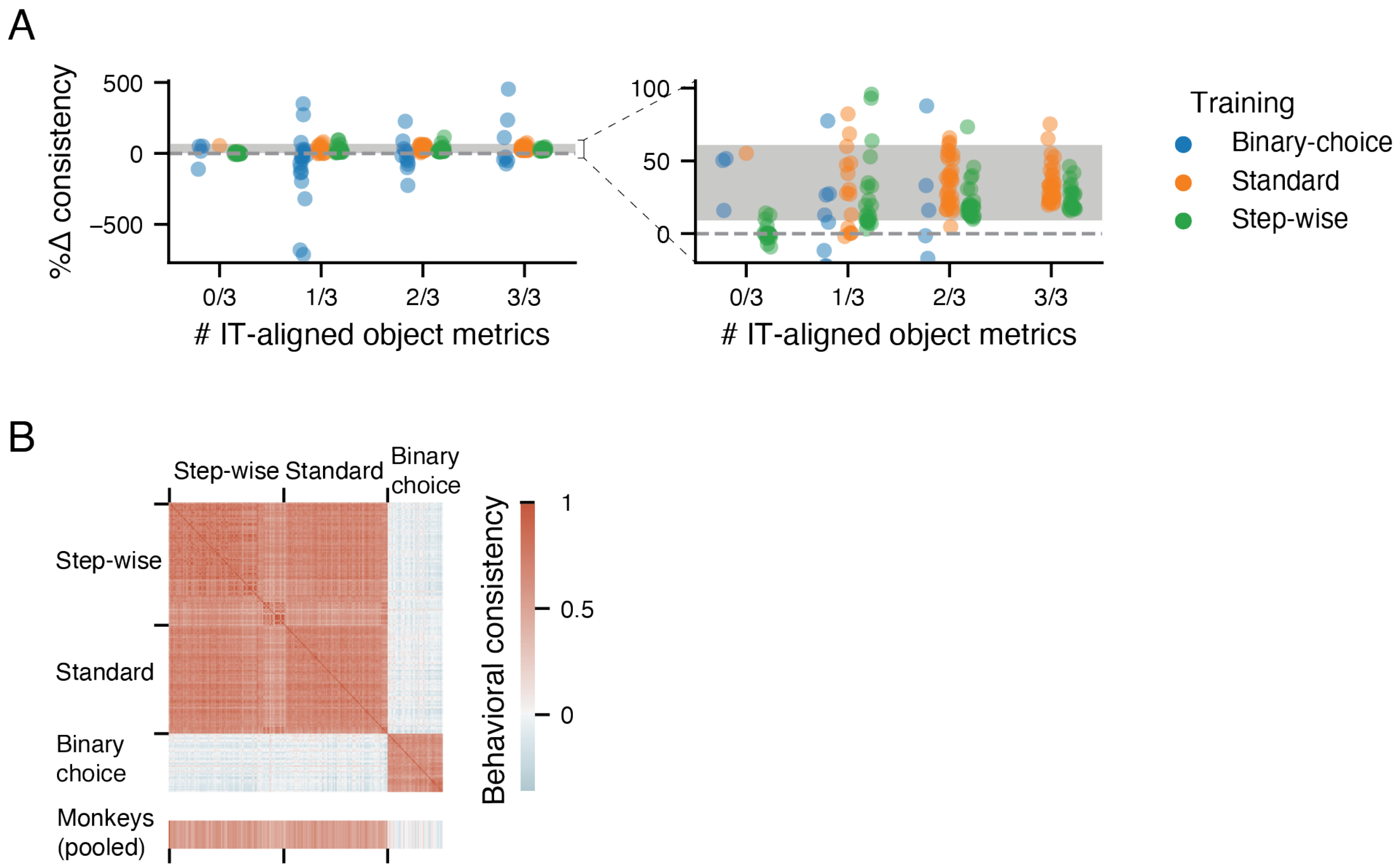

Figure S14. Binary-choice training produces distinct behavioral patterns from other training approaches and monkey behavior. *(A) Binary-choice training predicts the most extreme differences in behavioral consistency. Replotted data from Fig 8D as a function of model training. All figure conventions follow those in Fig 8. (B) Comparison of image-specific behavioral patterns between trained models and monkeys. Noise-corrected correlations (i.e., consistency) between all 208 models and pooled monkey behavior based on image-level behavioral performance across 640 test images. Individual models are ordered according to their training approach. While models trained with step-wise and standard training are highly consistent with each other after training, binary-choice-trained models were only consistent with themselves but not with models from other training approaches. Image-level performance patterns estimated from the behaviors of 3 trained monkeys are consistent with step-wise and standard training but not with models trained with binary choice feedback. The bar height of the monkey data is increased to enhance visibility.*
