# Supplementary material for: Hierarchical optimization predicts plasticity in the macaque inferior temporal cortex following object training
Source: Nat Commun. 2026 Jul 8;17:8434. doi: 10.1038/s41467-026-74816-0 (PMC13478300; doi:10.1038/s41467-026-74816-0)
Supplement: Supplementary file 3 [file 41467_2026_74816_MOESM3_ESM.pdf]

nature portfolio

Peer Review File

Hierarchical Optimization predicts Plasticity in the Macaque Inferior Temporal Cortex following Object Training

Corresponding Author: Dr Lynn Sörensen

This file contains all reviewer reports in order by version, followed by all author rebuttals in order by version.

Version 0:

Reviewer comments:

Reviewer #1

(Remarks to the Author)
In this manuscript, the authors examine how object encoding in area IT changes with object training, and whether those changes can also be observed in object-classifying convolutional neural networks models trained on a specific set of objects. The analyses are sophisticated and rigorously designed in accordance with the ambitious scope but there are a lot of technical details that are hard to grasp without referring to previous publications. Fundamentally, in its current form, it almost seems like a survey of computational techniques without a central motivating question or strong interpretations about how the presented results constrain biological or non-biological learning. One possible solution would be to break up the physiology/behavior observations from the modeling, allowing a better discussion of prior work (as opposed to just saying IT plasticity is “controversial”), and a better discussion of how “good” and “bad” models actually differ and any associated computational implications. There are issues of mapping these results onto previous physiological studies and separate issues regarding mapping onto the neural network literature, and by trying to do both in one paper, both of these mappings are being compromised.

1. There is a lot of pooling going on and it strongly limits how strong an association can be made between physiological changes and learning. If I understand correctly, the zero d' comes from animals that have not received task training, while the 2.5 d' (Fig 1C) comes from a different set of animals that have received training. But for the trained animals there are no measurements of how their performance increases with training, which is likely non-linear and variable between animals. Should IT be involved, than the actual dynamics in one animal's IT should have some match to that animal's behavioral improvement, but in the absence of that, behaviorally responsible changes could be occurring anywhere and anytime (and in different ways and places in different animals) and “trickle” up/down/sideways to IT after the training is largely done (and presumably things like vigilance and reward anticipation are much higher). And at very basic level is there any evidence in the data, either a single unit or population level, of choice signals in the recorded cells? Is the assumption of neuronal independence implicit in all of this pooling justifiable?

2. There's a lot of literature in perceptual learning that deals with the issue of changes in encoding vs changes in readout, from the modeling perspective (Lu/Dosher, Seitz, Biderman), and even physiology (Josh Gold). This seems very relevant to the question of what changes (and where) are responsible for learning, but these works aren't really addressed. On particular relevance is Seitz's use of AlexNet to reconcile a number of seemingly contradictory experimental findings. In this respect, the statement that there are lot of “conflicts” in the literature (line 66) seems like a lost opportunity: can these models help reconcile differences in the experimental literature in any way? For example, could subtle differences in task training or environment exposure when introduced to some ANN result in different internal unit performance?

3. Regarding the issue of changes in encoding vs readout, it would seem the data are clear, that readout has to be dominant factor: you can't get untrained performance (d'=0) without a pool size of more than few neurons, whereas trained performance can be obtained by decoding either untrained or trained IT (with a slight change in pool size). So while decoding performance is improving in IT, it's potentially irrelevant (or largely irrelevant) to explaining the behavioral improvements observed in training. That's fine, and it's still important to understand how IT changes with learning, but its relevance to actual behavior in this task is really questionable. This needs to be made clearer, and there should also be some discussion of tasks or situations in which the observed plasticity might have some demonstrable effect on performance.

4. There's a lot of references to anatomically mapped, but I really don't know what that means. Off hand I can think huge number of anatomical features (feedback, neuromodulation, laminar connectivity, neuronal activity correlations, lateral connections) that seem absent here. What does a non-anatomical layered convolution model look like and how does it differ in performance? Line 652 makes reference to biological constraints: what constraints and do you do any tests in which those constraints were absent? Further unease with regard to mapping artificial neural networks to real ones comes the completely non-biological nature of gradient descent, and the fact the feedback that actually matches the training (binary) is not better than the other feedbacks (and in fact looks fundamentally different in Fig S7).

5. More data is not particularly helpful if it doesn't give rise to insights, and here testing 660 models and finding roughly 10% meet the requirement, with very little discussion about factors or differences that are critical (or even related) to explain the physiological changes, seems to fall into this category. If I want to make a realistic model of IT, how should I proceed? Or is the point that it largely doesn't matter what I do because a large number of models work? If I run 10,000 models and get a 1000 to “work” by satisfying the 3 requirements, what have I learned? Similarly how useful is a mean model (Fig 5D)? If I varied the hyperparameter space differently wouldn't I get different distributions? And if a particular model (say one in the 0/3 category) can explain the physiology, how can that be ruled out? (Maybe IT is like that model with a set of hyperparameters that is governed by physiological/biological constraints).

6. In a related matter, while the 3 requirements makes a nice story, it would appear that the strongest constraint is that of changes in linear decoding performance (which get us from 660 to 75). What's going on here? Is there a reason why that would be true? And although it's a small number, how can improvements in linear decoding not be associated with the other 2 factors? The graphs of Fig 5 implicitly assume that all of the 3 factors are equivalent (the 3 1/3s are all the same, and 3 2/3s are all the same). Is that true and if not, how can they be thrown together? There's a potentially interesting story here (that parallels previous ones) about invariance organically being generated as a consequence of object training irrespective of the nuances of the model or training, but if that's the goal, a lot of these questions need to be cleared up.

7. One important control that is absent in the computational models is any test for layer specificity. That might be difficult given the metric applied for measuring “IT-ness” in naive models is often very broadly distributed (Fig S5). But in combination with the large number of models and feedback that seem capable of explaining physiology it brings up the uncomfortable point that similar results (in terms of change in performance as seen in Figure 5) might be found across a huge number of layers. In this case there would be even less of an ability to associate the artificial NNs with biological NNs and for that matter NN changes with behavior.

(Remarks on code availability)

Reviewer #2

(Remarks to the Author)
The authors’ primary aim was to develop a system-level model of adult brain plasticity during new object learning, using IT cortex as because of its well-established role in rapid object recognition and categorical discrimination. They frame three hypotheses about where learning-related changes occur: all learning-induced adaptations occur downstream of IT (No plasticity); IT itself undergoes task-relevant changes, but to a lesser degree than behavior (Moderate plasticity); IT fully mirrors the magnitude of behavioral improvements (Peak plasticity)
They trained three adult macaques on a novel object-categorization task and compared their IT responses—recorded under matched passive-viewing—to those of three naïve controls. By pooling reliability-matched multi-unit sites, they measured learning-related shifts in single-site selectivity, representational similarity, and linear decodability, and asked whether anatomically mapped, fine-tuned ANN models could predict the observed neural differences. The results revealed clear but quantitatively modest enhancements in selectivity, category clustering, and decodability—decisively ruling out the No plasticity and the Peak plasticity hypothesis, but supporting the Moderate plasticity hypothesis (H1). Convergent ANN simulations further demonstrated that gradient-based optimization in hierarchical networks provides a mechanistic account of these changes.
Overall, the authors combined carefully controlled cohort design, rigorous statistical procedures, and computational modeling to make a strong case for moderate IT plasticity. While their approach convincingly addresses the central question, it also highlights the need to explore how downstream circuits contribute to the remaining behavioral gains.
I have a couple of minor methodological questions that don’t undermine the core findings or preclude publication:
1. The authors focus on the early, feed-forward response (70–170 ms) to maximize signal-to-noise but don’t explain why they did not analyze later post-stimulus epochs. Task-modulated, feedback-driven effects—and true category-related signals in IT—often emerge ≥ 300 ms after onset of the stimulus (e.g., Meyers et al., 2008; McKee et al., 2014). By restricting to this early window, the study may underestimate the full magnitude of learning-induced plasticity and miss late-emerging, abstract category dynamics. It would strengthen the manuscript to clarify why later windows were not examined.
2. The choice to record under passive fixation—intended to equate sensory conditions across trained and naïve groups and avoid confounds from attention, decision-making, or motor planning—is understandable, and the authors rightly concede that comparing separate cohorts prevents tracking how IT representations evolve within the same individuals. Nevertheless, they should justify why they opted for two different groups instead of within-subject, longitudinal recordings, and discuss the trade-offs and practical considerations that precluded a same-subject design. Furthermore, the approach of relying solely on passive viewing may underrepresent the full extent of learning-induced plasticity, since category-selective responses in IT have also be found weaker under passive fixation than during active discrimination (see for instance McKee et al. (2014)).

(Remarks on code availability)

Reviewer #3

(Remarks to the Author)
This paper proposes a computational framework to model the effects of visual categorization learning on ventral visual stream responses. It combines an analysis of multi-unit recordings in the inferior temporal (IT) cortex of two groups of monkeys, one group of 3 monkeys trained in an object categorization task and another group of 3 untrained monkeys, and computational modeling. Extending previous studies of visual learning in macaque IT, they show an increased object selectivity in the trained compared to the untrained monkeys. This increased selectivity is, although statistically significant, small compared to the behavioral improvement, which aligns with previous studies. Then, and this I found the most interesting part, they trained, using the same images as in the electrophysiology study, a set of deep artificial neural nets (ANNs) and compared the training-induced changes of these ANNs to those observed in IT. They found that some, but not all, ANNs modeled the IT changes. These "IT-like" ANNs also modeled a training-induced increased selectivity for (some) stimulus transformations that are orthogonal to object category (which is a new finding by itself), and changes in the consistency of behavioral and IT-response-based object classifications.
I enjoyed reading this very well-written paper. The main analyses are thorough and carefully conducted, and they are further supported by appropriate control analyses (e.g., region-specific analyses in PIT, CIT, and AIT). Overall, the neural data extend and agree with previous studies showing increased selectivity in ventral visual stream areas V4 and PIT with visual perceptual learning and some studies in macaque IT showing changes in selectivity with shape or categorization learning (e.g., the recent study by Pearl et al., J Neurosci, 2024; also using Utah arrays for recording and decoding as analysis). The main advance offered by the present study is the meticulous computational modeling of the training effects seen in IT. That said, I have some issues and questions concerning the technical aspects of the methods/design and the data interpretation of this interesting study that need to be addressed.
Main comments.
1. A general comment to start: the authors perform several control analyses, of which the outcomes are consistent with the hypothesis that the differences in neural selectivity between trained and naive subjects are induced by training instead of due to individual differences, electrode locations, or anatomical differences. However, between-monkey designs to assess learning effects, especially with a small number of subjects and different electrode locations among subjects, are tricky, especially without proper controls. For instance, it would have been great to have an untrained stimulus set presented in both groups during the fixation task while measuring the responses and show that the changes seen after object learning were not present for this control set of images. This would have strengthened the conclusion that the reported IT response differences between the trained and naive groups are due to training.
2. In their task, the monkey must segment the object from the complex scene background. Part of the training effects may reflect learning this object–background segmentation. It is possible that during the fixation task in which neuron responses were tested, the trained monkeys were segmenting the objects from the background more efficiently, because of the training, than the untrained monkeys, and this may have resulted in the observed changes in IT responses. This could explain why they find both an increased (invariant) object decoding and an increased decoding of the category-orthogonal stimulus properties, such as of object position and size – better object segmentation in the trained group can result in both effects. This possibility should be discussed (they can relate their findings to the literature on the effects of learning on object segmentation). A related question is to what extent the general learning effects (changes in object and object-invariant selectivity) require explicit training or result from the extensive stimulus exposure during training? Would passive exposure to the trained stimulus set produce similar changes in IT? This can be discussed.
3. Related to the previous comment: In the categorization task, the location, size, and rotation of the object are irrelevant to the task. Based on previous literature (e.g., Sigala and Logothetis; De Baene et al), I would have predicted little if any effect of training on IT selectivity along these irrelevant, category-orthogonal dimensions, which is not what they see (see main point 2 above). It is interesting to see that this was also not true for the ANNs – at least those that are IT-like. This counter-intuitive result deserves a comment/discussion.
4. The reliability distributions for the trained and untrained neural data are very different, with lower reliabilities (many close to zero) for the trained sites. Any explanation for this difference? Although the authors matched reliabilities between the two groups of sites, this again raises the issue (see comment 1 above) of the possibility that differences unrelated to training can explain the "general" training effects (for relevant and irrelevant dimensions) seen in the present study. Also, a reliability criterion of 0.3 is pretty low. How robust are the training effects when changing (increasing) this reliability criterion?
5. The authors examined several measures related to IT object selectivity, which is excellent. However, were there differences in overall (net or raw) firing rates between the two groups? Were responses stronger (or weaker because of familiarity with the objects) in the trained compared to the untrained groups? And if so, can this have impacted the selectivity changes (e.g., decoding)? Perceptual learning studies have reported decreased response variability (Fano factor) upon learning. Was this also the case here?
6. The authors state that the neural changes were much smaller than the behavioral changes. But I could not find where the behavioral results of the naïve, untrained group are coming from. Were these the initial performances of the trained monkeys? Please clarify. This is important since, as discussed in the perceptual learning literature, learning a perceptual task can be conceived of as consisting of different (overlapping) phases, with in the first phase the animal learning the basic task (the task rules) and later a more "perceptual" phase. It is likely that IT is not involved in basic task learning but is (partially) involved in the "perceptual" phase. If stimuli are relatively easy to separate perceptually, then most of the behavioral task improvement will come from task learning, and thus a large gap between behavioral improvement and IT selectivity changes is expected. Hence, how was the behavioral improvement assessed: describe what was taken as "naïve" data (and discuss its implications) and show learning curves. Also, how much did the behavioral improvement differ between animals, and were these interindividual differences related to neural changes (although this might be hard to

assess in a between-subject design and with anatomical differences in recording locations between subjects)?
7. The data on the orthogonal/identity invariant dimensions are interesting, especially their (average) consistency with the IT-like models. However, it appears that object-invariant decoding of the rotations was low (Figure S8) and lower than in their previous study (Hong et al.). Intuitively, the poor decoding of object-invariant rotation makes sense since rotating an object alters its 2D shape in the image. The latter is not the case for size and translation changes, which may make them easier to decode. Interestingly, some ANN IT-like models showed large training changes for rotation, some negative and some positive. Any explanation? Was there a correlation between these training changes for the models across rotations?
8. The higher consistency between behavior and trained IT neural data is interesting. Is it possible to examine the consistency of the naïve/trained IT data with behavioral data obtained at different points during the behavioral training? When do naïve versus trained IT-behavior consistency diverge during learning (related to point 6)?
9. The authors should discuss in more detail the impact of different categorization paradigms on learning effects in the ventral visual stream and where their task is situated in this "population" of tasks. I expect that the kind of changes one will see in the ventral stream (V4 / IT) and the models depend on the nature of the categorization paradigm. For instance, in some studies, the subjects learned to discriminate small differences (e.g., orientation or contrast) between well-visible stimuli, in others to discriminate noise-masked stimuli, and in other studies to categorize multiple stimuli in the same group with small differences at the category boundary, etc. In the present study, subjects needed to categorize 10 objects rendered at different orientations, locations, and sizes, and presented upon complex scenes. This is like subordinate object classification, but with different scene backgrounds. Which factor(s) is (are) determining the performance and learning effect in this task: the segmentation from the complex image background and/or the position-, size-, and rotation-invariant object categorization?
Minor comments.
1. When discussing the older literature in the Introduction, Results, and Discussion, the authors should avoid lumping together different tasks or categorization paradigms, and instead clearly point out the distinctions (see main comment 9 above). In addition, they should integrate their findings and the older literature with the visual perceptual learning work in the ventral stream (including PIT studies), which is highly relevant for addressing visual learning. A related point is that it is not clear what exactly is meant by "learning new objects" (Introduction; line 5).
2. Figure 2E: The authors write that they computed Euclidean distances between images, but the left figure panel shows "corr(r)", suggesting a correlation-based distance metric. Please correct.
3. Line 263: "Data from B and C in annotated for comparison". Please clarify what is meant here.
4. Line 569: "This highlights the role of anatomical considerations in future studies." It was not obvious to me what is meant here with "anatomical considerations". Please clarify.
5. Line 586 and following: "A direction for future research is to test whether the magnitudes of neural changes induced by object vs. category training exceeds what would be expected based on the visual dissimilarities between objects." This sentence is not clear to me (object versus category training…) – please clarify.
6. Line 726: typo?: canonical.
7. Line 779: Please provide the bandpass filter settings (and filter order).
8. Line 780: What is the difference between multi-unit activity and multi-unit spikes?
9. Were the data normalized per unit before computing Euclidean distances?
10. Line 854: "The inputs to the regression models were scaled based on the training data split." Scaled = normalized (z-scored?)?
11. The manuscript states in several places that De Baene et al. did not find an effect of categorization learning in IT. This is inaccurate. That study did report a categorization learning effect in IT—albeit small—restricted to the task-relevant feature dimension when comparing pre- and post-training recordings. I recommend revising these statements accordingly.

(Remarks on code availability)

Reviewer #4

(Remarks to the Author)
This is an interesting study that investigates how object learning in a visual task induces plasticity in the macaque inferior temporal cortex, a critical region for visual object recognition. The authors trained six monkeys on a delayed-match-to-sample task involving eight object categories, with images carefully designed to systematically test object recognition in IT cortex. Neural activity was then recorded using chronically implanted multielectrode arrays across multiple IT subregions in both trained and naïve animals. The results showed that training enhanced object selectivity in the IT cortex. The authors presented this effect using multiple complementary approaches, indicating improvements both at the single-site level and at the population level, including representational geometry and linear decodability. The authors then employed hierarchical neural network models to simulate IT plasticity. Unfortunately, despite the strengths of the study, the manuscript still requires substantial revisions to address key issues.

Major concerns:
The manuscript provide no information about the training phase, such as the total number of sessions, number of trials per session, the overall duration of training. These details are critical, especially in a study focused on plasticity, since the magnitude and nature of neural changes might closely tied to training intensity and time course. Moreover, it would be important to report the behavioral learning curves, error rates, and performance progression across training sessions. These information must be provided for each animal separately.
Similarly, the description of neural recordings lacks important information. The manuscript should provide details on the number of recording sessions, the number of images presented per session, and quality metrics such as signal-to-noise ratio

for each electrode and the yield of each array. Information on the timing of recordings relative to the training phase (e.g., early vs. late training) should also be reported. As with the behavioral data, these details should be provided for each animal and each array separately, since variability across subjects and recording sites may strongly influence the interpretation of the results. Moreover, there is no analysis reported within each animal. All of the main analyses should also be presented at the single-animal level. I understand that such analyses might not reach statistical significance due to limited power, but they are essential for evaluating replicability and robustness of the reported effects.
The central conclusion from the neural data relies heavily on Figure 2L-M, which compares delta-d' between IT decoding and behavior. However, the approach used to derive these results raises several concerns. First, the delta-d' in IT is obtained by fitting a log function to the decoder performance as a function of the number of IT sites, but it's not clear how exactly delta-d' is calculated. Moreover, this fitting procedure introduces strong assumptions, including that the readout of IT activity in the brain is linear and that decoder performance continues to increase logarithmically with larger sample sizes. However, the neural decoding performance may reach a ceiling, and alternative fits (e.g., saturating or sigmoidal functions) may be more biologically plausible. The authors should justify their choice of function and demonstrate that their conclusions are robust across different fitting approaches. Second, even accepting the log fit, Figure 2L implies that the performance of an IT-based linear decoder would reach the monkeys' behavioral range with approximately 200 neurons and would exceed behavioral performance with a greater number of neurons even in naïve animals. This finding is difficult to reconcile with the claim that behavior can be directly compared to a linear readout of IT activity in order to reject H1 or H2.
Most of the p-values and confidence intervals are presented without corresponding effect sizes or sufficient context for the underlying statistical analyses.

Minor concerns:
All reported p-values should include a leading zero before the decimal point

Figure 1C and 1M: on the y-axis, d′ is labeled as "Accuracy" which is an unusual description for d'. "Performance" would be a better choice. Moreover, the plot currently presents only normalized d' (delta-d'). Please add a bar plot showing performance before and after training for each animal separately, as presenting the absolute values in the first plot would be more informative.

Line 58: the term "perception-action" has not been used elsewhere in the manuscript and requires clarification.

Line 151: subfigure (M) is presented as the right panel in subfigure (L).

Figure 2J: numbers on the y-axis should include a leading zero before the decimal point.

Figure 2M: this is one of the main plots of the paper summarizing results in the hypothesis space (H0, H1, H2). It would be more informative if plotted with greater detail (e.g., presenting data points for each animal separately, rather than only pooled results).

Lines 223-232: the paragraph beginning with "How do these observed training-induced differences compare to differences in interindividual variation?" seems intended to justify the use of pooled data across subjects. If so, the term "between-group" at the end should be revised. In any case, the paragraph is vague and should be clarified.
Line 243: "Addtionally" should be "Additionally"
Line 243: differences between areas may be due to variation in recording quality and signal-to-noise ratio. More controlled measures are needed.

Line 269: wording should be corrected to "we would expect."

Figure 6D: it is unclear what the gray band (95% CI of neural data) represents. A 'good model' should be closer to the actual performance (likely the median of the CI). If the intent is to show similarity, a scatter plot illustrating correlations might be more effective.

Figure S5 legend: "sqaures regression" should be "squares regression"

(Remarks on code availability)

Reviewer #5

(Remarks to the Author)
I co-reviewed this manuscript with one of the reviewers who provided the listed reports. This is part of the Nature Communications initiative to facilitate training in peer review and to provide appropriate recognition for Early Career Researchers who co-review manuscripts.

(Remarks on code availability)
i cannot find the codes

Version 1:

Reviewer comments:

Reviewer #1

(Remarks to the Author)
The revision is very responsive and clarifies a number of issues. It's now much clearer at the outset that the results fall into the “some plasticity but not fully able to explain behavior” camp. The discussion as well is significantly improved with much clearer statements about implications and potential future directions. My remaining concerns are certainly not disqualifying and mainly affect some new results that appear the responses and supplementary figures that deserve better discussion and integration into the main body.

1. There are 3 points that I believe are rather central and should not be sequestered to supplementary material. The first is learning dynamics and its gradual and irregular natures, consistent with gradual changes in representation (Fig S2). The second is Fig S8 showing specificity of learning with regard to trained objects. The third is S13 which I find a very nice approach for address the observed changes in a high dimensional space. With regard to the figure however, I fell like the discussion which focuses solely on manifold size is a little too brief. Rather I think it's the combination on increases in inter-cluster distances without changing cluster sizes that should be emphasized with the 3 metric matches: intuitively (unless I'm misinterpreting) that would imply better potential decoding, whereas increases in all distances (both within and between clusters) would not.

2. Another important point is the specificity of changes to IT-like layers in the models. While this is anecdotally addressed in S11, I think it deserves a more thorough treatment (and accordingly potential inclusion into the main body). First, rather than taking 3 individual layers and looking at IT specificity, represntation strength, and decoding distributions, I would instead for each of these report the percentage of models that fall within the realistic range of those parameters for all the layers. Just showing a relative simple one, like AlexNet would be fine here. But also of critical interest are the generalizations to untrained parameters shown in Figure 6. If I take the 3/3 models I would expect very poor generalization for early layers (and maybe too much for high layers?).

3. In the original submission I found the mismatch of the binary choice training to behavior/physiology puzzling, and now with more discussion and results, it seems even more puzzling. If I understand correctly, the extreme changes in behavioral consistency (Fig 6F), the ones far greater than would be expect from the IT data), are selectively happening for binary choice training? (If this is true, it might help to color the points in this presentation according the updating algorithm). Figure S15 shows that binary choice is distinctive for the other algorithms AND inconsistent with behavior. Finally, Figure S12 shows that decoding often completely fails (d'=0) with binary choice. Taken altogether, it seems like the most biological plausible updating is a complete disaster. Why? Does the sparseness and locality of reweighting mean changes get “trapped” in local minima? If i did a “slower” probabilistic reweigtting (or one with errors in assignment) would that help?

4. I believe the motivation in the introduction could flow a little better. Right now it identifies a nice “controversy” with some IT results suggesting plasticity and some suggesting none or very little. It would be helpful here to identify the particular choices of the experiment design here and why they might be relevant for reconciliation. For example, was relatively poorly physiological sampling a concern for some studies? Is the diversity of backgrounds used here vital? How about the number of categories? In short, a clearer delineation of previous limitations and how this study addresses those would be very helpful.

(Remarks on code availability)

Reviewer #3

(Remarks to the Author)
The authors have responded to all of my concerns and even performed an additional control experiment, suggesting that the neural “training” effect is restricted to the trained stimuli (albeit based on a single-monkey comparison), which I appreciate. Upon rereading the manuscript, I have four remaining (minor) issues that should be addressed.

1. The authors now present data from individual animals (Figure S6) and conclude that the same trend is observed at the individual-subject level as in the pooled data. While this is the case for the d′ metric, there is considerable variability across subjects for the other two metrics (the three data points of the naïve group are not consistently below those of the trained group). I therefore suggest softening the claim that these data “indicate robust training effects at the individual level.”
2. The inclusion of a table listing the recording sessions per monkey is informative. I do, however, have a question regarding the pooling of data across individual sessions: how confident are the authors that data from different sessions are statistically independent, given that recordings may have been obtained from the same units across multiple days using the chronic implants? Please comment on this issue.
3. Please provide the recording location(s) for the newly included Monkey P.
4. “Monkeys completed hundreds of trials per session from the start of training, demonstrating familiarity with the task structure…” The authors should provide additional evidence that they were familiar with the task structure. That they completed hundreds of trials indicates that they were motivated to do/try something. I suppose they were trained before to perform the task with other stimuli. Please clarify.

(Remarks on code availability)
-

Reviewer #4

(Remarks to the Author)
The authors have addressed my previous concerns by adding behavioral learning curves, additional analyses at the individual level, and clarifying the interpretation of the IT-behavior relationship. I have no remaining concerns and consider the manuscript suitable for publication.

(Remarks on code availability)

Reviewer #5

(Remarks to the Author)
I co-reviewed this manuscript with one of the reviewers who provided the listed reports. This is part of the Nature Communications initiative to facilitate training in peer review and to provide appropriate recognition for Early Career Researchers who co-review manuscripts.

(Remarks on code availability)

Version 2:

Reviewer comments:

Reviewer #1

(Remarks to the Author)
I am fully satisfied with the manuscript and thank the authors for their thoughtful responses and changes.

(Remarks on code availability)

Reviewer #3

(Remarks to the Author)
The authors have addressed all of my remaining concerns. I congratulate them on this nice work.

(Remarks on code availability)
-

Open Access This Peer Review File is licensed under a Creative Commons Attribution 4.0 International License, which permits use, sharing, adaptation, distribution and reproduction in any medium or format, as long as you give appropriate credit to the original author(s) and the source, provide a link to the Creative Commons license, and indicate if changes were made.
In cases where reviewers are anonymous, credit should be given to 'Anonymous Referee' and the source.
The images or other third party material in this Peer Review File are included in the article's Creative Commons license, unless indicated otherwise in a credit line to the material. If material is not included in the article's Creative Commons license and your intended use is not permitted by statutory regulation or exceeds the permitted use, you will need to obtain permission directly from the copyright holder.
To view a copy of this license, visit https://creativecommons.org/licenses/by/4.0/

Reviewer #1 (Remarks to the Author):

In this manuscript, the authors examine how object encoding in area IT changes with object training, and whether those changes can also be observed in object-classifying convolutional neural networks models trained on a specific set of objects. The analyses are sophisticated and rigorously designed in accordance with the ambitious scope but there are a lot of technical details that are hard to grasp without referring to previous publications. Fundamentally, in its current form, it almost seems like a survey of computational techniques without a central motivating question or strong interpretations about how the presented results constrain biological or non-biological learning. One possible solution would be to break up the physiology/behavior observations from the modeling, allowing a better discussion of prior work (as opposed to just saying IT plasticity is "controversial"), and a better discussion of how "good" and "bad" models actually differ and any associated computational implications. There are issues of mapping these results onto previous physiological studies and separate issues regarding mapping onto the neural network literature, and by trying to do both in one paper, both of these mappings are being compromised.

We appreciate the reviewer's attention to detail and their thoughtful review of our work. We hope that we can alleviate their concerns in our responses below.

1.1. There is a lot of pooling going on and it strongly limits how strong an association can be made between physiological changes and learning. If I understand correctly, the zero d' comes from animals that have not received task training, while the 2.5 d' (Fig 1C) comes from a different set of animals that have received training. But for the trained animals there are no measurements of how their performance increases with training, which is likely non-linear and variable between animals. Should IT be involved, than the actual dynamics in one animal's IT should have some match to that animal's behavioral improvement, but in the absence of that, behaviorally responsible changes could be occurring anywhere and anytime (and in different ways and places in different animals) and "trickle" up/down/sideways to IT after the training is largely done (and presumably things like vigilance and reward anticipation are much higher).

We thank the reviewer for the opportunity to clarify these aspects. Indeed, we assumed that the task performance of naïve subjects is at a d' of zero (chance-level), whereas the trained estimate comes from the trained group. We have now estimated the task performance of the trained group at the beginning of their object training.

Conceptually, the task training can be divided into two overlapping phases: the acquisition of the task structure and the discrimination of the training objects. We would not expect that the task structure training impacts IT. In contrast, we do expect that learning about these specific objects could affect IT responses. To better tease these aspects apart, we analysed how two of the trained subjects learned about the objects after they were already familiar with the task structure (Fig S2; the data from the third subject was not recorded). Based on this, we see no indication of large interindividual differences across subjects (Fig S2B).

Unfortunately, we cannot link these behavioral time courses to the IT measurements, as no task-naïve IT recordings are available for these subjects.

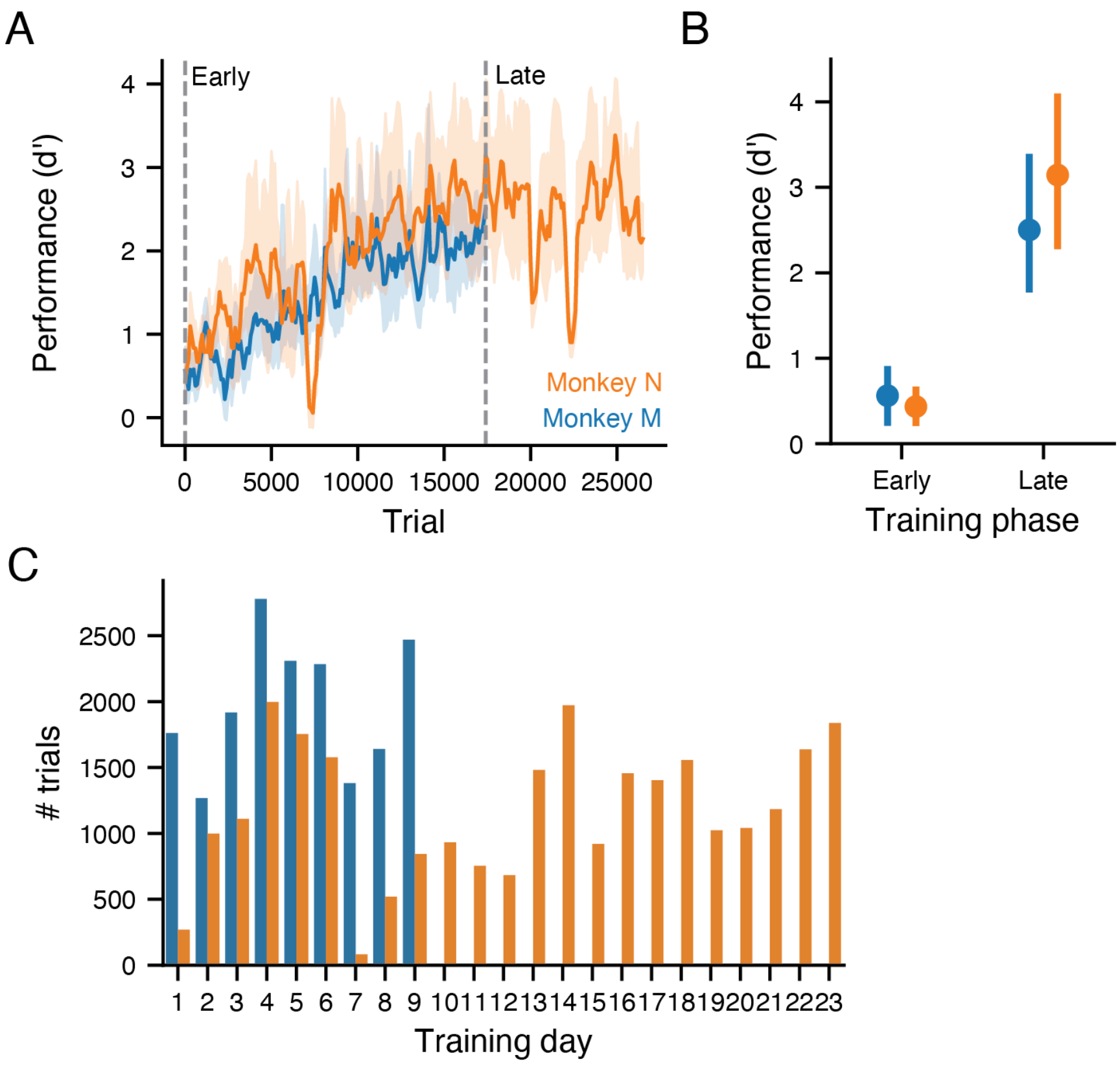

Figure S2. Object discrimination performance improves gradually during training. (A) Learning curves task-trained monkeys. Performance is estimated using a sliding window of 500 trials for each object; the x-axis indicates the starting trial of each window. Shaded areas show 95% confidence intervals of the mean bootstrapped across the eight objects. Dashed vertical lines mark the early and late training periods analyzed in panel B. (B) Comparison of early versus late training performance. Monkeys showed near-chance performance early in training but robust discrimination late in training (trial window: 17400-17900 for both subjects), demonstrating significant improvement over the training period. (C) Number of completed trials per session for each subject. Monkeys completed hundreds of trials per session from the start of training, demonstrating familiarity with the task structure despite initial unfamiliarity with the specific training objects (corresponding to low early performance in panel A).

We have now included this figure as a supplementary figure and clarified this aspect in the manuscript:

“Substantial perceptual learning was required for successful performance: Subjects, who were already familiar with the task structure, displayed low performance at the onset of the object training (d’= 0.50) and only gradually improved their discrimination abilities over the course of the training (d’ = 2.82, Fig S2), thus indicative of object-specific learning.”

Results - Task training enhances task-relevant information in IT

And at very basic level is there any evidence in the data, either a single unit or population level, of choice signals in the recorded cells?

We agree with the reviewer that demonstrating a link between IT activity and behavioral choice is critical for interpreting the relevance of the observed neural changes. In the present manuscript, our primary goal was to characterize training-induced changes in IT representations; however, we have previously shown, and continue to find, that IT population activity in the 70–170 ms window carries information that is predictive of behavioral choice. Specifically, in prior and ongoing work (including a manuscript currently in preparation), we constructed decoders based on IT population vectors averaged over the 70–170 ms post-stimulus window and evaluated their ability to predict behavior on a trial-by-trial basis. The logic of this analysis is as follows: if IT activity is causally relevant to choice, then a decoder trained on IT responses should predict the animal's choice on individual trials better than chance, and this prediction should depend on the correct alignment between the neural response and the corresponding choice. Consistent with this prediction, we find that when trial-by-trial IT responses are paired with the correct behavioral choices, the decoder predicts choices with high accuracy (≈0.65). In contrast, when IT responses are deliberately mispaired with choices from other trials, decoding accuracy drops to chance levels (≈0.5). The trial-by-trial choice prediction analysis was restricted to a subset of trials in which the test image contained two objects and both of those objects were present as choice options. This restriction was intentional: under these conditions, the visual stimulus itself is ambiguous with respect to the correct choice, and predicting behavior requires sensitivity to trial-specific neural fluctuations rather than stimulus identity alone. This result demonstrates that trial-specific fluctuations in IT population activity are informative about the animal's choices, rather than merely reflecting stimulus identity or task structure. Although these analyses are part of a separate manuscript under preparation and therefore not included in full detail here, we now include a summary bar plot illustrating this comparison for two monkeys below (two monkeys, M and N, from the task-trained group). This plot directly visualizes the contrast between correctly paired and mispaired trial–choice decoding, providing additional evidence that IT population activity in the analyzed time window is behaviorally relevant.

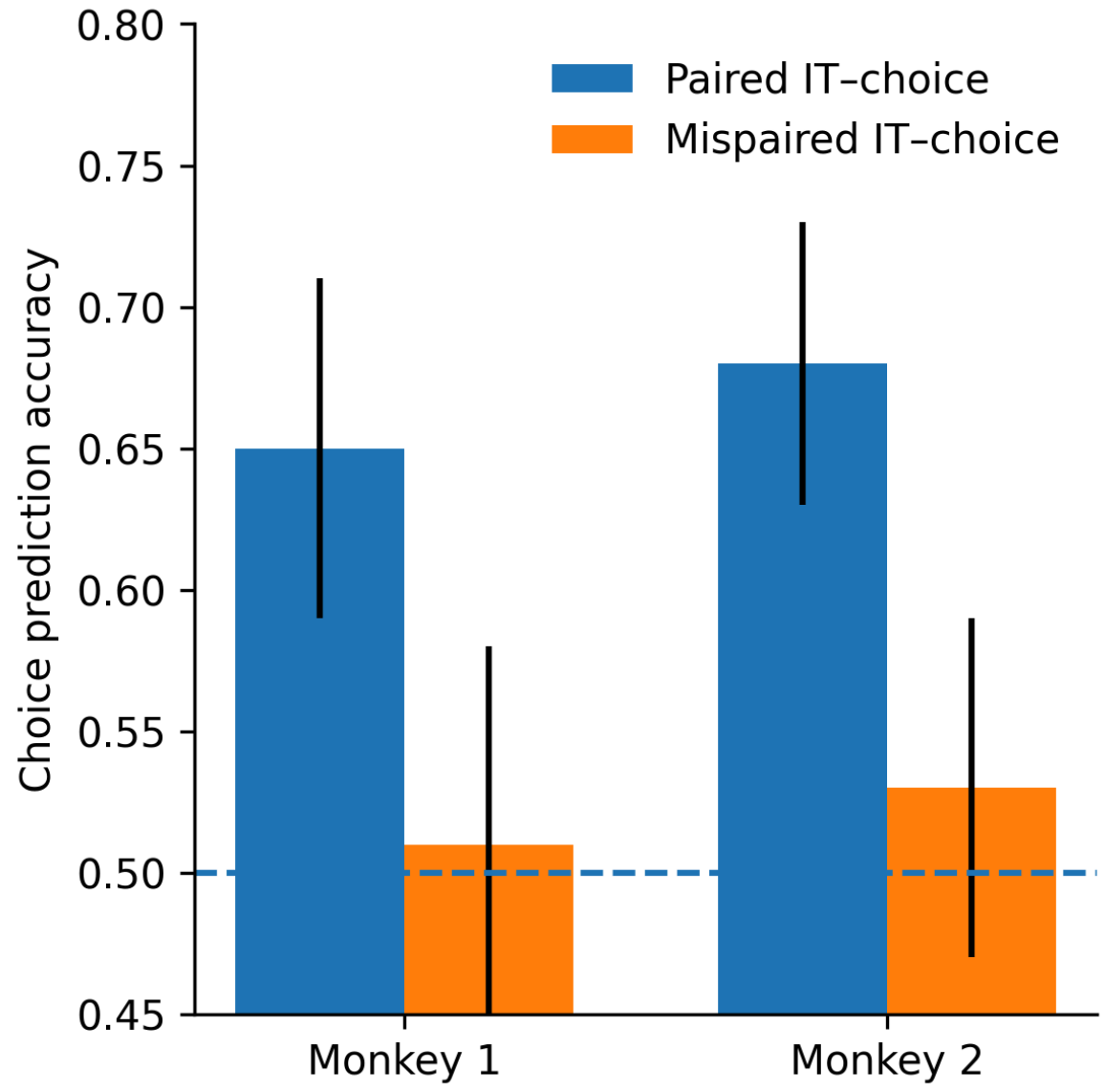

Figure R1. IT population activity predicts behavioral choices on a trial-by-trial basis. Decoding accuracy for predicting behavioral choices from IT population responses (70–170 ms post-stimulus window) in two task-trained monkeys (M and N). Decoders were trained on IT population vectors and tested on their ability to predict choices on individual trials. Paired (blue): IT responses paired with the actual behavioral choice from the same trial. Mispaired (orange): IT responses paired with choices from different trials as a control. Successful decoding required sensitivity to trial-specific neural fluctuations. Decoding accuracy for correctly paired trials significantly exceeded chance (dashed line at 0.5), whereas mispaired trials fell to chance levels, demonstrating that moment-to-moment fluctuations in IT population activity are informative about behavioral choices.

Is the assumption of neuronal independence, implicit in all of this pooling, justifiable?

We would like to clarify that our analyses do not assume neuronal independence. Rather, we assume that there exists shared variance at the neuronal population level across individuals of the same training status (task-trained vs. task-naïve). The goal of our pooling procedures is not to treat recording sites as independent units, but to estimate properties of an underlying population response characteristic of each training group. To this end, we constructed sampling procedures to account for potential confounds such as the number of recording sites per subject and their respective reliability (Fig. S4). These sampling procedures also enabled us to construct appropriate hierarchical null hypotheses to further contextualize the effect sizes of our findings (Fig. S7) and to ensure that they are not due to a single subject or specific recording sites. By repeating our pooling procedure many times, we ensure that our conclusions are not reliant on any individual site or individual, but rather are a robust signature of the population responses within that group. We clarify this in our methods section:

“We aimed to assess group-level differences between task-trained and -naïve recordings at the population level. To ensure that potential differences did not stem from other factors, such as a difference in the number of sites in every group or reliability, we analyzed the recording sites in pseudo-random pools drawn as

bootstraps from all recording sites (within a given group). We thereby assume that any recording site is a sample from the underlying population response and test the hypothesis that this population is changed due to task training."

Methods - Neural and behavioral data analyses - Neural recording quality metrics and pooling

1.2. There's a lot of literature in perceptual learning that deals with the issue of changes in encoding vs changes in readout, from the modeling perspective (Lu/Dosher, Seitz, Biderman), and even physiology (Josh Gold). This seems very relevant to the question of what changes (and where) are responsible for learning, but these works aren't really addressed. On particular relevance is Seitz's use of AlexNet to reconcile a number of seemingly contradictory experimental findings. In this respect, the statement that there are lot of "conflicts" in the literature (line 66) seems like a lost opportunity: can these models help reconcile differences in the experimental literature in any way? For example, could subtle differences in task training or environment exposure when introduced to some ANN result in different internal unit performance?

We agree with the reviewer that computational modeling can unlock systematic comparisons and address prior conflicts in the literature. In this project, we focused on the variety of reported effect sizes for plasticity in IT. Our approach was inspired by Seitz et al., as we mentioned in the introduction. We hope that our approach will invite future studies. We now also highlight this in the discussion:

"Based on our simulations, we reason that differences in the visual features of the training stimuli, their linear decodability in IT before training, and an animal's trained performance after training may have contributed to the diversity of effects sizes reported in prior studies (see Op de Beeck & Baker, 2010 for a review), with some studies reporting sizable changes with task training (Baker et al., 2002; Freedman et al., 2003, 2006; Kobatake et al., 1998; Logothetis et al., 1995; Pearl et al., 2023; Sakai & Miyashita, 1994; Sigala & Logothetis, 2002; Adab et al., 2014) and others finding small or absent changes (De Baene et al., 2008; Op de Beeck et al., 2007, 2008; Vogels & Orban, 1994). Accounting for these factors when comparing studies and generating training-specific computational predictions can help reveal the circumstances that drive or limit IT plasticity during task training in future studies."

Discussion - Adopting a systems-level perspective clarifies IT's role in object learning

We fully agree that our findings have important parallels with prior modeling work, and we now make this connection explicit in our discussion:

"A key insight from our study is that object discrimination training changed IT responses to a moderate extent, yet the magnitude of these differences did not fully account for the behavioral improvements observed after training (Fig 2M). This pattern of results rejected two initial hypotheses (Fig 1E): (1) That IT does not change at all, since IT's responses already held sufficient visual representations to support performance on the trained task (H0) and (2) that IT responses serve as a direct readout for behavior and, in turn, IT would reflect any changes apparent in overt behavior (H2). Our hypotheses can be loosely mapped to prior work on the effect of training on the encoding and readout of sensory inputs, typically focusing on early visual processing (e.g., Dosher & Lu, 2017). In this view, H0 corresponds

to a learning process that leaves the encoding unaltered and only achieves behavioral changes via changes to the downstream readout (e.g., Dosher & Lu 1998). Conversely, H2 describes a process that emphasizes changes in the encoding that enable a linear readout for the trained behavior (e.g., Karni & Sagi, 1991; Yan et al., 2014). Contextualizing IT's difference with those in behavior enabled us to show that neither of these scenarios applied: IT's responses were enriched in favor of the trained objects, indicative of a change to IT's encoding, while at the same time, not differing to the same extent as behavior. These results support a middle ground, in line with H1: IT undergoes functional encoding changes, but most plasticity occurs downstream of IT."

Discussion - Adopting a systems-level perspective clarifies IT's role in object learning

We now also clarify that, complementary to these prior works, we focused more on quantitative predictions of the effect sizes specific to our recorded sites and behavioral measurements, grounded in the specific stimuli and tasks:

"To understand how such task-specific factors shape IT encoding, we developed a computational framework to simulate the plasticity needed at the level of the IT cortex to support the task performance shown by the trained monkeys. These simulations revealed that the magnitude of IT changes across various metrics naturally followed from its functional role within the visual hierarchy. In other words, when we modeled IT as part of a larger network that gradually changes in a distributed fashion to optimize task performance, the models reproduced both the direction and magnitude of differences observed in IT responses. This perspective indicates that IT's plasticity will depend on the training stimuli, the task demands (i.e., the similarity between objects, the number of discriminations), IT's functional state before training, and a subject's task performance after training. This view roughly aligns with proposals of perceptual learning in IT as part of a hierarchy of representations (Riesenhuber & Poggio, 1999; Sheinberg & Logothetis, 2002; Wenliang & Seitz, 2018), but critically, we here turn this proposal into a testable hypothesis grounded in image-computable models and task performance."

Discussion - Adopting a systems-level perspective clarifies IT's role in object learning

Regarding the reviewer's question about whether subtle differences in task training or environmental exposure would produce different outcomes: our results show that multiple combinations of model architecture, training approach, and hyperparameters can yield IT-like plasticity (Fig S12). This points to the fact that the type of changes that IT undergoes cannot be distilled into a simple external factor. This does not imply that "anything works," but rather that IT-like plasticity occupies a constrained region of representational change space, whose detailed mechanisms need further causal exploration. Furthermore, we have now also added a new, potential alternative perspective examining the geometric changes characterizing IT-like models (see explanation and additional figures in the reply to point 1.6).

1.3. Regarding the issue of changes in encoding vs readout, it would seem the data are clear, that readout has to be dominant factor: you can't get untrained performance (d'=0) without a pool size of more than few neurons, whereas trained performance can be obtained by decoding either untrained or trained IT (with a

slight change in pool size). So while decoding performance is improving in IT, it's potentially irrelevant (or largely irrelevant) to explaining the behavioral improvements observed in training. That's fine, and it's still important to understand how IT changes with learning, but its relevance to actual behavior in this task is really questionable. This needs to be made clearer, and there should also be some discussion of tasks or situations in which the observed plasticity might have some demonstrable effect on performance.

We agree that this issue warrants more extensive discussion. While we also agree that changes in the readout are likely an essential part of learning our studied behavior, the evidence from our analyses strongly suggests a functional role for changes in IT encoding. There are three lines of evidence:

(1) **Decoding analyses:** A readout-only account would predict no changes in the encoding of the training objects. By comparing the increase in linearly decodable information to that for objects that were not part of the training, we see that these increases in decodable information were particularly pronounced for the trained as compared to the novel objects (Fig S8, an additional analysis performed in response to Reviewer 3, point 3.1). This supports the notion that IT responses in the task-trained group were reconfigured in service of an improved readout for downstream areas and that this was specific to the trained objects. While theoretically, these changes in decoding may still be epiphenomenal, it seems counterintuitive that these epiphenomenal effects should not extend to the novel objects.

(2) **IT-behavior consistency:** We demonstrated in Fig S14B that the trained passive IT responses were more consistent with the animal's behavior at an image-by-image grain. This adds further evidence to our second analysis presented in point 1.1, showing that this link is even more pronounced during active behavior at a trial-by-trial level. This makes it likely that the changes we observed in IT informed the animal's trained behavior.

(3) **Training curves:** This task had a substantial perceptual learning component, illustrated by the monkey's slow, gradual improvement, indicative of a trial-and-error learning strategy (cf. Fig S2 mentioned in point 1.1). This is notable since the subjects were already fully acquainted with the rules and structure of the task. This can be seen empirically in the number of trials they unsuccessfully completed (Fig S2C), demonstrating that they knew how to initiate and complete a trial. Of course, theoretically, based on this behavioral data alone, we do not know whether these improvements were achieved via changes to the readout alone. Yet, the slow and gradual nature of the behavioral changes makes changes in encoding more likely given prior theoretical work (e.g., McClelland, McNaughton, & Randall, 1995; Russin, Pavlik & Frank, 2025).

We have added a paragraph to the discussion to distill these insights and to suggest follow-up experiments:

“Several of our observations suggest that the encoding changes in the IT cortex were functionally relevant for the animals' trained behavior. First, training specifically increased linearly decodable information for trained objects compared to novel objects (Fig S8), arguing against training-nonspecific, epiphenomenal changes. Second, trained IT responses became more consistent with the animals'

behavioral choices at an image-by-image level during passive viewing (Fig S14B), suggesting that the changes in IT could also shape behavioral decisions. Third, the slow, gradual nature of the behavioral learning curves (Fig S2)—which occurred despite subjects already being fully acquainted with the task aligns with theoretical accounts linking slow learning dynamics to changes in cortical encoding rather than rapid rule learning (e.g., McClelland, McNaughton, & Randall, 1995). This account predicts that the encoding changes we observed should be sensitive to differences in training curricula and show limited generalization to new scenarios (Russin, Pavlik & Frank, 2025). ”

Discussion - Adopting a systems-level perspective clarifies IT's role in object learning

1.4. There's a lot of references to anatomically mapped, but I really don't know what that means. Off hand I can think huge number of anatomical features (feedback, neuromodulation, laminar connectivity, neuronal activity correlations, lateral connections) that seem absent here. What does a non-anatomical layered convolution model look like and how does it differ in performance? Line 652 makes reference to biological constraints: what constraints and do you do any tests in which those constraints were absent?

We appreciate the reviewer's observation that the list of anatomical features that may matter to this research is extensive. In this project, the term ‘anatomically-mapped’ means that we ensured that our chosen layer in a given base model was the best possible reflection of task-naïve IT responses in any (pretrained) base model. We are thus fixing our choice of which model layer to analyse after training by relying on an encoding analysis of our target area IT (Fig S9). Thereby, we established an anatomical link between a layer in the network and a brain area. A secondary commitment we made was about how many units in our model are equivalent to a single IT recording site (Fig S10). Here, again, we put the recording sites, sampling the IT region, and the units in the networks on an equal footing by matching their information. That is, given that a single recording site picks up signals from thousands of neurons, how many units in a network are necessary to approximate this information? While a one-to-one mapping between recording site and layer unit may seem intuitive, given the differences in the number of units we encountered in the IT-mapped layers, this approach was not tenable. An analysis performed for this review highlights that these choices matter for accounting for the observed training-related differences in IT (cf. point 1.7 for a control for IT-to-layer mapping control).

We fully agree with the reviewer that there is potential to further extend this anatomical mapping, e.g., by modeling different subcircuits and their respective excitatory and inhibitory connections. We now discuss this as an avenue for future research:

“Moreover, a promising avenue for refining our understanding of how the observed differences in IT are implemented is to refine the spatial and functional scale of the mapping between IT recordings and ANN activations, e.g., by mapping out correspondences to excitatory and inhibitory neurons (e.g., Sanghavi & Kar, 2023).”

Discussion - Limitations and Future Directions

Further unease with regard to mapping artificial neural networks to real ones comes the completely non-biological nature of gradient descent, [...]

We agree that gradient descent is not a biologically realistic learning rule, and we do not interpret its use as a claim about how synaptic plasticity is implemented in IT. In this work, gradient descent serves as a mathematical tool describing how behavioral change can be achieved by adapting hierarchical representations. It provides a normative reference point for understanding the direction and magnitude of representational changes required to support learning. Gradient descent is the optimal way to solve this kind of problem, and many biologically plausible algorithms adopt gradient descent as their gold standard (Bredenberg & Savin, 2023; Richards & Kording, 2023). Given the reviewer's comment, we highlight below a part of our Discussion where clarify that we do not think that our work can speak to any implementation details of how gradient descent-like solutions may be achieved by the brain, but rather, given the resolution and limitations of our data, we see that an algorithm such as gradient descent is a good computational approximation of the state changes we observed in IT.

"Our success in modeling IT plasticity using gradient descent raises important questions about the relationship between artificial and biological learning. While gradient descent may not mirror biological learning mechanisms, our results suggest it captures fundamental principles about how hierarchical networks optimize behavior. Our network simulations suggest that a model of IT's plasticity is readily obtained by combining SMART models with task optimization via gradient descent to mimic object training. We here viewed gradient descent as an effective tool to simulate a normative perspective on the observed neural changes. In other words, if one wants to understand how a distributed system should be adapted in service of task performance, gradient descent provides the optimal solution to achieve this (Bredenberg & Savin, 2023; Richards & Kording, 2023). From this perspective, the success of our modeling approach may not be surprising. Viewing our results through this lens, we found that IT plasticity appears to follow principles of efficient task optimization, where changes in sensory representations cannot be understood in isolation from behavioral demands. The magnitude and distribution of observed plasticity reflect both computational constraints and network architecture. However, the relationship between gradient-based learning in artificial networks and plasticity in biological circuits requires careful consideration. There remains a possibility that the changes achieved via gradient descent, even though they are optimal, are not achievable in biological brains. While gradient descent computes optimal weight updates using non-local information propagated backward through the network, biological circuits face more severe constraints (Lillicrap et al., 2020; Song et al., 2020). Neural plasticity likely operates with more constrained mechanisms than artificial networks, such as predominantly local information at synapses (Sacramento et al., 2018), temporally sparse and delayed feedback signals (Guerguiev et al., 2017), and circuit motifs that may restrict information flow (Lillicrap et al., 2020; Richards & Lillicrap, 2019). For example, the structure of neural circuits could limit how task-optimizing error updates may be approximated and propagated throughout the system, affecting the distribution and magnitude of changes in IT. Remarkably, our results suggest these biological constraints may not critically impact the pattern of plasticity at the scale of IT responses and behavior. We found that multiple learning algorithms, operating under different constraints, could produce changes matching empirical observations. This implies that the

observed IT plasticity may reflect general principles of hierarchical learning rather than specific implementation details. Nevertheless, it is conceivable that these factors will become important when modeling inter-areal coordination, learning dynamics over time, individual differences in learning trajectories, and the transfer of learning to novel tasks. Future work combining our normative modeling approach with more biologically detailed implementations may help bridge the gap between artificial and biological learning mechanisms."

Discussion - Gradient Descent as a Window into Neural Plasticity

[...] and the fact the feedback that actually matches the training (binary) is not better than the other feedbacks (and in fact looks fundamentally different in Fig S7).

The reviewer is correct that binary feedback models showed different patterns (Fig S15). We believe this reflects a limitation in how we implemented binary feedback rather than evidence against our framework, as we explain below:

The key difference between feedback types concerns distractor information. For the step-wise/standard training, the label contained information about all seven distractor classes and the target class, whereas in the binary case, it only contained information about one randomly chosen distractor class in addition to the target class. The information about the distractor classes was thus sparser, and we hypothesized that this may be a better model for the feedback received by the monkey. While binary feedback also produced performant models (although to a lesser extent, Fig S12A), such models tended to be the least effective at mimicking IT (Fig. S12B). They also appeared to be markedly different in their image-by-image error patterns (Fig S15). This latter result in particular suggests that distractor-wide feedback was necessary to produce monkey-like error patterns.

We hypothesize that this difference arises because we modeled only sensory learning, omitting task-structure reasoning that monkeys might additionally employ. For example, consider that a subject has a good understanding of the structure of the task: This means that they know that there can only be one correct choice/object and that there exists a set of distractors, but only one distractor is shown on a given trial. Using this structural knowledge, a subject can infer that a positive reward means that they've made the right choice and that, hence, all other distractors, even those that were currently not present, would be incorrect. Conversely, making a mistake enables them to know that they should have picked the other object and that any other object (including those not present in the current trial) would be incorrect. Monkeys are capable of such abstraction and may leverage it during learning. However, our binary feedback implementation did not include this structural inference step. Rather, binary simply applied gradients to the target and one randomly selected distractor per trial, and was thus constrained to changes in sensory representations.

1.5. More data is not particularly helpful if it doesn't give rise to insights, and here testing 660 models and finding roughly 10% meet the requirement, with very little discussion about factors or differences that are critical (or even related) to explain the physiological changes, seems to fall into this category. If I want to make a realistic model of IT, how should I proceed? Or is the point that it largely doesn't matter what I do because a large number of models work? If I run 10,000 models

and get a 1000 to "work" by satisfying the 3 requirements, what have I learned? Similarly how useful is a mean model (Fig 5D)? If I varied the hyperparameter space differently wouldn't I get different distributions? And if a particular model (say one in the 0/3 category) can explain the physiology, how can that be ruled out? (Maybe IT is like that model with a set of hyperparameters that is governed by physiological/biological constraints).

We agree with the reviewer's concern and performed additional analyses to better contextualize our results. We see our work as a proof of principle that there exist ANN analogs to the differences observed in IT.

To clarify, our model simulations (and hyperparameters) served to identify a varied set of models that could mimic the monkey's performance. Focusing on matched behavior, we relinquished control over the hyperparameters and their interpretability. We did not attempt to interpret individual hyperparameters in isolation, as their effects are inseparable from the model's pre-training state, architecture, and random initialization. In this sense, hyperparameters do not constitute a low-dimensional explanatory axis that can be meaningfully compared across models. Instead, our analysis reveals a solution landscape shaped jointly by initial representational structure, learning dynamics, and regularization, within which IT-like outcomes emerge. Additionally, whether the choice of hyperparameters yields an IT-like model is likely intricately linked with the naïve state of the base model. Therefore, interpreting hyperparameters across different (pretrained) base models may not be possible or insightful. Rather, our work illustrates that there are "many roads to Rome": there is a landscape defined by a model's initial naïve state, the random initialization of the output layer, the learning rate, and regularization strength. Specific location(s) within that landscape correspond to IT-like solutions. That being said, we agree that there is still a need to understand what makes some of these training differences more IT-like than others. In our reply to the following question (point 1.6), we propose an additional perspective (Fig S13) on how the manifold in each of these networks changes as a function of their IT-likeness. We believe that such a level of analysis is more appropriate for distilling commonalities across trained models and hyperparameters.

1.6. In a related matter, while the 3 requirements makes a nice story, it would appear that the strongest constraint is that of changes in linear decoding performance (which get us from 660 to 75). What's going on here? Is there a reason why that would be true? And although it's a small number, how can improvements in linear decoding not be associated with the other 2 factors? The graphs of Fig 5 implicitly assume that all of the 3 factors are equivalent (the 3 1/3s are all the same, and 3 2/3s are all the same). Is that true and if not, how can they be thrown together? There's a potentially interesting story here (that parallels previous ones) about invariance organically being generated as a consequence of object training irrespective of the nuances of the model or training, but if that's the goal, a lot of these questions need to be cleared up.

We thank the reviewer for highlighting this issue. The linear decoding criterion indeed is the most selective, reducing the model set most strongly. Indeed, we observed that these metrics are highly positively correlated with one another (see figure below, selectivity vs. representational strength: $r = 0.657$, $p < 0.0001$, CI (95%) = [0.57, 0.73]; selectivity vs. decodability: $r = 0.714$, $p < .0001$, CI (95%) =

[0.64, 0.77]; decodability vs. representational strength: r = 0.691, p <.0001, CI (95%) = [0.61, 0.76]). However, we do not view this as indicating that the other two metrics are redundant or secondary. Rather, this reflects how these three metrics capture complementary but correlated aspects of the same underlying representational change. We chose to use them as a set since all of them provide a slightly different take on the neural changes, and we expect that, ultimately, a good model of IT should satisfy all of these requirements.

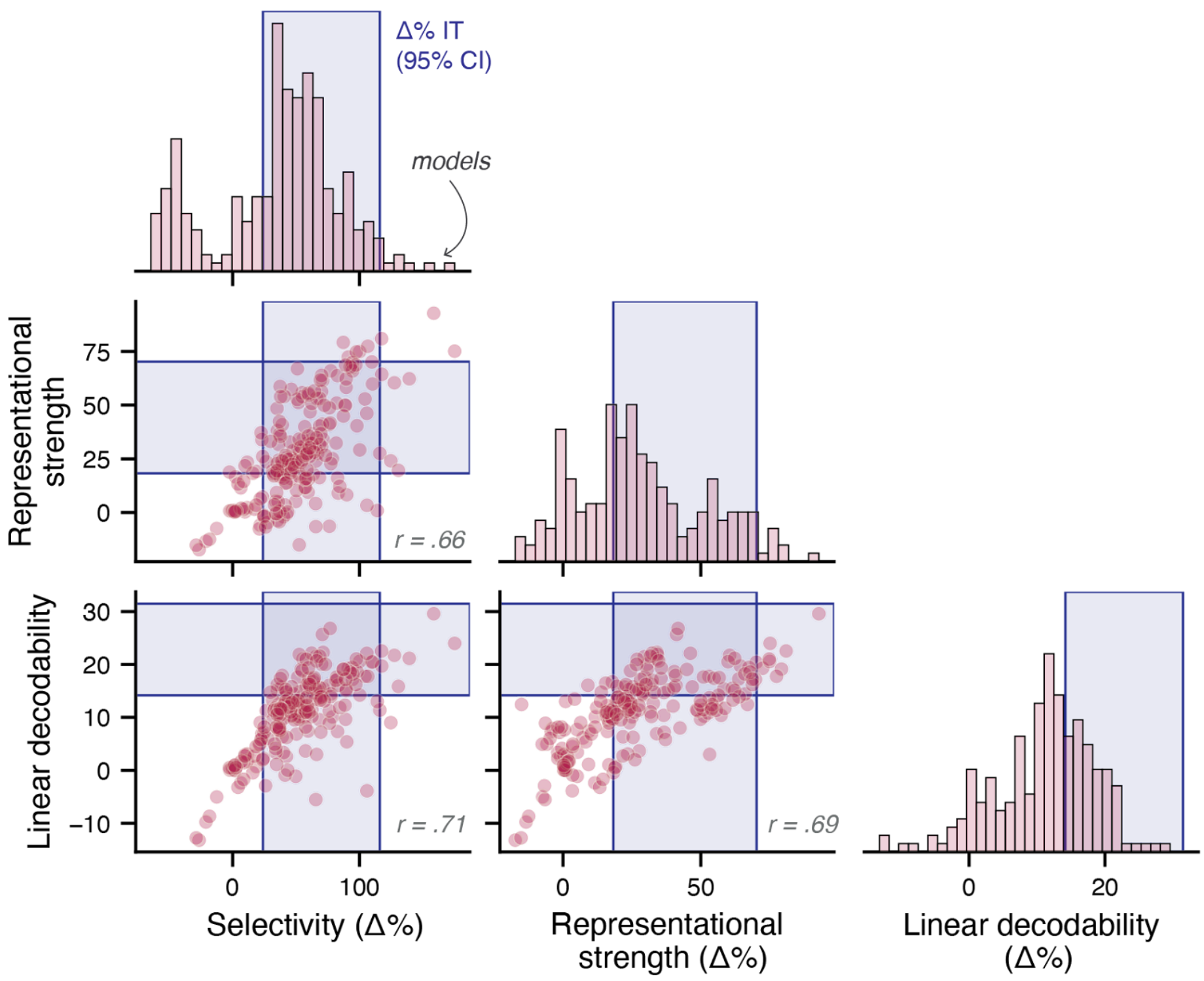

Figure R2. IT metrics are highly correlated. The diagonal histograms show the number of models displaying a particular degree of change. This data is replotted from Fig 5C. The blue shaded areas show the 95% CI of the difference of these metrics in IT in all plots. The off-diagonal panels show how these metrics relate to one another. Each point shows a single model, and the blue shaded areas mark the observed range in IT for each respective metric.

Indeed, linear decoding is the most selective constraint. However, as shown in the first figure above, it alone is insufficient: For example, some models with the highest increase in linear decoding show excessive changes in both selectivity and representational strength, making them IT-unlike. This illustrates that we're looking for a system that increases the linearly decodable information without relying on excessive changes at the level of single sites. Thereby, the three metrics provide different characterizations of the underlying phenomenon. Collectively, these metrics suggest that we're looking for a type of change that effectively increases the information about task-related information, in a fairly distributed manner.

We further investigated what might set IT-like models apart by performing additional geometric analyses on the changes to the manifolds of these models over training (following recent metrics proposed by Cheng et al., 2025). Specifically, we teased apart contributions of four distinct mechanisms (signal enhancement, manifold shrinkage, signal rotation, and manifold warping), all capable of increasing

information about the trained objects via distinct changes to the manifolds. This analysis revealed that manifold shrinkage was the most differentiating aspect for IT-likeness. Although all metrics showed differences with the IT-unlike groups, the remaining three mechanisms did not distinguish between different levels of IT-likeness. Manifold shrinkage describes the relative changes of how tightly the manifold is formed by the images (i.e., how compact the manifold is). For IT-like models, we see that the manifold variance remains roughly stable in naïve and trained networks, whereas for less IT-like groups, variance increased with training (i.e., images were forming larger clusters than before training). This provides a complementary perspective on our previous results: it shows that an increased signal was insufficient to mimic IT metrics, but rather a reduction in variance (across images from the same object) was symptomatic. This resonates with our observations regarding the link between the metrics: Likely, excessive increases in selectivity would result in larger manifolds.

We now include this analysis in the results and mention it in the discussion:

“To further probe what distinguishes IT-like models from the alternatives, we conducted geometric analyses of the representational manifolds before and after task training (Fig S13). Following metrics proposed by Cheng et al. (2025), we quantified changes in four distinct geometric properties that can each contribute to increased task-relevant information: (1) signal enhancement (increasing distance between target and non-target manifold centroids); (2) manifold shrinkage (tightening the clustering of images within each object category); (3) signal rotation (repositioning the manifold centroids relative to each other); and (4) manifold warping (altering the covariance structure across dimensions, Fig S13A). We compared these metrics across models grouped by their degree of IT-likeness (i.e., the number of IT metrics matched). While all four metrics qualitatively distinguished IT-unlike models (matching 0 metrics) from IT-like models (matching all 3 metrics), manifold shrinkage showed the most systematic relationship with IT-likeness, revealing graded differences across intermediate levels (Fig S13B). Specifically, IT-like models maintained relatively stable manifold variance between their naïve and trained states, whereas models matching fewer IT metrics exhibited increased variance after training, indicating that images formed larger, more dispersed clusters following task training. This finding provides a complementary perspective on our earlier results: increases in neural selectivity alone are insufficient to reproduce IT-like changes; rather, training must enhance discriminability via subtle and distributed changes across the population.”

Results - Task-optimized ANNs reproduce IT's task-training-induced differences

“We started to tackle this question by performing a geometric manifold analysis, asking whether there are any properties that set apart IT-like models from less IT-like ones. Interestingly, we found that models that maintained the size of their object manifolds over training were the most similar to IT. This finding is in accordance with another study on perceptual learning that also identified reductions in manifold size as the key driver for improving information during learning (Cheng et al., 2025). This speaks to the importance of coordinated, subtle, and distributed changes within biological systems and learning principles that prioritize invariance within classes over a separation between classes.”

Discussion - Understanding What Makes Models Match IT Plasticity

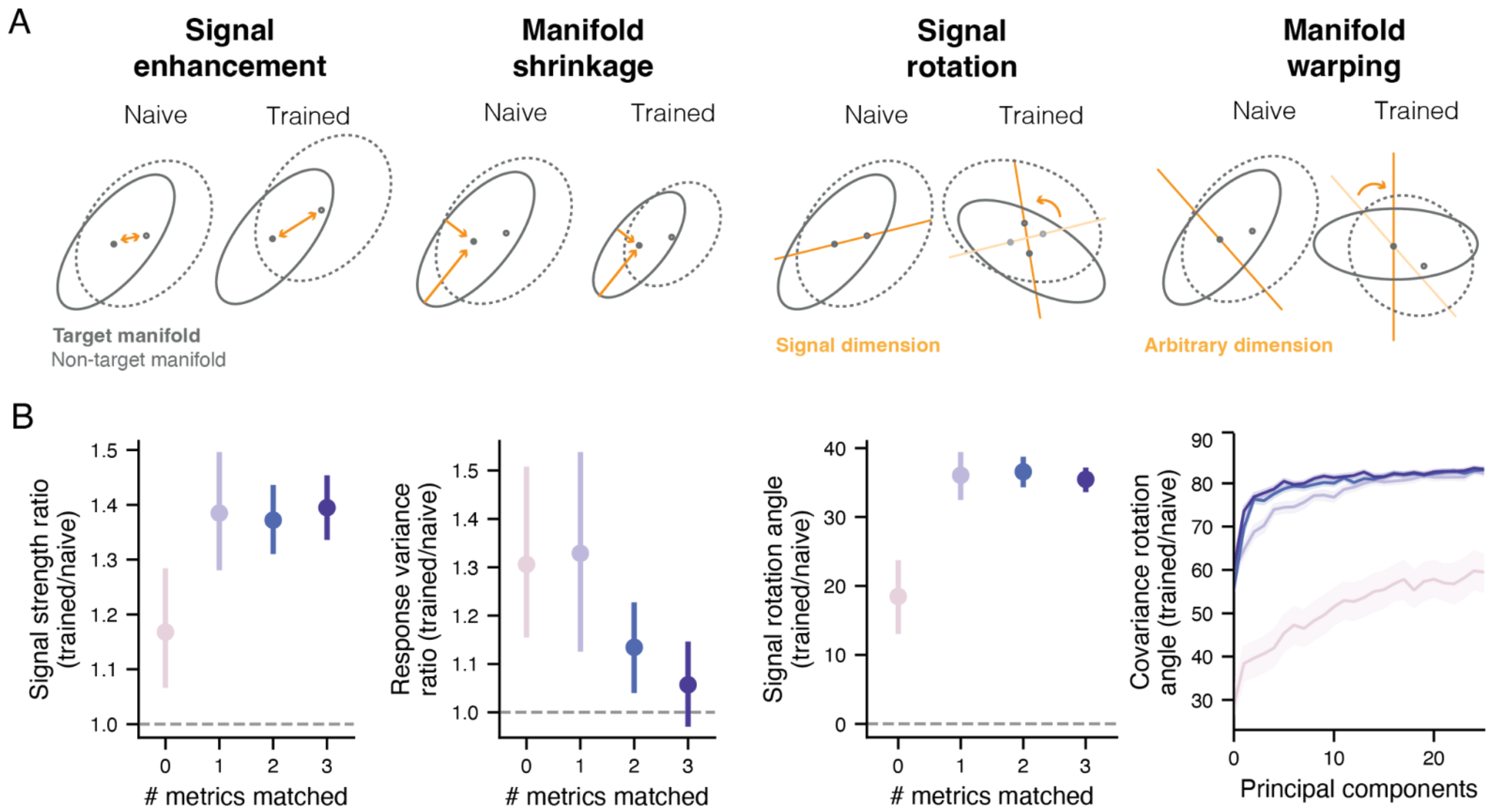

Figure S13. Identifying geometric properties that distinguish IT-like models. (A) Schematic illustration of learning mechanisms on object manifolds. To understand what distinguishes models that match IT plasticity patterns from those that do not, we analyzed different geometric factors driving manifold changes during training using metrics from Cheng et al. (2025). Training can increase task-specific information through four mechanisms: (1) Signal enhancement (SE): increasing the distance between target and non-target manifold centroids; (2) Manifold shrinkage (MS): reducing manifold size, thereby tightening clusters of instances from the same object; (3) Signal rotation (SR): rotating manifolds to change the relative orientation of centroids; (4) Manifold warping (MW): altering the covariance structure across dimensions. Solid ellipses represent target object manifolds; dashed ellipses represent non-target manifolds (incl. all other objects). (B) Comparison of geometric metrics across models grouped by IT alignment (x-axis: number of IT metrics matched, from 0 to 3). All four metrics distinguish models matching no IT metrics from those matching some or all. SE, SR, and MW primarily separate IT-unlike from IT-like models, showing relatively binary patterns. MS reveals a more graded relationship: IT-like models maintain approximately constant manifold variance across training (MS ratio ≈ 1), whereas less IT-like models show increased manifold size. Error bars show the CI (95%) of the mean estimates across models.

7. One important control that is absent in the computational models is any test for layer specificity. That might be difficult given the metric applied for measuring "IT-ness" in naive models is often very broadly distributed (Fig S5). But in combination with the large number of models and feedback that seem capable of explaining physiology it brings up the uncomfortable point that similar results (in terms of change in performance as seen in Figure 5) might be found across a huge number of layers. In this case there would be even less of an ability to associate the artificial NNs with biological NNs and for that matter NN changes with behavior.

We agree with the reviewer that assessing the extent to which the effects identified are specific to the IT-mapped layer is essential. As correctly pointed out, the quality of IT encoding across layers is distributed across the model. In our analyses, we simply chose the best observed IT encoding score (Fig S9, formerly Fig S5). To address how dependent our results are on this choice, we repeated our analysis for two additional, less IT-like layers. Specifically, we chose an earlier and a later layer (before and after the IT-mapped layer, respectively) that both had a comparable IT encoding score (Fig S11A). These simulations revealed that only the IT-like layer produced a reliable overlap with the empirical data (Fig S11B). For selectivity, we observed that the earlier layer did not increase sufficiently in selectivity, whereas the later layer increased its selectivity too excessively (in comparison to IT). For representational strength, control, and IT-like layers showed similar patterns. For decodability, neither the early nor the late control layer increased its information to the same extent as IT.

We now mention this additional control analysis in the results section:

“To test whether the observed changes are specific to the features of IT-mapped layers, we performed a control analysis where we replaced the IT-like ANN layer with early and late control layers with less IT-like encoding. For selectivity and decodability, and less so for representational strength, we observed that IT-like layers most aptly captured the neural differences (Fig S11).”

Results - Task-optimized ANNs reproduce IT's task-training-induced differences

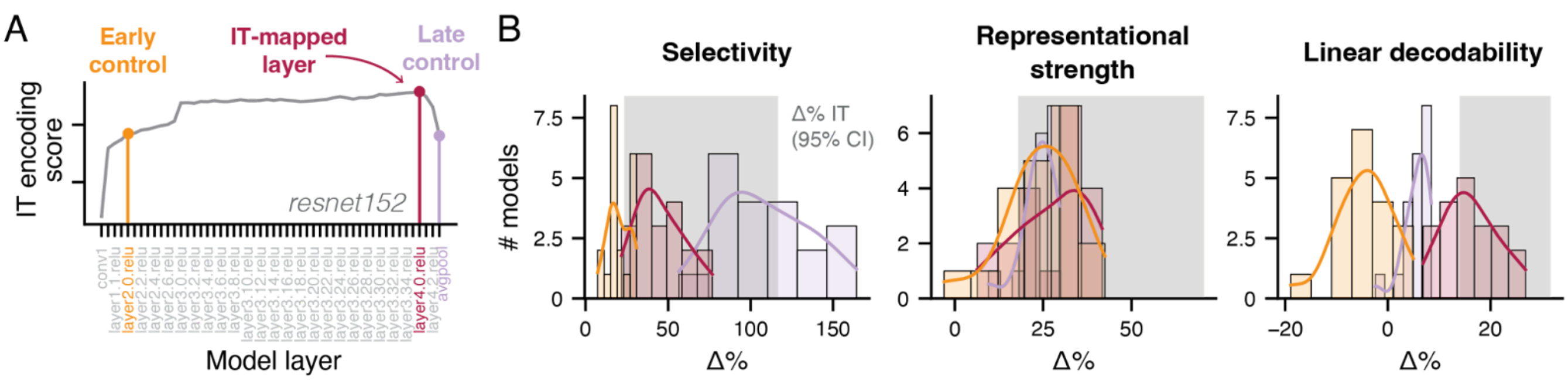

Figure S11. IT-like layers best capture neural IT differences. (A) For our main analysis, we selected the model layer showing the best encoding of naïve IT responses. To validate this approach, we compared this IT-like layer to two control layers for one base model (resnet152): (1) a late control layer, the next deeper layer after the IT-mapped layer, which showed the worst IT encoding score, and (2) an early control layer, an earlier layer matched to the late control in IT encoding score. All comparisons used the same 20 trained model instances of resnet152. (B) Whereas model changes from the IT-like layer (red histograms) fell mostly within the observed IT changes, model changes from the control layers (orange and lilac histograms) tended to fall outside of the empirically observed differences.

Reviewer #2 (Remarks to the Author):

Overall, the authors combined carefully controlled cohort design, rigorous statistical procedures, and computational modeling to make a strong case for moderate IT plasticity. While their approach convincingly addresses the central question, it also highlights the need to explore how downstream circuits contribute to the remaining behavioral gains.

We thank the reviewer for their positive evaluation of our paper. We are delighted to hear that they deem our results convincing.

I have a couple of minor methodological questions that don't undermine the core findings or preclude publication:

2.1. The authors focus on the early, feed-forward response (70–170 ms) to maximize signal-to-noise but don't explain why they did not analyze later post-stimulus epochs. Task-modulated, feedback-driven effects—and true category-related signals in IT—often emerge ≥ 300 ms after onset of the stimulus (e.g., Meyers et al., 2008; McKee et al., 2014). By restricting to this early window, the study may underestimate the full magnitude of learning-induced plasticity and miss late-emerging, abstract category dynamics. It would strengthen the manuscript to clarify why later windows were not examined.

We thank the reviewer for allowing us to clarify this analysis choice. Our focus on the 70–170 ms post-stimulus window was motivated by both theoretical considerations (based on prior studies) and constraints of our stimulus presentation paradigm.

First, our experiment employed a rapid serial visual presentation (RSVP) design in which each image was shown for **100 ms**, followed by a **100 ms blank interval**, after which the next image appeared. Although images were randomly interleaved, neural activity beyond ~200 ms after stimulus onset inevitably overlaps with responses evoked by the subsequent stimulus. As a result, later post-stimulus epochs cannot be unambiguously attributed to the currently presented image, making interpretation of learning- or image-specific effects in those windows difficult. Restricting analyses to the 70–170 ms window allowed us to isolate the neural response that is most reliably stimulus-locked and uncontaminated by subsequent inputs.

Second, averaged firing rates in task-naïve IT during this window show a reliable, linear relationship with object recognition behavior in primates (Majaj, Hong et al., 2015). Due to the close link to behavior, we hypothesized that this part of the IT response should also be modified upon task training. Furthermore, this time window has been successfully modeled using ANNs (e.g., Yamins et al., 2014), and we could thus rely on a robust mapping in the task-naïve setting. This was a critical precondition for our study, since the (task-naïve) baseline contextualizes any shared training-induced differences. We now state this more explicitly in our methods summary:

"We chose this time window building on prior work showing that responses in this window feature a linear link with choice behavior (Majaj et al., 2015) and that these responses can be reliably predicted by ANNs (e.g., Yamins et al., 2014)."

Methods - Large-scale multi-electrode recordings in the inferior temporal cortex - Electrophysiological recordings

Our current results serve as a basis for expanding our approach to later time windows (>170ms) as suggested by the reviewer. We fully agree that the observable training-induced effects are likely even more pronounced than reported previously (Meyers et al., 2008; McKee et al., 2014). We noticed that these studies chose to present their images for much longer than our study did (600ms vs. 100ms). Longer presentation durations might partly account for these more pronounced later effects. It is an important avenue for future work to quantify the magnitude of these early vs. late training-induced differences and to understand the opportunities and limitations of current computational models in accounting for these. We have added this point to our discussion:

“It remains an open question how stimulus duration and analysis window affect the magnitude of training-induced changes: Indeed, prior work suggests even more pronounced effects during later windows, evoked by longer image presentations (Meyers et al., 2008; McKee et al., 2014).”

Discussion - Limitations and Future Directions

2.2. The choice to record under passive fixation—intended to equate sensory conditions across trained and naïve groups and avoid confounds from attention, decision-making, or motor planning—is understandable, and the authors rightly concede that comparing separate cohorts prevents tracking how IT representations evolve within the same individuals. Nevertheless, they should justify why they opted for two different groups instead of within-subject, longitudinal recordings, and discuss the trade-offs and practical considerations that precluded a same-subject design. Furthermore, the approach of relying solely on passive viewing may underrepresent the full extent of learning-induced plasticity, since category-selective responses in IT have also be found weaker under passive fixation than during active discrimination (see for instance McKee et al. (2014)).

We acknowledge that the choice of a between-subject design for studying learning deserves extensive justification. As already alluded to by the reviewer, this choice was partly motivated by the current methodological limitations of longitudinal recordings. Indeed, it remains a methodological challenge to track large populations of neurons in the same individual while keeping the data size and quality constant. By contrast, leveraging multiple cohorts of individuals allowed us to assemble an unusually large dataset for macaque electrophysiology (N=6). Assembling such a large dataset enabled us to demonstrate how training-related effects scale as a function of population size and anatomical location (cf. Fig 3). Such an analysis would be difficult to achieve with longitudinal recordings in individual animals.

Additionally, the choice of comparing training-induced differences between groups was motivated by a theoretical assumption: We sought to model differences that are shared between individuals and thus transcend any individual's idiosyncrasies. Current computational models of task-naïve IT do not yet have the precision to model interindividual variability. Pooling data across animals, therefore, provided a

well-defined and robust modeling target, enabling us to characterize learning-related changes that generalize across subjects and can be meaningfully compared to model predictions. We emphasize both of these points in the manuscript:

“While our study provides insights into how object learning shapes IT responses, several important limitations suggest promising directions for future research. A central limitation is our reliance on between-subject comparisons rather than tracking learning-induced changes within individual subjects. Though a within-subject design would be ideal for understanding how category learning modifies neural circuits, two key challenges justified our between-subject approach. First, characterizing learning-induced plasticity within individuals requires precise measurement of baseline states and careful separation of true plasticity from measurement variability. Current techniques for longitudinal neural recordings face significant challenges in maintaining stable measurements across extended periods, making it difficult to definitively attribute response changes to learning rather than recording instability or sampling differences (Chestek et al., 2011; Dickey et al., 2009). While recent advances in chronic recording methods and statistical frameworks for analyzing individual variability (Oby et al., 2019) show promise, the field still lacks robust approaches for tracking fine-grained neural changes over learning timescales. Second, our between-subject design enabled the collection of a larger dataset (six subjects) with better statistical power than would be possible in a within-subject study given current technological constraints. This approach revealed consistent training-induced changes across subjects, providing strong evidence for systematic plasticity in IT during category learning shared across subjects. Nevertheless, future studies combining improved longitudinal recording methods with our computational framework could reveal important insights about individual learning trajectories that our between-subject design could not address.”

Discussion - Limitations and Future Directions

Finally, we agree with the reviewer that training-related differences might be even more pronounced in an active task setting. Yet, as the reviewer notes, such active task effects are challenging to disentangle from other processes (e.g., attention, decision-making, motor responses). Importantly, observing reliable training-related differences even under passive fixation suggests that learning induces durable changes in IT representations that persist outside the task context. We therefore view our results as providing a conservative, lower-bound estimate of learning-induced plasticity. Quantifying how these effects are amplified or reshaped during active task engagement, particularly at later latencies, remains an important direction for future work. However, to illustrate that the observed changes are behaviorally relevant, we performed an additional analysis comparing an individual trial-specific IT response to behavioral reports on the same trials for two subjects in our task-trained group. We describe this analysis in-depth in response to Reviewer 1, point 1.1.

Reviewer #3 (Remarks to the Author):

This paper proposes a computational framework to model the effects of visual categorization learning on ventral visual stream responses. It combines an analysis of multi-unit recordings in the inferior temporal (IT) cortex of two groups of monkeys, one group of 3 monkeys trained in an object categorization task and another group of 3 untrained monkeys, and computational modeling. Extending previous studies of visual learning in macaque IT, they show an increased object selectivity in the trained compared to the untrained monkeys. This increased selectivity is, although statistically significant, small compared to the behavioral improvement, which aligns with previous studies. Then, and this I found the most interesting part, they trained, using the same images as in the electrophysiology study, a set of deep artificial neural nets (ANNs) and compared the training-induced changes of these ANNs to those observed in IT. They found that some, but not all, ANNs modeled the IT changes. These "IT-like" ANNs also modeled a training-induced increased selectivity for (some) stimulus transformations that are orthogonal to object category (which is a new finding by itself), and changes in the consistency of behavioral and IT-response-based object classifications.

I enjoyed reading this very well-written paper. The main analyses are thorough and carefully conducted, and they are further supported by appropriate control analyses (e.g., region-specific analyses in PIT, CIT, and AIT). Overall, the neural data extend and agree with previous studies showing increased selectivity in ventral visual stream areas V4 and PIT with visual perceptual learning and some studies in macaque IT showing changes in selectivity with shape or categorization learning (e.g., the recent study by Pearl et al., J Neurosci, 2024; also using Utah arrays for recording and decoding as analysis). The main advance offered by the present study is the meticulous computational modeling of the training effects seen in IT. That said, I have some issues and questions concerning the technical aspects of the methods/design and the data interpretation of this interesting study that need to be addressed.

We thank the reviewer for their enthusiastic evaluation of our work. Below, we set out to address the raised issues:

Main comments.

3.1. A general comment to start: the authors perform several control analyses, of which the outcomes are consistent with the hypothesis that the differences in neural selectivity between trained and naive subjects are induced by training instead of due to individual differences, electrode locations, or anatomical differences. However, between-monkey designs to assess learning effects, especially with a small number of subjects and different electrode locations among subjects, are tricky, especially without proper controls. For instance, it would have been great to have an untrained stimulus set presented in both groups during the fixation task while measuring the responses and show that the changes seen after object learning were not present for this control set of images. This would have

strengthened the conclusion that the reported IT response differences between the trained and naive groups are due to training.

We fully agree that such a control set would strengthen our results. The reviewer's suggestion implies a clear prediction: <u>if training is the primary driver of the observed IT differences, then images depicting objects not used during training should not show the same group differences under passive fixation.</u>

Although such a control dataset was not available for the initial cohorts, we took the reviewer's suggestion seriously and collected additional neural recordings to address the issue. Prior to this review, we had recorded IT responses from the trained macaques to a novel image set (10 untrained objects) under passive fixation for a different study. This allowed us to test whether training-induced changes generalize beyond the trained stimulus domain. However, we lacked matched task-naïve baseline recordings for this novel image set. We have now collected these data from an additional naïve subject (monkey P).

The stimulus set contained 10 novel objects (not part of the training), each presented in 50 differently rendered views (example images in Fig S8A). We analysed the neural recordings in exactly the same fashion as for the other subjects (55 sites, 1000 draws). To enable comparison, we matched decoding difficulty between trained-object and novel-object datasets using the naïve recordings (Fig S8B). Our analysis revealed two key findings. First, we replicated our main results using this subject's new recordings for the trained objects: We could decode significantly more object-related information from the trained compared to the naïve subject's recordings (Fig S8C, right panel). Second, in contrast, there was no significant difference between the two groups when decoding the identity of the novel objects (Fig S8C, left panel). Thus, we find that object training selectively amplifies differences for the objects that were part of training, further supporting that the observed differences are due to task training.

We included this additional analysis as an additional supplementary figure and refer to it in the manuscript:

"To what extent are the observed training effects specific to images depicting the objects used during training? Repeating the decoding analysis using another image dataset displaying new objects (10 objects, 50 images each) and another task-naïve subject (monkey P), we see that objects not featured during training did not elicit significantly different decoding accuracies (1 subject per group, 55 sites, Difference (mean ± SD) = 0.176 ± 0.147, CIDifference (95%) = [-0.118, 0.474], p = 0.212, 12.47% increase, Fig SXC, left panel). In contrast, the dataset showing the trained objects to the same subjects did produce significant differences in such matched conditions (Difference (mean ± SD) = 0.693 ± 0.147, CIDifference (95%) = [0.411 - 0.976], p < 0.001, 48.3% increase, Fig SXC, right panel)."

Results - Task training enhances task-relevant information in IT

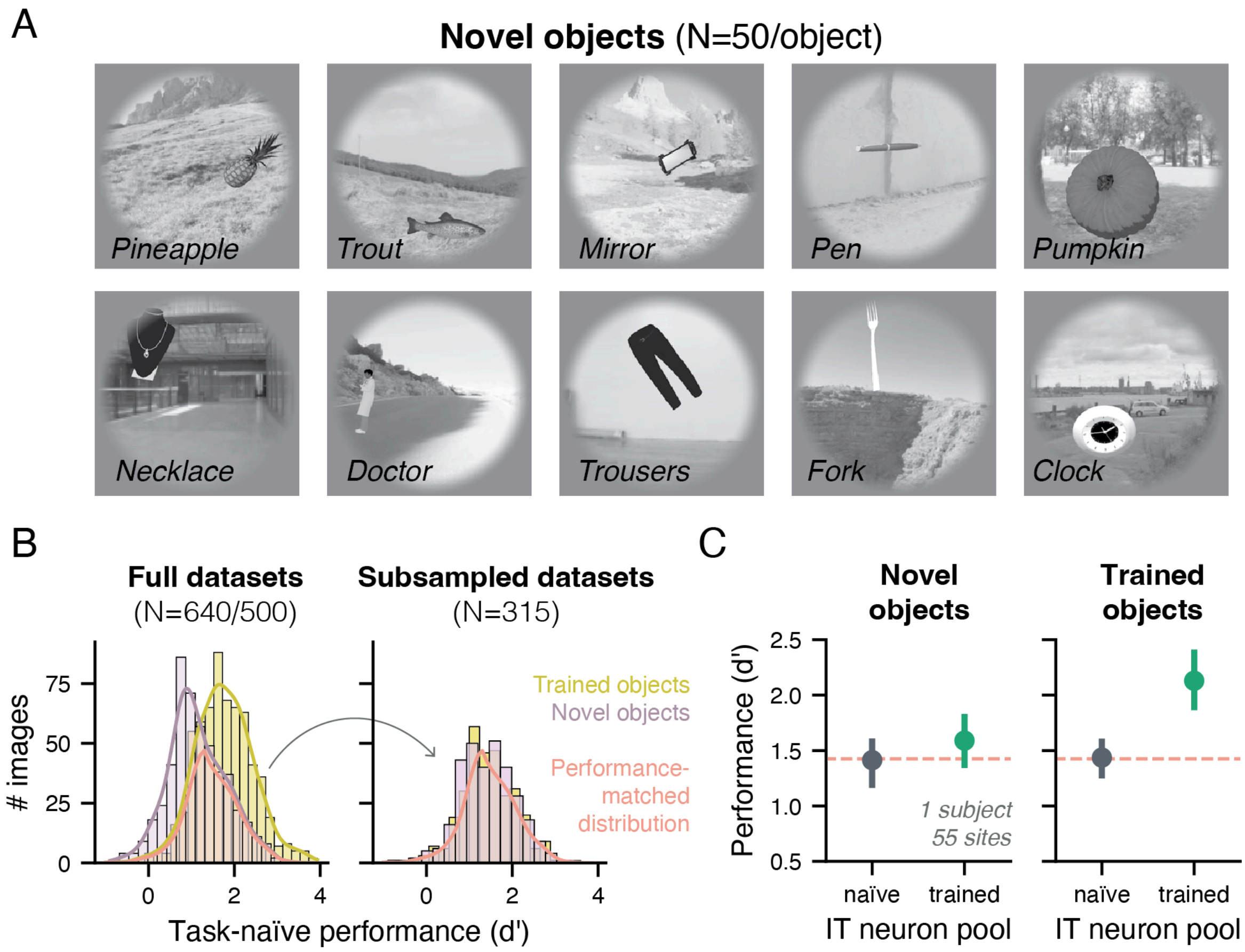

Figure S8. Task training does not increase decoding accuracy for novel objects. (A) To test whether training-related differences also affect the information about novel objects, we repeated our decoding analysis using a different image dataset consisting of 10 novel objects (50 views each) that were not part of the training objects. Since the original task-naïve subjects were not tested with these novel objects, we recorded data from a new task-naïve individual (monkey P; see Methods) for this comparison. For the task-trained condition, we therefore also selected one individual from the trained group and matched the reliability of both datasets based on our standard procedure (see Methods). (B) Controlling for dataset difficulty. To enable a comparison between datasets, we matched the difficulty of both stimulus sets based on the decoding accuracy in the naïve responses. This yielded reduced datasets (N=315 images for each stimulus set) matched in their naïve decoding performance and dataset size. (C) Decoding performance for trained versus novel objects. Even at the single-subject level, we observe improved decoding for the trained objects for the task-trained recordings. In contrast, we do not observe better decoding for the novel objects due to task training. Error bars show the 95% CI based on bootstrapping across neuron pools.

3.2. In their task, the monkey must segment the object from the complex scene background. Part of the training effects may reflect learning this object–background segmentation. It is possible that during the fixation task in which neuron responses were tested, the trained monkeys were segmenting the objects from the background more efficiently, because of the training, than the untrained monkeys, and this may have resulted in the observed changes in IT responses. This could explain why they find both an increased (invariant) object

decoding and an increased decoding of the category-orthogonal stimulus properties, such as of object position and size – better object segmentation in the trained group can result in both effects. This possibility should be discussed (they can relate their findings to the literature on the effects of learning on object segmentation).

We agree that object-background segmentation may be part of the computational problem solved during training. Our current analyses remain agnostic to the specific computations implemented by IT and the ANNs. Indeed, some recent work points to partially shared computational principles between the primate brains and ANNs in the domain of object-background segmentation (Seijdel et al., 2020; Seijdel et al., 2021; Loke et al., 2024; Djambazovska et al., 2024). Given the analysis presented in Fig S8 (novel object decoding, point 3.1), we speculate that at least a general (object-nonspecific) segmentation process cannot account for the observed benefits of training on IT decoding. We now discuss this possibility in the discussion:

"At the core of the computational problem solved by the brain and ANNs during object learning is that of invariance, the ability to identify objects over a large range of viewing conditions (DiCarlo, Zoccolan, & Rust, 2012). In our study, successful performance required invariance to an object's position, size, rotation, and, critically, its scene background. While invariance to intrinsic object properties is well-documented in the ventral stream (Brincat & Connor, 2004; Ito et al., 1995; Li et al., 2009; Rust & DiCarlo, 2010; Tovée et al., 1994; Logothetis et al., 1994; Logothetis et al., 1995; Op de Beeck & Vogels, 2000) and commonly encountered during natural viewing, our task imposed an additional demand: segmenting objects from highly variable backgrounds that, unlike natural viewing contexts (e.g., Davenport & Potter, 2004), provided no informative cues about object identity. Prior computational work suggests that ANNs implicitly learn object-scene segmentation during object recognition training, even on natural images (Seijdel et al., 2020), and that such segmentation strategies contribute to their ability to predict neural responses (Seijdel et al., 2021; Loke et al., 2024; Djambazovska et al., 2024). To what extent did this segmentation challenge contribute to the training-induced changes we observed? While our study was not designed with appropriate control conditions to directly test whether training improved general segmentation abilities, we performed a control analysis using novel objects (not part of the training set) displayed on backgrounds similar to those encountered during training. This analysis revealed no significant decoding benefit for these novel objects (Fig S8), suggesting that training did not yield general improvements in background-invariant representations. However, this does not rule out object-specific segmentation processes that may have contributed to improved discrimination of the trained objects. Future studies with explicit segmentation controls could further clarify to what extent segmentation demands shaped the distributed learning processes we have identified."

Discussion - Adopting a systems-level perspective clarifies IT's role in object learning

A related question is to what extent the general learning effects (changes in object and object-invariant selectivity) require explicit training or result from the extensive stimulus exposure during training? Would passive exposure to the trained stimulus set produce similar changes in IT? This can be discussed.

We acknowledge that our experiments cannot dissociate the contributions of task engagement versus stimulus exposure, as these were necessarily coupled during training. We now make this limitation and its implications explicit in the Discussion:

“Another crucial direction is understanding how learning unfolds across different timescales and its interplay with passive exposure via unsupervised learning (Jia et al., 2021; Li & DiCarlo, 2008, 2010; Li & Dicarlo, 2012; Zhong et al., 2024) as well as with pre-existing perceptual expertise (Collins & Behrmann, 2020; Martens et al., 2018; Tanaka & Taylor, 1991). Our study examined the end state of object training, but important questions remain about how representations evolve during learning. How do unsupervised learning, passive exposure, and existing perceptual expertise facilitate the observed differences in IT responses?”

Discussion - Limitations and Future Directions

3.3. Related to the previous comment: In the categorization task, the location, size, and rotation of the object are irrelevant to the task. Based on previous literature (e.g., Sigala and Logothetis; De Baene et al), I would have predicted little if any effect of training on IT selectivity along these irrelevant, category-orthogonal dimensions, which is not what they see (see main point 2 above). It is interesting to see that this was also not true for the ANNs – at least those that are IT-like. This counter-intuitive result deserves a comment/discussion.

We agree that our findings seem counterintuitive, given prior considerations suggesting learning should be specific to the discriminating features. According to this view, training on object identity should affect only identity representations while leaving task-irrelevant dimensions (e.g., position, size) unchanged. Our findings extend this prior work by demonstrating that learning affects multiple latents simultaneously. Based on our results, we propose that learning proceeds on a highly distributed representation that holds information about a variety of latents (such as location or object identity), all available for downstream readout. Crucially, while these latents can be decoded independently, they are not encoded independently. As a result of this joint embedding, a change to the encoding of object identity would also concurrently affect the encoding of unrelated latents, such as position, even when those dimensions are not explicitly task-relevant. This joint encoding account is consistent with prior evidence that object identity and spatial latents are jointly encoded within overlapping IT populations rather than in strictly separable subspaces (Hong et al., 2016). In addition, a recent study suggests that the optimization of spatial latents also improves the decoding of object identity and that either objective results in models with ventral-stream-like representations (Xie et al., 2024).

To address the reviewer’s point explicitly, we have added a dedicated paragraph to the Discussion clarifying why training-induced changes can emerge along task-irrelevant dimensions and how this reconciles our findings with prior literature:

“Another critical question is whether training-induced changes in IT's encoding are narrowly tuned to the specific distinction being reinforced (object identity) or whether they more broadly affect IT's representational geometry. Our findings suggest the latter: training not only changed the linear separability of the trained objects but also affected other visual latents represented within the same neural

populations, including spatial position and other identity-invariant features (Fig 5). This pattern aligns with prior work showing that IT simultaneously represents multiple visual latents (Hong et al., 2016) and recent evidence that training on different visual latents can yield convergent representations aligned with ventral-stream activity, partly because optimizing for one latent implicitly facilitates learning about others present in the world (Xie et al., 2024). Critically, ANNs trained through distributed optimization across the network hierarchy naturally reproduced these multi-faceted changes, demonstrating that ANNs are an appropriate testbed for capturing IT-like encoding and its distributed changes during training. This perspective enriches prior views of perceptual learning by showing that changes are not limited to the trained dimension but rather reshape hierarchical representations that simultaneously encode multiple visual dimensions, making them amenable to a variety of downstream readouts."

Discussion - Adopting a systems-level perspective clarifies IT's role in object learning

3.4. The reliability distributions for the trained and untrained neural data are very different, with lower reliabilities (many close to zero) for the trained sites. Any explanation for this difference? Although the authors matched reliabilities between the two groups of sites, this again raises the issue (see comment 1 above) of the possibility that differences unrelated to training can explain the "general" training effects (for relevant and irrelevant dimensions) seen in the present study. Also, a reliability criterion of 0.3 is pretty low. How robust are the training effects when changing (increasing) this reliability criterion?

We thank the reviewer for raising this important point. The apparent difference in reliability distributions between trained and task-naïve datasets does indeed require explanation, and it is critical to establish that our conclusions do not depend on reliability-related confounds.

The apparent difference in reliability distributions arises from differences in acquisition setups across the datasets. Specifically, for two of the three untrained monkeys, we were limited to recording 256 of the 288 available channels. These channels were selected based on early sampling to prioritize electrodes with more reliable signals, thereby biasing the untrained datasets toward higher-reliability sites. In contrast, for the trained animals, all 288 channels were recorded, regardless of their reliability, yielding a more representative distribution that naturally includes a larger proportion of low-reliability sites.

Importantly, however, we expect this acquisition-related bias to favor the untrained monkeys as their datasets are enriched for higher-reliability sites. Yet, even under stringent reliability matching between groups (see Fig S5), we find that the trained monkeys exhibit higher decoding accuracies. This demonstrates that our conclusions are robust and not driven by differences in reliability sampling.

We now mention this control analysis in the methods section and have included the figure below in the supplement:

"Additional control analyses demonstrate that the choice of reliability criterion did not qualitatively affect our main results (Fig S5)."

Methods - Neural and behavioral data analyses - Neural recording quality metrics and pooling

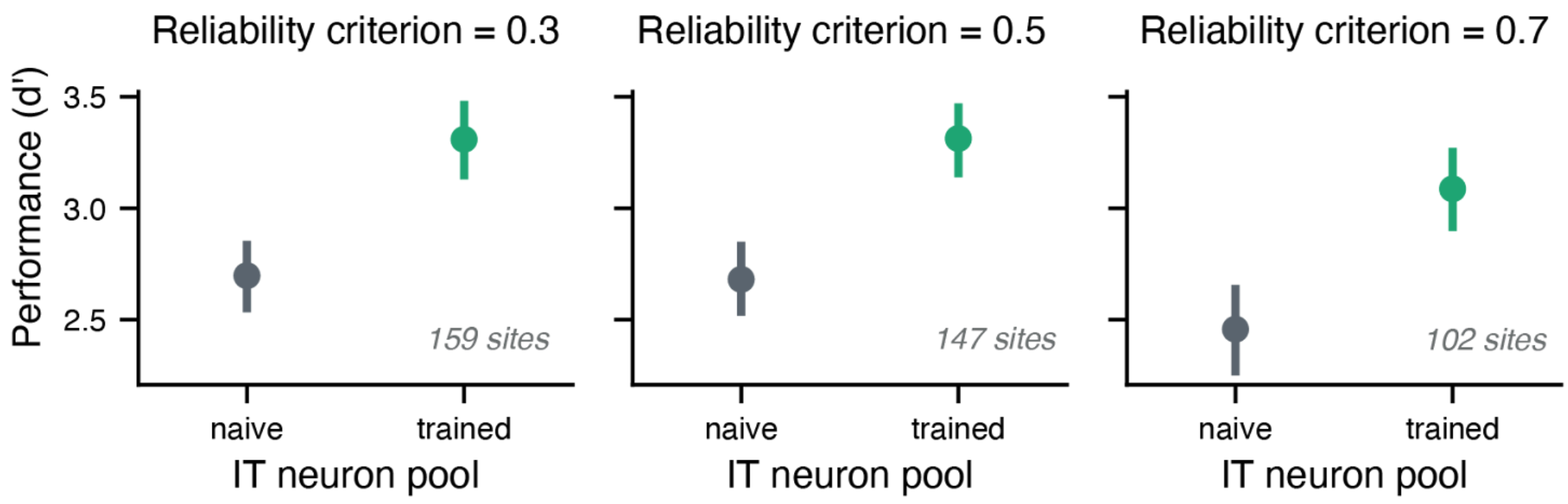

Figure S5. Training-induced decoding differences are not dependent on the choice of the reliability criterion. Neuron pools were sampled from recording sites passing a given reliability criterion. To test whether our results depend on this criterion choice, we repeated the analysis using different reliability thresholds. Although increasingly stringent reliability criteria (left to right) progressively reduced the number of available sites (annotated in grey), training-induced differences in decoding accuracy remained significant across all thresholds (all $p < 0.001$).

3.5. The authors examined several measures related to IT object selectivity, which is excellent. However, were there differences in overall (net or raw) firing rates between the two groups? Were responses stronger (or weaker because of familiarity with the objects) in the trained compared to the untrained groups? And if so, can this have impacted the selectivity changes (e.g., decoding)? Perceptual learning studies have reported decreased response variability (Fano factor) upon learning. Was this also the case here?

To test whether there are differences in firing rates between the naïve and trained IT recordings, we quantified the visual drive in both datasets by comparing the responses evoked by images (N=640) to those of blank screens (N=8). To standardize this difference, we compute a *d'* score as follows (based on Kar et al., 2019):

$$d'_{visual} = \frac{avg(rates_{images}) - avg(rates_{gray})}{\sqrt{0.5\,(\sigma^2_{rates\ images} + \sigma^2_{rates\ gray})}}$$

where we compute the mean difference per site between the evoked rates at 70-170ms across images, normalized by the standard deviation across images for each of the pools drawn via our bootstrapping procedure (see Methods). The mean and 95% CI across pools are plotted in the figure below for each group.

While we see a slightly elevated visual drive in the naïve vs. trained group ($mean_{naïve}$= 0.63, $mean_{trained}$= 0.54), this difference is not statistically significant (*p* = 0.142, $CI_{difference}$ (95%) = [-0.219, 0.019]). As a result, we do not think that this would have impacted our reported IT measures.

Similarly, the Fano factor showed no significant difference between groups. We see a slight reduction in fano factor in the trained group ($mean_{naïve}$= 0.53, $mean_{trained}$= 0.50), but that difference is not statistically significant (*p* = 0.514, $CI_{difference}$ (95%) = [-0.132, 0.061]).

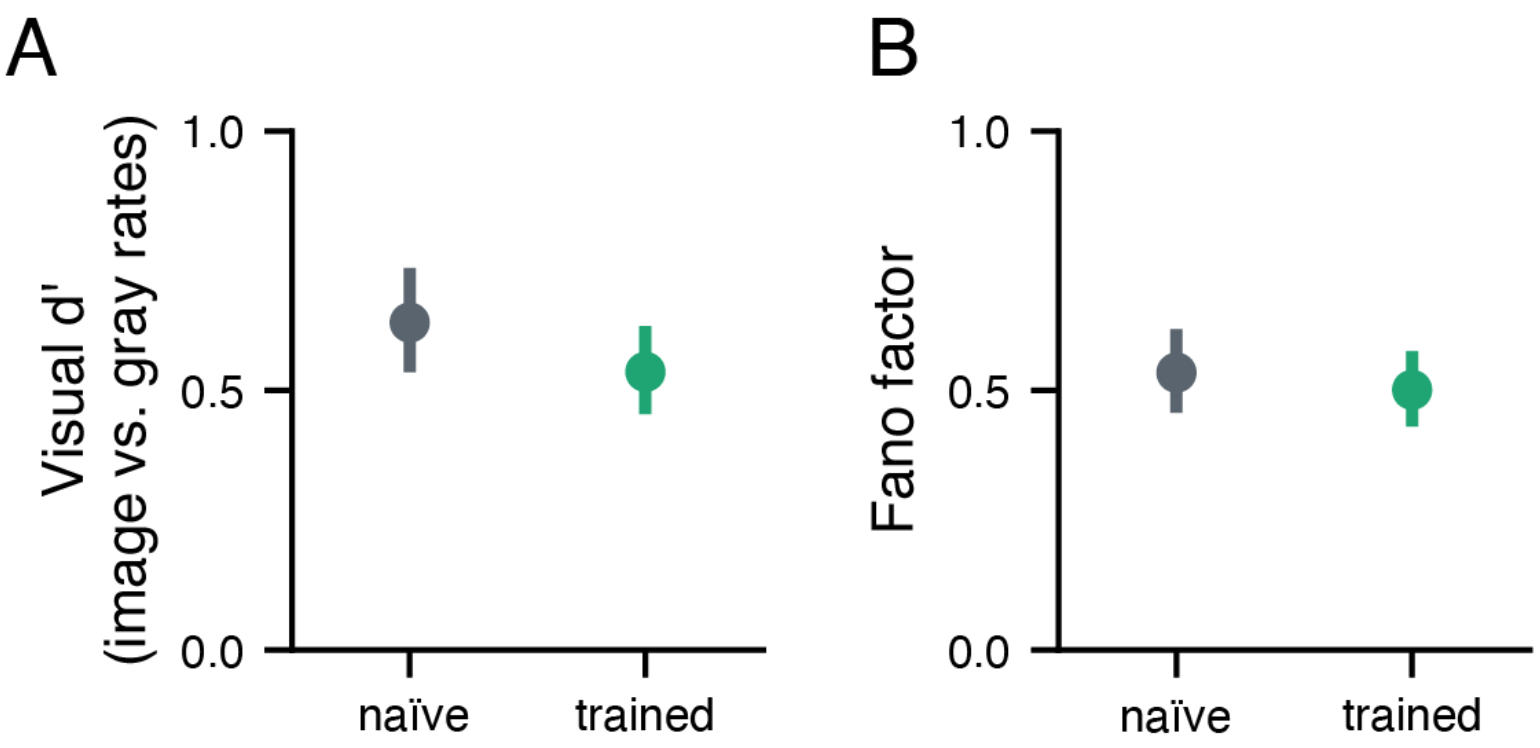

Figure R3. Naïve and task-trained recordings do not differ in their response vigor or fano factor. (A) Mean estimates of visual drive for both experimental groups, collapsed across sites. (B) Mean estimates of the Fano factor, computed as the ratio between the mean firing rate and divided by the variance across images, for both experimental groups, averaged across sites. Error bars show the 95% CI across draws.

3.6. The authors state that the neural changes were much smaller than the behavioral changes. But I could not find where the behavioral results of the naïve, untrained group are coming from. Were these the initial performances of the trained monkeys? Please clarify. This is important since, as discussed in the perceptual learning literature, learning a perceptual task can be conceived of as consisting of different (overlapping) phases, with in the first phase the animal learning the basic task (the task rules) and later a more "perceptual" phase. It is likely that IT is not involved in basic task learning but is (partially) involved in the "perceptual" phase. If stimuli are relatively easy to separate perceptually, then most of the behavioral task improvement will come from task learning, and thus a large gap between behavioral improvement and IT selectivity changes is expected. Hence, how was the behavioral improvement assessed: describe what was taken as "naïve" data (and discuss its implications) and show learning curves. Also, how much did the behavioral improvement differ between animals, and were these interindividual differences related to neural changes (although this might be hard to assess in a between-subject design and with anatomical differences in recording locations between subjects)?

We concur with the reviewer's analysis that object training consists of multiple overlapping phases: a basic task structure and a perceptual learning phase.We initially assumed the naïve monkeys were unfamiliar with both the task structure and the training objects. Yet, we fully agree that the relative importance of the training aspects on the monkey's trained task performance matters for our interpretation of our findings. We do not expect that task structure training reconfigures IT responses, but rather that the observed differences are specific to the perceptual learning part, that is, the feedback and experience collected with these specific objects. Critically, if the perceptual learning component is substantial (i.e., objects are difficult to discriminate), we would expect IT changes. Conversely, if discrimination is trivial, most learning would involve task structure, with minimal IT plasticity. Therefore, we analysed the training data of two subjects from the trained group (Fig S2, the data for Monkey B was not recorded). At the start of training, the monkeys were already fully familiar with the task structure, yet they had never seen

these specific objects before. We estimated their performance using a sliding window approach (window size: 500 trials) over the course of training (Fig S2A) to obtain subject-specific learning curves. Comparing performance between the first 500 trials and a later set of trials shows a substantial perceptual learning effect in both subjects (Fig S2B). Moreover, for these two subjects, we observe that performance gains are comparable (Fig S2B). This also suggests that we would not expect large interindividual variation in IT plasticity. Taken together, despite their task familiarity, monkeys showed performance gains that are attributable to their perceptual learning about the training objects.

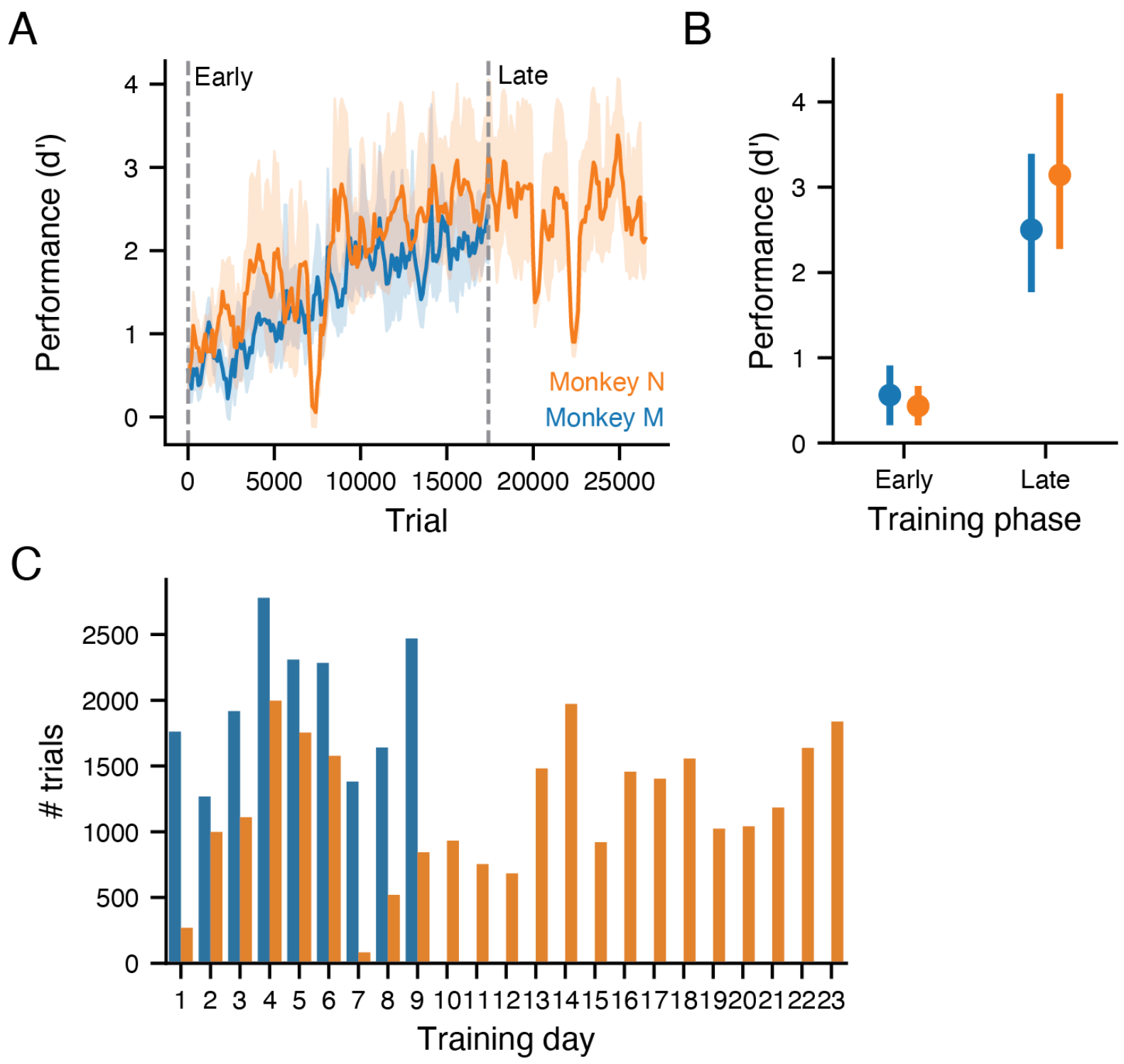

Figure S2. Object discrimination performance improves gradually during training. (A) Learning curves task-trained monkeys. Performance is estimated using a sliding window of 500 trials for each object; the x-axis indicates the starting trial of each window. Shaded areas show 95% confidence intervals of the mean bootstrapped across the eight objects. Dashed vertical lines mark the early and late training periods analyzed in panel B. (B) Comparison of early versus late training performance. Monkeys showed near-chance performance early in training but robust discrimination late in training (trial window: 17400-17900 for both subjects), demonstrating significant improvement over the training period. (C) Number of completed trials per session for each subject. Monkeys completed hundreds of trials per session from the start of training, demonstrating familiarity with the task structure despite initial unfamiliarity with the specific training objects (corresponding to low early performance in panel A).

We have now included this figure as a supplementary figure and clarified this aspect in the manuscript:

“Substantial perceptual learning was required for successful performance: Subjects, who were already familiar with the task structure, displayed low performance at the onset of the object training (d’= 0.50) and only gradually improved their

discrimination abilities over the course of the training (d' = 2.82, Fig S2), thus indicative of object-specific learning."

Results - Task training enhances task-relevant information in IT

3.7. The data on the orthogonal/identity invariant dimensions are interesting, especially their (average) consistency with the IT-like models. However, it appears that object-invariant decoding of the rotations was low (Figure S8) and lower than in their previous study (Hong et al.). Intuitively, the poor decoding of object-invariant rotation makes sense since rotating an object alters its 2D shape in the image. The latter is not the case for size and translation changes, which may make them easier to decode. Interestingly, some ANN IT-like models showed large training changes for rotation, some negative and some positive. Any explanation? Was there a correlation between these training changes for the models across rotations?

As in the earlier study by Hong et al. (2016), decoding an object's rotation proved the most challenging in our IT recordings. The decoding of rotation-based dimensions also did not significantly improve with task training (Fig S14, formerly Fig S8), and we observed, on average, that models tended not to change along rotation-based dimensions either. This held for all models, irrespective of their IT-likeness. However, individual models showed large variability in rotation-based changes. What accounts for this? This was due to how we computed the change in IT and the models:

$$\%\Delta_{\phi} = \left(\frac{\phi_{trained}}{\phi_{naïve}} - 1\right) * 100$$

Since decoding in the naïve networks tended to be close to or around zero, this led to a very small denominator. If even a small change occurred by chance in the trained networks, this then led to a large positive or negative change because it was divided by this small denominator. This division-by-small-number effect is visible in the large scaling of the y-axis for all rotation-based metrics in Fig 6D. IT data show the same pattern: large confidence intervals (grey bands in Fig 6D) for rotation metrics due to low baseline decoding. Critically, when averaging across models, rotation decoding showed no systematic change—matching the pattern observed in IT.

3.8. The higher consistency between behavior and trained IT neural data is interesting. Is it possible to examine the consistency of the naïve/trained IT data with behavioral data obtained at different points during the behavioral training? When do naïve versus trained IT-behavior consistency diverge during learning (related to point 6)?

We agree that it would be valuable to understand the time course of IT plasticity. Unfortunately, we did not collect IT recordings at intermediate timepoints during training and thus cannot track how IT-behavior consistency evolved during learning. We have included this suggestion in the discussion as a future direction.

"Answering these questions about the time course of IT plasticity and its relationship to behavior will require new experimental paradigms that can track neural changes at multiple temporal scales while maintaining measurement stability as well as computational models that can express these different hypotheses."

Discussion - Limitations and Future Directions

3.9. The authors should discuss in more detail the impact of different categorization paradigms on learning effects in the ventral visual stream and where their task is situated in this "population" of tasks. I expect that the kind of changes one will see in the ventral stream (V4 / IT) and the models depend on the nature of the categorization paradigm. For instance, in some studies, the subjects learned to discriminate small differences (e.g., orientation or contrast) between well-visible stimuli, in others to discriminate noise-masked stimuli, and in other studies to categorize multiple stimuli in the same group with small differences at the category boundary, etc. In the present study, subjects needed to categorize 10 objects rendered at different orientations, locations, and sizes, and presented upon complex scenes. This is like subordinate object classification, but with different scene backgrounds.

We agree with the reviewer that the stimuli and the specifics of the training paradigms critically determine the locus and magnitude of learning effects in the ventral visual stream and beyond. We now situate our study within this literature:

"Our finding that IT undergoes moderate functional changes raises a natural question: why did IT encoding change to the degree that we observed, and why not more or less? Prior IT training studies (for review, see Op de Beeck & Baker, 2010) span a wide range of stimulus statistics, task structures, and training demands, making it difficult to isolate which factors determine the magnitude of plasticity. For instance, stimulus complexity has ranged from simple, yet highly controlled feature configurations (De Baene et al., 2008; Vogels & Orban, 1994; Baker et al., 2002; Adab et al., 2014) to complex objects (Logothetis et al., 1995; Op de Beeck et al., 2006, 2008), morphed objects (Freedman et al., 2003, 2006; Gillebert et al., 2009; Kietzmann et al., 2016) and complex patterns (Kobatake et al., 1998), though nearly all presented stimuli on blank backgrounds at fixed positions. We here employed particularly complex stimuli — 3D objects rendered on varied scene backgrounds in random positions, rotations, and scales, adding to the diversity of the existing stimulus type considered in prior studies. While our paradigm was a standard delayed-match-to-sample object discrimination task with a two alternative forced choice between the target and distractor, presented side-by-side on the choice screen (similar to Sigala & Logothetis, 2002), many prior IT training studies adopted temporal versions of this paradigm requiring same/different judgements instead (e.g., Freedman et al., 2003; Op de Beeck et al., 2008; Vogels & Orban, 1994; Logothetis et al., 1995) or a go/no-go paradigm tied to category membership (e.g., Pearl et al., 2024). Moreover, we deviated from prior studies by relying on a training paradigm that reinforced a large number of discriminations, compared to a single binary discrimination used in most prior studies (e.g., Baker et al., 2002; Freedman et al., 2003; Pearl et al., 2024). Each of these design choices likely affects the magnitude of learning-induced changes in IT. However, the existing diversity in findings makes it challenging to isolate which factors primarily drive observed effect sizes. To address this challenge and understand what drives IT's plasticity, we turned to computational modeling."

Adopting a systems-level perspective clarifies IT's role in object learning

Which factor(s) is (are) determining the performance and learning effect in this task: the segmentation from the complex image background and/or the position-, size-, and rotation-invariant object categorization?

We now discuss this possibility more explicitly in our discussion:

“At the core of the computational problem solved by the brain and ANNs during object learning is that of invariance, the ability to identify objects over a large range of viewing conditions (DiCarlo, Zoccolan, & Rust, 2012). In our study, successful performance required invariance to an object's position, size, rotation, and, critically, its scene background. While invariance to intrinsic object properties is well-documented in the ventral stream (Brincat & Connor, 2004; Ito et al., 1995; Li et al., 2009; Rust & DiCarlo, 2010; Tovée et al., 1994; Logothetis et al., 1994; Logothetis et al., 1995; Op de Beeck & Vogels, 2000) and commonly encountered during natural viewing, our task imposed an additional demand: segmenting objects from highly variable backgrounds that, unlike natural viewing contexts (e.g., Davenport & Potter, 2004), provided no informative cues about object identity. Prior computational work suggests that ANNs implicitly learn object-scene segmentation during object recognition training, even on natural images (Seijdel et al., 2020), and that such segmentation strategies contribute to their ability to predict neural responses (Seijdel et al., 2021; Loke et al., 2024; Djambazovska et al., 2024). To what extent did this segmentation challenge contribute to the training-induced changes we observed? While our study was not designed with appropriate control conditions to directly test whether training improved general segmentation abilities, we performed a control analysis using novel objects (not part of the training set) displayed on backgrounds similar to those encountered during training. This analysis revealed no significant decoding benefit for these novel objects (Fig S8), suggesting that training did not yield general improvements in background-invariant representations. However, this does not rule out object-specific segmentation processes that may have contributed to improved discrimination of the trained objects. Future studies with explicit segmentation controls could further clarify to what extent segmentation demands shaped the distributed learning processes we have identified.”

Discussion - Adopting a systems-level perspective clarifies IT's role in object learning

Minor comments.

3.10.1. When discussing the older literature in the Introduction, Results, and Discussion, the authors should avoid lumping together different tasks or categorization paradigms, and instead clearly point out the distinctions (see main comment 9 above). In addition, they should integrate their findings and the older literature with the visual perceptual learning work in the ventral stream (including PIT studies), which is highly relevant for addressing visual learning.

We have revised our discussion of prior literature to distinguish more clearly between different task paradigms and training approaches (cf. point 3.9). We have also integrated additional work on perceptual learning in PIT:

Adab, H. Z., Popivanov, I. D., Vanduffel, W., & Vogels, R. (2014). Perceptual learning of simple stimuli modifies stimulus representations in posterior inferior

temporal cortex. Journal of Cognitive Neuroscience, 26(10), 2187–2200. https://doi.org/10.1162/jocn_a_00641

Adab, H. Z., & Vogels, R. (2016). Perturbation of posterior inferior temporal cortical activity impairs coarse orientation discrimination. Cerebral Cortex (New York, N.Y.: 1991), 26(9), 3814–3827. https://doi.org/10.1093/cercor/bhv178

If we overlooked additional relevant work, we would appreciate more specific pointers. We summarize these studies as part of the introduction on prior training studies in IT:

“Even extensive training on orientation discrimination with faint gratings, stimuli typically associated with earlier visual processing, produces behaviorally relevant changes in posterior IT (Adab et al., 2014; Adab & Vogels, 2016).”

Introduction

A related point is that it is not clear what exactly is meant by “learning new objects” (Introduction; line 5).

We also corrected the formulation in the introduction to be more specific:

“As a result, identifying new kinds of objects – even in adulthood – is pivotal for adapting swiftly to situational demands and thriving in unfamiliar environments.”

Introduction

3.10.2. Figure 2E: The authors write that they computed Euclidean distances between images, but the left figure panel shows “corr(r)”, suggesting a correlation-based distance metric. Please correct.

Thank you, we have corrected this.

3.10.3. Line 263: “Data from B and C in annotated for comparison”. Please clarify what is meant here.

Thank you, we have now added more information:

“The site counts and mean estimates from panels B and C are annotated for comparison.”

Figure 3 - Legend

3.10.4. Line 569: “This highlights the role of anatomical considerations in future studies.” It was not obvious to me what is meant here with “anatomical considerations”. Please clarify.

Thank you, we have added the following clarification:

“While decoding analyses like the ones used here are only a proof of principle that information is suitably formatted for downstream readout, it is also likely that such downstream populations do not have access to the entirety of IT responses or even the subset of populations we sampled for this study. Understanding how anatomically sparse these downstream projections are is a promising direction for improving current computational models.”

Discussion - Adopting a systems-level perspective clarifies IT's role in object learning

3.10.5. Line 586 and following: "A direction for future research is to test whether the magnitudes of neural changes induced by object vs. category training exceeds what would be expected based on the visual dissimilarities between objects." This sentence is not clear to me (object versus category training…) – please clarify.

Thank you, we have now clarified this:
"A promising direction for future research is to study the impact of categorical vs. visual similarity on the magnitude of neural changes in and beyond IT."

Discussion - Beyond IT: The Role of Downstream Networks in Object Learning

3.10.6. Line 726: typo?: canonical.

Thank you, we have corrected this.

3.10.7. Line 779: Please provide the bandpass filter settings (and filter order).

Thank you, we have added this information to our methods section:

"All neural activity was filtered using a 54-order finite-impulse-response band-pass filter (300-6000 Hz), yielding a 55-tap filter that attenuates frequencies outside the passband."

Methods - Large-scale multi-electrode recordings in the inferior temporal cortex - Electrophysiological recordings

3.10.8. Line 780: What is the difference between multi-unit activity and multi-unit spikes?

We had used it interchangeably. However, to increase clarity, we have now restricted our usage specifically to multi-unit activity.

"Most data contained multi-unit activity. Spike events were detected after data collection. In particular, a spike event (multi-unit) was detected when the voltage (falling edge) exceeded more than three standard deviations of the filtered raw voltage values.

Methods - Large-scale multi-electrode recordings in the inferior temporal cortex - Electrophysiological recordings

3.10.9. Were the data normalized per unit before computing Euclidean distances?

No, we did not apply any additional preprocessing beyond what was part of the standard preprocessing pipeline.

3.10.10. Line 854: "The inputs to the regression models were scaled based on the training data split." Scaled = normalized (z-scored?)?

Yes, exactly. We have now clarified this:

"The inputs to the regression models were z-scored based on the training data split."

Methods - Population-level object decoding

3.10.11. The manuscript states in several places that De Baene et al. did not find an effect of categorization learning in IT. This is inaccurate. That study did report a categorization learning effect in IT—albeit small—restricted to the task-relevant feature dimension when comparing pre- and post-training recordings. I recommend revising these statements accordingly.

Thank you for pointing this out. We have corrected these statements:

“Indeed, several studies have not observed either small or no changes in IT with category training (De Baene et al., 2008; Op de Beeck et al., 2008; Vogels & Orban, 1994).”

Introduction

“For example, training macaques to categorize or discriminate objects can increase single-neuron selectivity for trained object discriminations relative to non-trained ones (Baker et al., 2002; Freedman et al., 2003, 2006; Kobatake et al., 1998; Logothetis et al., 1995; Pearl et al., 2023; Sigala & Logothetis, 2002), though some studies report small or no significant changes (De Baene et al., 2008; Op de Beeck et al., 2008; Vogels & Orban, 1994).”

Introduction

“However, while some studies suggest that training-induced plasticity increases the selectivity of IT responses (Baker et al., 2002; Freedman et al., 2003; Kobatake et al., 1998; Logothetis et al., 1995; Sigala & Logothetis, 2002), others only observed minor differences in selectivity (De Baene et al., 2008).”

Result - Task training enhances task-relevant information in IT

Reviewer #4 (Remarks to the Author):

This is an interesting study that investigates how object learning in a visual task induces plasticity in the macaque inferior temporal cortex, a critical region for visual object recognition. The authors trained six monkeys on a delayed-match-to-sample task involving eight object categories, with images carefully designed to systematically test object recognition in IT cortex. Neural activity was then recorded using chronically implanted multielectrode arrays across multiple IT subregions in both trained and naïve animals. The results showed that training enhanced object selectivity in the IT cortex. The authors presented this effect using multiple complementary approaches, indicating improvements both at the single-site level and at the population level, including representational geometry and linear decodability. The authors then employed hierarchical neural network models to simulate IT plasticity. Unfortunately, despite the strengths of the study, the manuscript still requires substantial revisions to address key issues.

We thank the reviewer for their thoughtful and constructive feedback. We have addressed each of their concerns and believe the manuscript is substantially strengthened as a result. Below, we detail our responses:

Major concerns:

4.1. The manuscript provide no information about the training phase, such as the total number of sessions, number of trials per session, the overall duration of training. These details are critical, especially in a study focused on plasticity, since the magnitude and nature of neural changes might closely tied to training intensity and time course. Moreover, it would be important to report the behavioral learning curves, error rates, and performance progression across training sessions. These information must be provided for each animal separately.

We have now provided additional information about the training phase for two of our trained monkeys in the table below and in Fig S2. The training of monkey B was not recorded.

**Subject**	**Monkey N**	**Monkey M**
**# sessions**	23	9
**# trials (total)**	27034	17808
**# Trials per session (mean [min, max])**	1175.39 [82, 1997]	1978.67 [1268, 2779]
**Training duration (days)**	44	12

We have also added this information to the methods section:

“At the start of the training, subjects were already familiar with performing a match-to-sample task, yet unfamiliar with these specific training objects (cf. Fig S2C). Subjects gradually improved their performance over the course of the training (Fig S2A), and they transitioned to the testing phase once their training performance saturated (for details on this criterion, see Rajalingham et al., 2015). Monkey N reached criterion after 23 sessions (44 days, 27,034 trials); Monkey M after 9 sessions (12 days, 17,808 trials). Training data for monkey B was not recorded.”

Methods - Monkey training and behavioral tasks - Object training and active binary object discrimination task

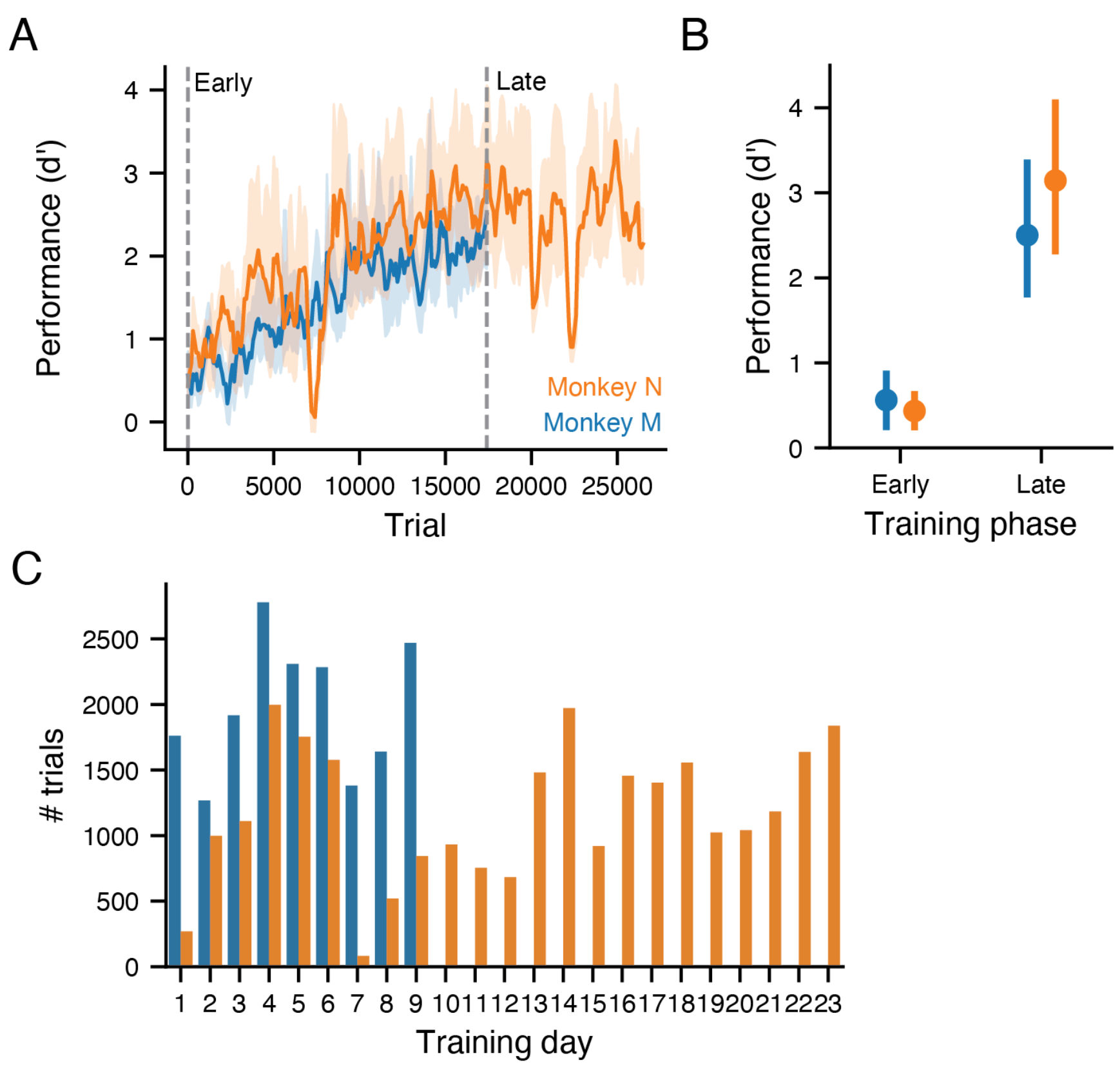

Figure S2. Object discrimination performance improves gradually during training. (A) Learning curves task-trained monkeys. Performance is estimated using a sliding window of 500 trials for each object; the x-axis indicates the starting trial of each window. Shaded areas show 95% confidence intervals of the mean bootstrapped across the eight objects. Dashed vertical lines mark the early and late training periods analyzed in panel B. (B) Comparison of early versus late training performance. Monkeys showed near-chance performance early in training but robust discrimination late in training (trial window: 17400-17900 for both subjects), demonstrating significant improvement over the training period. (C) Number of completed trials per session for each subject. Monkeys completed hundreds of trials per session from the start of training, demonstrating familiarity with the task

structure despite initial unfamiliarity with the specific training objects (corresponding to low early performance in panel A).

4.2 Similarly, the description of neural recordings lacks important information. The manuscript should provide details on the number of recording sessions, the number of images presented per session, and quality metrics such as signal-to-noise ratio for each electrode and the yield of each array. Information on the timing of recordings relative to the training phase (e.g., early vs. late training) should also be reported. As with the behavioral data, these details should be provided for each animal and each array separately, since variability across subjects and recording sites may strongly influence the interpretation of the results.

We have now added the following additional details in the Methods section under the subsection Recording Timelines:

“In the trained group, neural data were collected across 41, 57, and 10 recording sessions for Monkeys N, M, and B, respectively. In each session, monkeys viewed a set of 1,320 unique images, with images repeated multiple times, resulting in approximately 2,000–8,000 total image presentations per recording day. Recordings in trained animals began after completion of object training, with neural data collection starting approximately 8 months (Monkey N) and 6 months (Monkeys M and B) after training.

In the task-naïve group, recordings were collected across multiple days. Monkey S contributed data from 3 recording days with 640 unique images per session, while Monkeys C and T contributed data from 65 and 68 recording days, respectively, with 2,560 unique images per session. As in the trained group, images were repeated within sessions, yielding approximately 2,000–8,000 image presentations per day.”

Methods - Large-scale multi-electrode recordings in the inferior temporal cortex - Recording Timelines

Additionally, we now provide detailed quality metrics for each animal and hemisphere in Fig S3B:

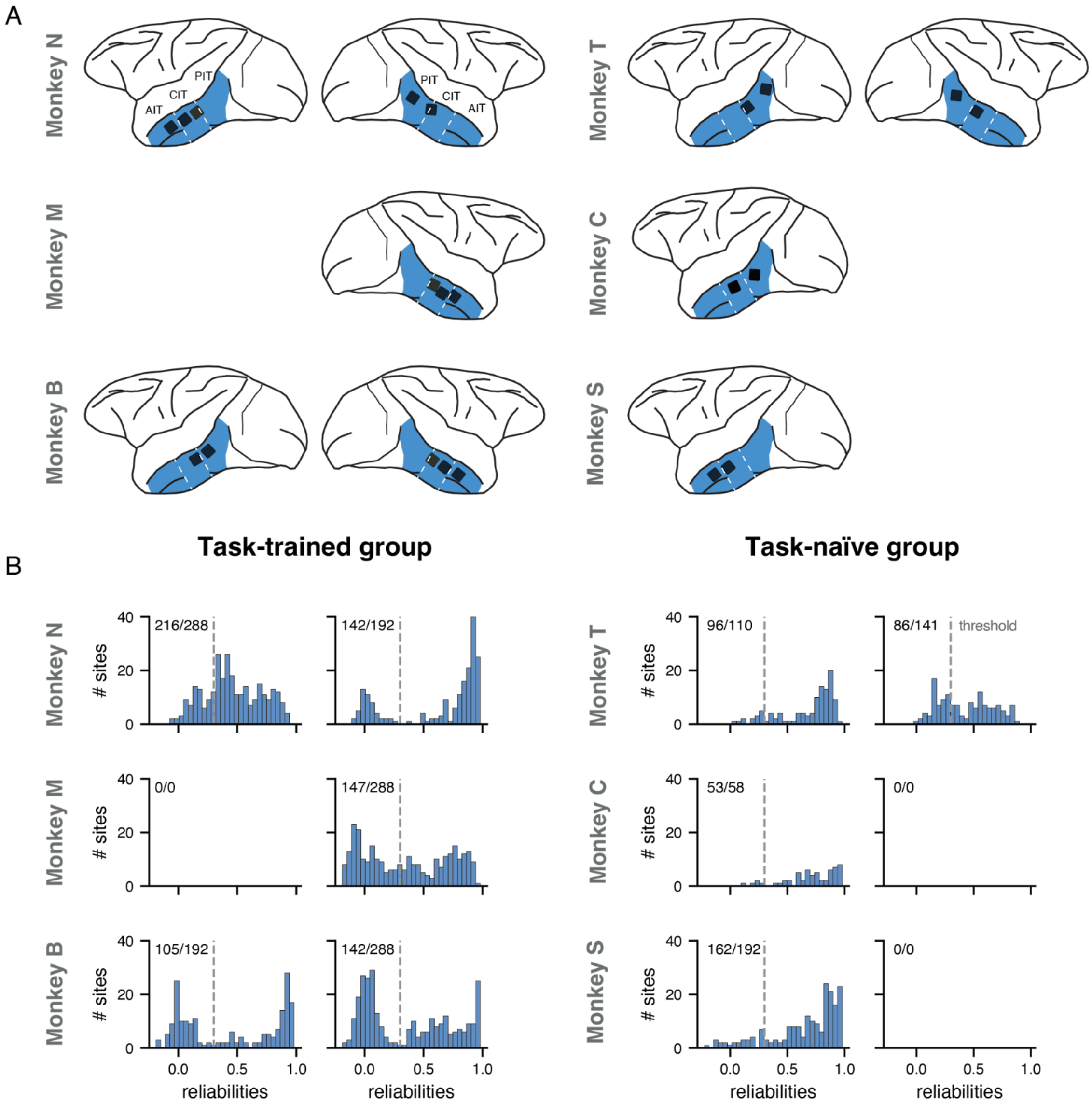

Figure S3. Micro-electrode array placements & electrode signal-to-noise ratio. (A) All subjects had chronically implanted Utah arrays in various locations along the posterior-to-anterior axis in the inferior temporal cortex. Note for subjects with bilateral arrays shown, recordings were performed consecutively in both hemispheres (see Methods for further details). Arrays were classified as AIT, CIT, or PIT according to their position relative to the anterior and posterior middle temporal sulci (annotated by the grey dashed lines). For our main analysis, recording sites were pooled across IT subregions, treating all recording sites as random samples from an average IT population. (B) Signal-to-noise ratio of all recording sites per hemisphere and subject was computed as the split-half reliability across repetitions (see Methods for further details). The dashed line indicates the reliability criterion of 0.3 for site inclusion adopted in the main analysis. Each panel is annotated by the effective yield (i.e., sites that passed the reliability criterion) and the total number of available recording sites. The panel layout follows that of (A).

Moreover, there is no analysis reported within each animal. All of the main analyses should also be presented at the single-animal level. I understand that such analyses might not reach statistical significance due to limited power, but they are essential for evaluating replicability and robustness of the reported effects.

We fully agree that demonstrating robustness at the individual animal level is essential. To this end, we repeated all of our main analyses for every subject separately. We show the results in the figure below. While this is not the appropriate level to draw statistical conclusions, as pointed out by the reviewer, we do see qualitative differences for all examined metrics: Recordings from task-trained subjects tend to score higher on all examined metrics than recordings from task-naïve subjects.

We now mention this analysis in our Results section:

“Do these training-induced differences exceed what would be expected from interindividual variation alone? That is, could the observed effects be driven by differences between a few subjects, irrespective of their training history? To test whether our observed training differences are robust to interindividual variation, we implemented two additional analyses. First, we qualitatively replicated each of our training effects for each animal. Fig S6 demonstrates that even with limited data and varying numbers of recording sites, we observed the same qualitative differences between task-trained and naïve subjects as shown in Fig 2.”

Results - Task training enhances task-relevant information in IT

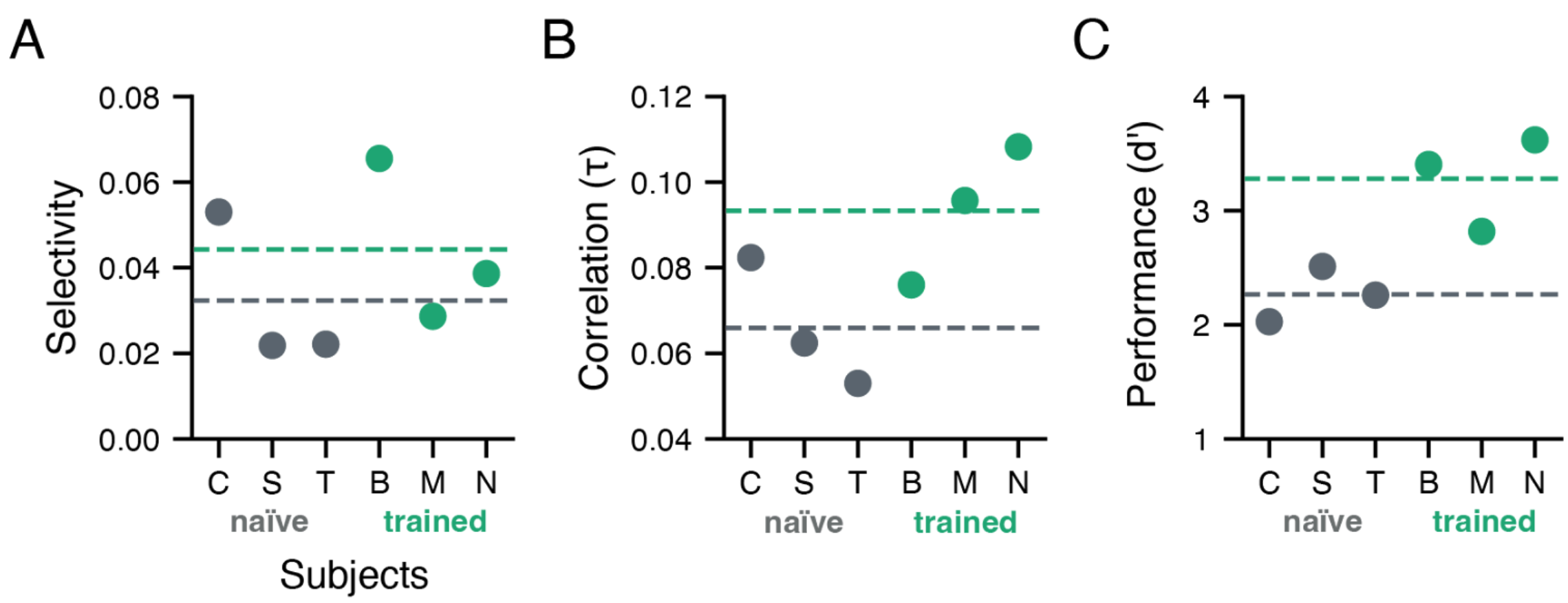

Figure S6. Single-subject level analyses. To determine whether training effects are observable at the individual subject level, we computed our key metrics (as in Fig 2) using the full dataset available for each subject. We included only sites with split-half reliability higher than 0.3, but did not subsample or match for reliability differences between subjects. Grey and green dots show individual subject estimates for task-naïve and task-trained animals, respectively. Dashed lines show the mean across the three subjects in each condition. Task-trained subjects tend to show higher values than naïve subjects across all metrics, indicating robust training effects at the individual level.

4.3 The central conclusion from the neural data relies heavily on Figure 2L-M, which compares delta-d' between IT decoding and behavior. However, the approach used to derive these results raises several concerns. First, the delta-d' in IT is obtained by fitting a log function to the decoder performance as a function of the number of IT sites, but it's not clear how exactly delta-d' is calculated.

We have now provided further clarification to address the reviewer's comments. Our central conclusion is based on Figure 2K, containing our full dataset (N=159 sites per draw and group) without any extrapolation or assumption about saturation. Figure 2L shows the data from Figure 2K as the difference between trained and naive decodes, where we subtract the naïve pool decoding estimates from the trained pool metrics for each of the 1,000 draws. This step involved no fitting and is merely a different way to plot these results. All of our subsequent modeling work is based on an equivalence to these 159 recording sites in a task-naïve subject (cf. Methods - Aligning base models to task-naïve IT responses, Fig S10). We now clarify this in the legend of Figure 2:

"(L) The sampled IT population sizes (i.e., 159 sites) are sufficient to approximate the behavioral performance level of the trained subjects. The horizontal shaded area shows the 95% CI of the trained monkeys' mean accuracy (pooled, across 3 subjects) for the same images. We fit a log function to the IT decodes (dotted lines) to identify the crossing point of the IT decodes with the average trained monkeys' behavioral accuracy (gray horizontal dashed line). (M) Comparison between differences in discrimination performance at the level of IT and behavior. IT differences show the same data as in K and are based on draws of 159 sites. All error bars and shaded areas depict the 95% confidence interval (CI) of the central tendency metric across draws. "

Moreover, this fitting procedure introduces strong assumptions, including that the readout of IT activity in the brain is linear and that decoder performance continues to increase logarithmically with larger sample sizes. However, the neural decoding performance may reach a ceiling, and alternative fits (e.g., saturating or sigmoidal functions) may be more biologically plausible. The authors should justify their choice of function and demonstrate that their conclusions are robust across different fitting approaches.

The purpose of the fitted function in Fig 2L is to illustrate that larger differences in decoding could be observed if we had an even larger dataset. We appreciate the reviewer's critique and have implemented a version of this function that plateaus at the upper bound of the observed behavioral data (see figure below). Here, we enforce that the fitted function cannot exceed the 95%CI of the behavioral performance. This approach yields very similar results to those we show in Fig 2L.

However, we emphasize that our central conclusions are based on the empirical data at 159 sites (Fig 2K), not on these extrapolations. Since this function has mostly illustrative value and to stay consistent with other analyses, we retain the log function in Fig 2L.

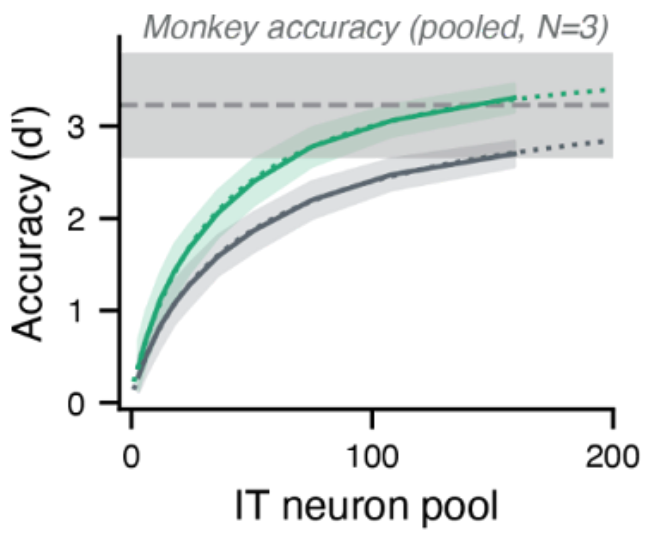

Figure R4. Alternative function fit for Fig 2L. We replaced the initial unbounded log-function with one constrained to saturate at the observed behavioral performance (97.5 percentile of the pooled monkey performance estimates).

Second, even accepting the log fit, Figure 2L implies that the performance of an IT-based linear decoder would reach the monkeys' behavioral range with approximately 200 neurons and would exceed behavioral performance with a greater number of neurons even in naïve animals. This finding is difficult to reconcile with the claim that behavior can be directly compared to a linear readout of IT activity in order to reject H1 or H2.

We thank the reviewer for raising this concern and allowing us to further clarify this point in our Discussion. We use decoding from neural recording sites as a way to estimate linearly separable information in IT. In this study, we do not assume the decoding results to be interpreted as hypotheses for how behavior might be read out of IT (e.g., from IT to motor circuits). Rather, we evaluate whether information in IT is suitably formatted for downstream readout, making it capable in principle of supporting behavioral performance. We did not intend to imply that it is, therefore, evidence for how such information is used by downstream areas. While we do know from stimulation and inactivation studies that IT is causally involved in object recognition behavior, we do not know how downstream areas specifically access this information to inform perception. Additionally, each recording site likely samples from multiple neurons, meaning the number of recording sites does not directly correspond to the number of biological neurons contributing to behavior.

We now clarify this:

"While decoding analyses like the ones used here are only a proof of principle that information is suitably formatted for downstream readout, it is also likely that such downstream populations do not have access to the entirety of IT responses or even the subset of populations we sampled for this study. "

Discussion - Adopting a systems-level perspective clarifies IT's role in object learning

4.4 Most of the p-values and confidence intervals are presented without corresponding effect sizes or sufficient context for the underlying statistical analyses.

We appreciate the reviewer's attention to our statistical reporting, and we have taken this opportunity to revise and verify all reported statistics. In general, we perform two-sample bootstrap tests as we describe in our methods section.

All our IT-metrics are standardized measures (i.e., they have interpretable and bounded value ranges between -1 or 0 and 1 for selectivity and Kendall's Tau and 0 - 5 for d'). As such, their relative and absolute difference is an effect size. We now clarify these aspects in our methods section:

"[...] to test for differences between groups (1000 draws sampled pools with replacement), we performed a two-sample bootstrap test by computing the differences in the bootstrapped distributions and derive a p-value from the probability of zero under that difference distribution (i.e., we construct the confidence interval of the difference distribution and check whether it contains zero). We report the absolute and relative difference of our standardized metrics (e.g., selectivity or d') as a measure of effect size."

Methods - Statistical analyses

Moreover, we now report our main results (incl. effect sizes) in the following formats throughout the manuscript:

"We observed that object predictions from the trained IT populations showed a significantly higher accuracy compared to naïve IT populations (Fig 2K, Trained: 3.31 ± 0.08 (mean ± SD), Naïve: 2.70 ± 0.08, Difference: 0.612 ± 0.108, CIDifference (95%) = [0.398, 0.811], $p < 0.001$)."

Results - Task training enhances task-relevant information in IT

"For the properties that significantly differed between task-naïve vs. -trained IT recordings (i.e., object location, size, and eccentricity), we observed that IT-likeness introduced significant differences between model groups (upper panel, Fig 6D, Vertical position: $H(3) = 38.34$, $p < 0.001$, $\varepsilon^2 = 0.173$; Horizontal position: $H(3) = 51.03$, $p < 0.001$, $\varepsilon^2 = 0.235$; Object size: $H(3) = 45.03$, $p < 0.001$, $\varepsilon^2 = 0.206$; Eccentricity: $H(3) = 50.83$, $p < 0.001$, $\varepsilon^2 = 0.234$) with IT-like models always producing a larger increase in accuracy compared to IT-unlike models."

Results - IT-like models best predict differences along untrained dimensions in IT

Thanks to the reviewer's comment, we noticed that we had not included confidence intervals and effect sizes for some of our control analyses. We have now corrected this:

"Still, the quantitative differences were only statistically different in central IT where many recording sites were available (AIT: Difference = 0.234 ± 0.159, $CI_{Difference}$ (95%) = [-0.056, 0.534], $p = 0.16$, 24.2% increase CIT: Difference = 0.770 ± 0.166, $CI_{Difference}$ (95%) = [0.446, 1.070], $p < 0.001$, 42.2% increase, PIT: Difference = 0.254 ± 0.149, $CI_{Difference}$ (95%) = [-0.050, 0.537], $p = 0.112$, 20.2% increase)."

Results - Task training enhances task-relevant information in IT

"Repeating our analysis for the left hemisphere only, we again observed a pronounced increase in task-related information in the task-trained IT responses (Fig 3C, Difference = 0.870 ± 0.103, $CI_{Difference}$ (95%) = [0.676, 1.070], $p < 0.001$, 36.6% increase)."

Results - Task training enhances task-relevant information in IT

Minor concerns:

4.5.1. All reported p-values should include a leading zero before the decimal point

Thank you, we have corrected this.

4.5.2. Figure 1C and 1M: on the y-axis, d' is labeled as "Accuracy" which is an unusual description for d'. "Performance" would be a better choice.

We have altered all "Accuracy" labels to "Performance" in all figures.

4.5.3. Moreover, the plot currently presents only normalized d' (delta-d'). Please add a bar plot showing performance before and after training for each animal separately, as presenting the absolute values in the first plot would be more informative.

Since this study was not conducted in a longitudinal design, this is unfortunately not possible. We do, however, now provide metric estimates for each subject (point 4.2, Fig S6).

4.5.4. Line 58: the term "perception-action" has not been used elsewhere in the manuscript and requires clarification.

Thank you for noticing this. We have changed this term to sensory-motor circuits, referring to neural circuits that process sensory information and translate this information into appropriate motor commands.

4.5.5. Line 151: subfigure (M) is presented as the right panel in subfigure (L).

Thank you, we have corrected this.

4.5.6. Figure 2J: numbers on the y-axis should include a leading zero before the decimal point.

Thank you, we have corrected this.

4.5.7. Figure 2M: this is one of the main plots of the paper summarizing results in the hypothesis space (H0, H1, H2). It would be more informative if plotted with greater detail (e.g., presenting data points for each animal separately, rather than only pooled results).

This is not possible since we are analysing data between groups of individuals. We do, however, now provide all IT metrics shown separately for each subject (point 4.2, Fig S6).

4.5.8. Lines 223-232: the paragraph beginning with "How do these observed training-induced differences compare to differences in interindividual variation?" seems intended to justify the use of pooled data across subjects. If so, the term "between-group" at the end should be revised. In any case, the paragraph is vague and should be clarified.

Thank you, we have now clarified this paragraph as follows:
"Do these observed training-induced differences exceed what would be expected from interindividual variation alone? That is, could the observed effects be driven by differences between a few subjects, irrespective of their training history? To test

whether our observed training differences are robust to interindividual variation, we implemented two additional analyses. First, we qualitatively replicated each of our training effects for each animal. Fig S6 demonstrates that even with limited data and varying numbers of recording sites, we observed the same qualitative differences between task-trained and naïve subjects as shown in Fig 2. Second, we assessed these qualitative observations statistically using a hierarchical permutation bootstrapping test. This test not only captures the variability between groups but is also sensitive to variability within both groups (see Methods - Statistical Analysis). We observed that the effects linked to task training were significantly greater than what would be expected from interindividual variation for selectivity (p = 0.003, Fig S7A), representational strength (p = 0.041, Fig S7B), and object decoding (p = 0.002, Fig S7C). These results support that the observed differences are attributable to task training rather than pre-existing individual variation, validating our approach of pooling data across subjects within each condition."

Results - Task training enhances task-relevant information in IT

4.5.9. Line 243: "Addtionally" should be "Additionally"

Thank you, we have corrected this.

4.5.10. Line 243: differences between areas may be due to variation in recording quality and signal-to-noise ratio. More controlled measures are needed.

As per your suggestion in R4.2, we now also show the reliability of the recording sites per hemisphere and animal. Additionally, we have replicated our key result while applying different reliability criteria, observing a robust statistical difference, even when applying a more stringent SNR criterion. We now mention this control analysis in the methods section and have included this figure in the supplement:

"Additional control analyses demonstrate that the choice of reliability criterion did not qualitatively affect our main results (Fig S5)."

Methods - Neural and behavioral data analyses - Neural recording quality metrics and pooling

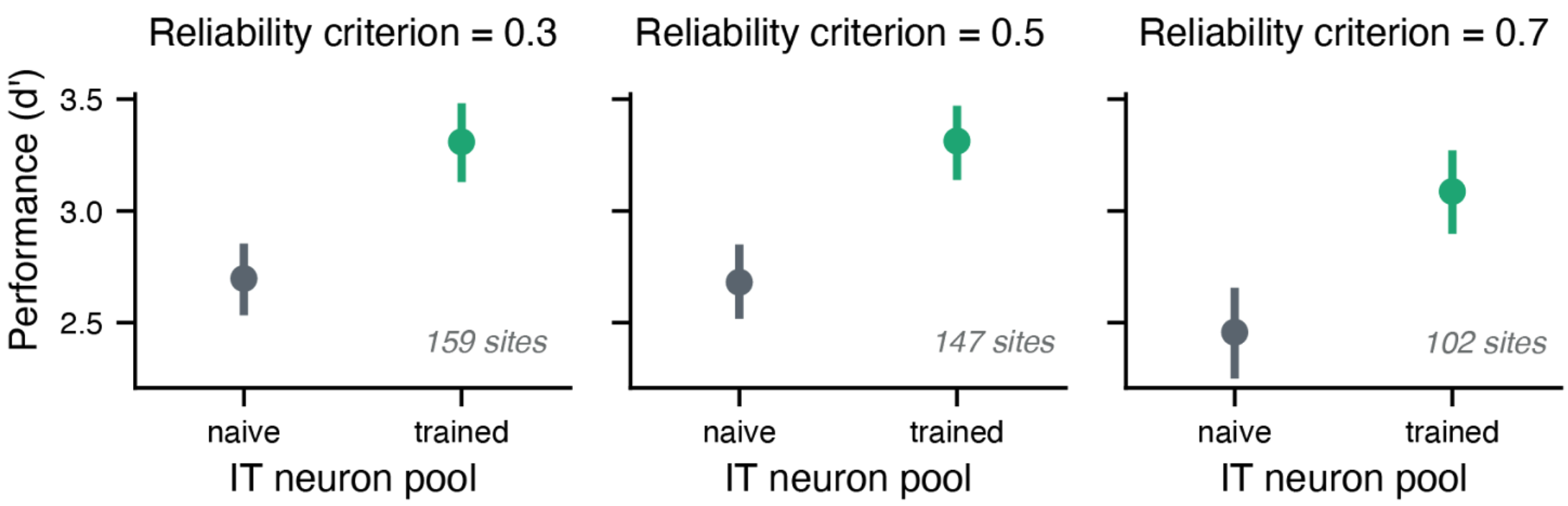

Figure S5. Training-induced decoding differences are not dependent on the choice of the reliability criterion. Neuron pools were sampled from recording sites passing a given reliability criterion. To test whether our results depend on this criterion choice, we repeated the analysis using different reliability thresholds. Although increasingly stringent reliability criteria (left to right) progressively reduced

the number of available sites (annotated in grey), training-induced differences in decoding accuracy remained significant across all thresholds (all $p < 0.001$).

4.5.11. Line 269: wording should be corrected to "we would expect."

Thank you, we have corrected this.

4.5.12. Figure 6D: it is unclear what the gray band (95% CI of neural data) represents. A 'good model' should be closer to the actual performance (likely the median of the CI). If the intent is to show similarity, a scatter plot illustrating correlations might be more effective.

We thank the reviewer for this feedback. The gray band represents the 95% confidence interval around the observed neural changes, reflecting our measurement uncertainty. If a model's change falls within this band, we consider it consistent with the neural data. While the median represents our best estimate, any value within the full CI is considered compatible with observations given measurement uncertainty.

Regarding the visualization choice: while a scatter plot could show correlations for individual models, our goal was to demonstrate how IT-likeness (the number of IT metrics matched) systematically affects model predictions across all behaviorally-matched trained models. The grouped distribution visualization more effectively shows this systematic relationship.

4.5.13. Figure S5 legend: "sqaures regression" should be "squares regression"

Thank you, we have corrected this.

Reviewer #5 (Remarks to the Author):

I co-reviewed this manuscript with one of the reviewers who provided the listed reports. This is part of the Nature Communications initiative to facilitate training in peer review and to provide appropriate recognition for Early Career Researchers who co-review manuscripts.

Reviewer #5 (Remarks on code availability):

i cannot find the codes

We had already provided our code with the initial submission, and it is also accessible in the code availability section of the portal as:

https://osf.io/dcgze/?view_only=6dfe548c7ba24238932d247e65523053 .

Reviewer #1 (Remarks to the Author):

The revision is very responsive and clarifies a number of issues. It's now much clearer at the outset that the results fall into the "some plasticity but not fully able to explain behavior" camp. The discussion as well is significantly improved with much clearer statements about implications and potential future directions. My remaining concerns are certainly not disqualifying and mainly affect some new results that appear the responses and supplementary figures that deserve better discussion and integration into the main body.

1. There are 3 points that I believe are rather central and should not be sequestered to supplementary material. The first is learning dynamics and its gradual and irregular natures, consistent with gradual changes in representation (Fig S2). The second is Fig S8 showing specificity of learning with regard to trained objects. The third is S13 which I find a very nice approach for address the observed changes in a high dimensional space. With regard to the figure however, I fell like the discussion which focuses solely on manifold size is a little too brief. Rather I think it's the combination on increases in inter-cluster distances without changing cluster sizes that should be emphasized with the 3 metric matches: intuitively (unless I'm misinterpreting) that would imply better potential decoding, whereas increases in all distances (both within and between clusters) would not.

We thank the reviewer for the positive assessment of our revisions and for their acknowledgement of the clarity in the inferences that the newly added analyses have offered. We have now further implemented their new recommendation as follows:

1. We now show the gradual increases in task accuracy over the course of training as additional panel C in Figure 1 (added below), summarizing the information in Fig S2.

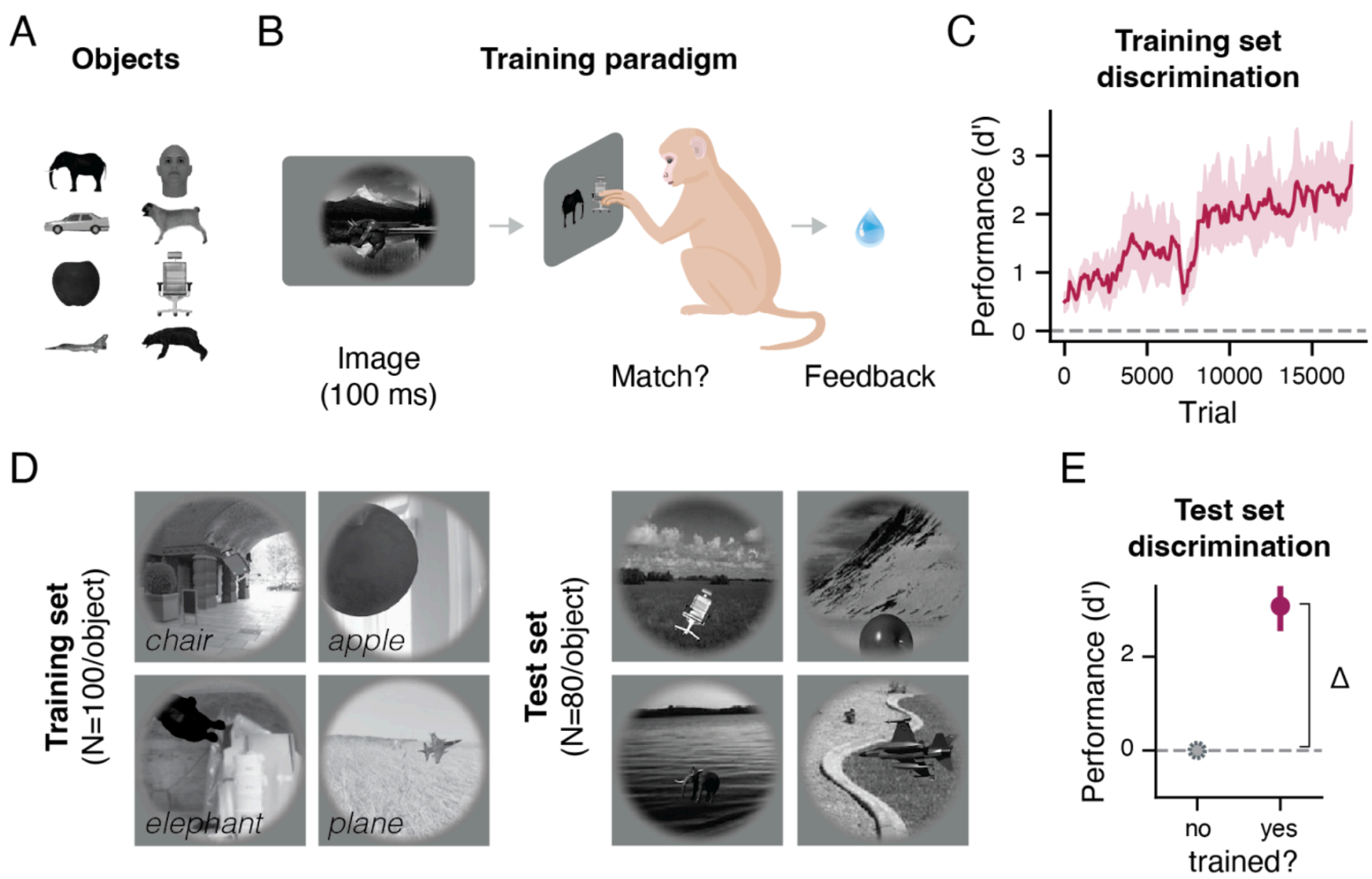

Figure R1. Excerpt from Figure 1.

2. We have now moved Fig S8 and Fig S13 into the main body of the manuscript; they are now Fig 4 and 7, respectively.

We also added a more extensive discussion of the results presented in Fig 7 (previously S13). We now clarify that all evaluated geometric changes can, in principle and independently of one another, increase linear separability. Indeed, the magnitude of inter-cluster distances (i.e., signal enhancement) sets apart various IT-like models from those that only match behavior. Similar patterns were observed for signal rotation and manifold warping. Yet, only cluster size (i.e., manifold shrinkage) appears to further separate between different levels of IT-like models. We have clarified these aspects in the Results as follows:

"To further probe what distinguishes IT-like models from the alternatives, we conducted geometric analyses of the representational manifolds before and after task training (Fig 7). Following metrics proposed by Cheng et al. (2025), we quantified changes in four distinct geometric properties that can each independently contribute to increased task-relevant information: (1) signal enhancement (increasing distance between target and non-target manifold centroids); (2) manifold shrinkage (tightening the clustering of images within each object category); (3) signal rotation (repositioning the manifold centroids relative to each other); and (4) manifold warping (altering the covariance structure across dimensions, Fig 7A). We compared these metrics across models grouped by their degree of IT-likeness (i.e., the number of IT metrics matched). While all four metrics qualitatively distinguished IT-unlike models (matching 0 metrics) from IT-like models (matching all 3 metrics). That is, signal enhancement, signal rotation, and manifold warping appeared to be associated with meeting at least one IT metric. Manifold shrinkage showed the most systematic relationship with IT-likeness, revealing graded differences across intermediate levels (Fig 7B). Specifically, the most IT-like models maintained relatively stable manifold variance between their naïve and trained states, whereas models matching fewer IT metrics exhibited increased variance after training, indicating that images formed larger, more dispersed clusters following task training. This finding provides a complementary perspective on our earlier results: increases in neural selectivity alone, achieved potentially by signal enhancement or rotation, are insufficient to reproduce all IT changes; rather, training must enhance discriminability via subtle and distributed changes across the population (e.g., via manifold shrinkage) to parallel the differences observed in IT."

Results - Task-optimized ANNs reproduce IT's task-training-induced differences

2. Another important point is the specificity of changes to IT-like layers in the models. While this is anecdotally addressed in S11, I think it deserves a more thorough treatment (and accordingly potential inclusion into the main body). First, rather than taking 3 individual layers and looking at IT specificity, represntation strength, and decoding distributions, I would instead for each of these report the percentage of models that fall within the realistic range of those parameters for all the layers. Just showing a relative simple one, like AlexNet would be fine here. But also of critical interest are the generalizations to untrained parameters shown in Figure 6. If I take the 3/3 models I would expect very poor generalization for early layers (and maybe too much for high layers?).

As suggested by the reviewer, we have now performed a more thorough analysis of the specificity of IT-like layer changes across the network hierarchy. In total, we examined 14 model layers of ResNet-152 (Fig S11A). We chose this architecture rather than AlexNet because it produced the most IT-like models in our screening procedure (cf. S12B) and would thus be the most representative of our overall results.

We observe that the layer with the highest IT encoding score (i.e., the IT-mapped layer) best captures training-induced IT differences for the selectivity and linear decodability metric (Fig S11C) across all examined layers. For representational strength, the IT-mapped layer is among the best-fitting layers, but many alternative layers fit the neural differences comparably well (Fig S11C). This fully replicates our earlier results (Fig S11B).

Next, we performed the proposed generalization test. Focusing only on models that match IT for the three IT metrics (12 3/3 models), we examined three representative layers (early control, IT-mapped, and later control, Fig S11A). Given that the object-related IT differences are best captured by the IT-mapped layer, one would expect that this layer should also best generalize to predicting object-invariant differences. In turn, the early and late control layers are expected to generalize less well to predicting these other dimensions.

Fig S11D (attached below) shows that this pattern held for most object-invariant properties. In particular, for properties that differ significantly in IT (vertical & horizontal positions, object size, and eccentricity), the differences predicted by the IT-mapped layer better align with the observed IT differences than those of the control layers. IT-mapped layers performed on par or worse for properties without significant differences (i.e., rotational properties).

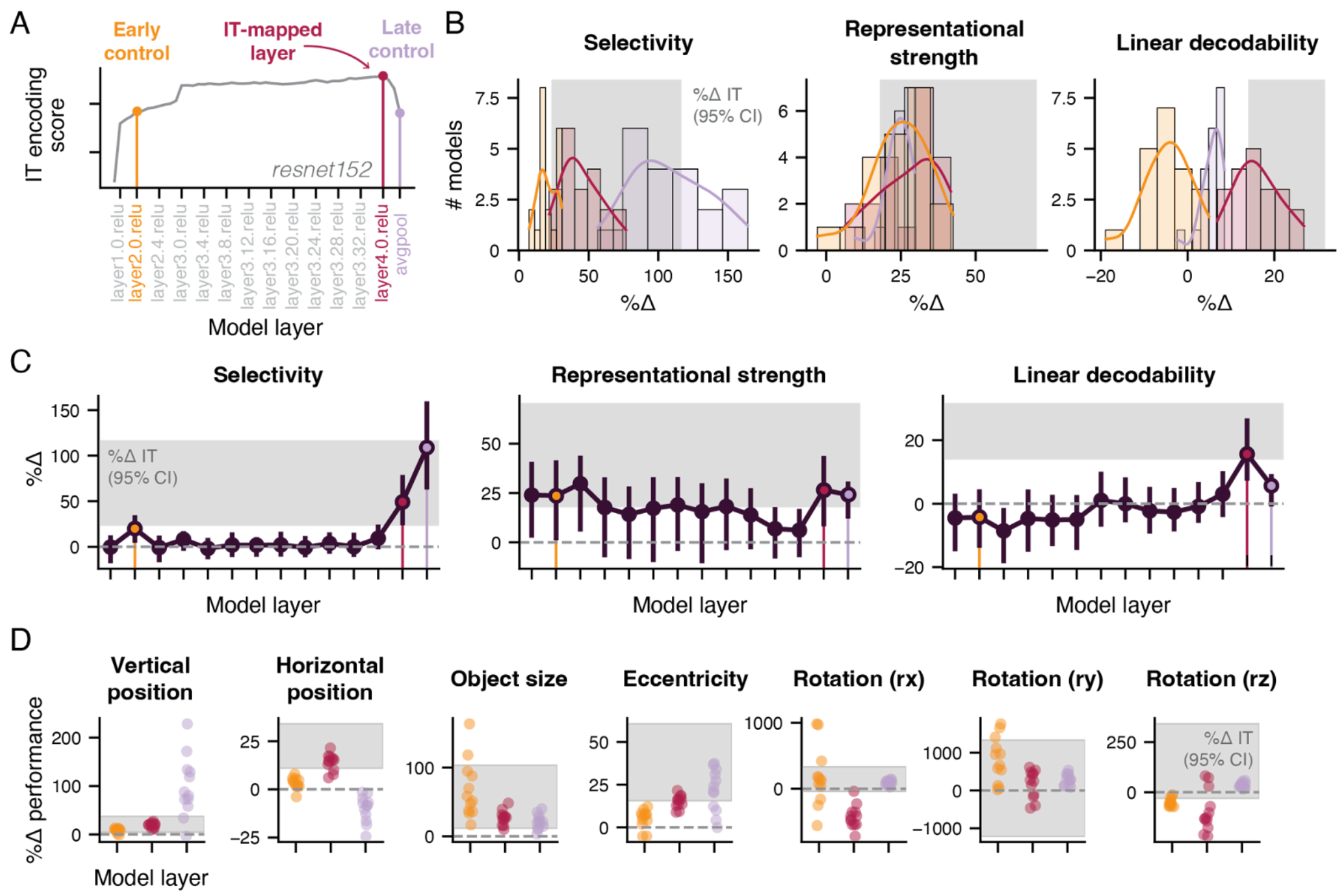

Figure S11. IT-like layers best capture neural IT differences. (A) For our main analysis, we selected the model layer showing the best encoding of naïve IT responses (i.e., IT-mapped layer, red line). To validate this approach, we compared this IT-mapped layer to two control layers for one base model (resnet152). All evaluated layers are annotated on the x-axis.: For two control layers, we performed more extensive analyses: (1) a late control layer, the next deeper layer after the IT-mapped layer, which showed the worst IT encoding score (lilac line), and (2) an early control layer, an earlier layer matched to the late control in IT encoding score (orange line). All comparisons used the same 20 trained model instances of resnet152. (B) Whereas model changes from the IT-mapped layer (red histograms) fell mostly within the observed IT changes, model

changes from the more extensively evaluated control layers (orange and lilac histograms) tended to fall outside of the empirically observed differences. (C) Examining layers across the entire model reveals that the IT-mapped layer best matches the neural data for the selectivity and decoding metric. For representational strength, earlier and later layers fit the neural data on par with the IT-mapped layer. The x-axis shows the annotated layers in (A). We plot the mean across 20 models and show the 95% confidence interval. (D) Is the ability to generalize to untrained dimensions specific to the IT-mapped layer? To assess this, we focused on models that fit all three IT metrics (N=12) and adopted the early and late control layers for predicting object-invariant training differences (cf. Fig 8). We find that for most properties, the IT-mapped layer tends to better predict the observed IT differences compared to the control layers.

We have summarized these findings in the updated supplementary figure S11 and refer to these results throughout the main body of the text:

"To test whether the observed changes are specific to the features of IT-mapped layers, we performed a control analysis where we replaced the IT-like ANN layer with other earlier and later control layers (with less IT-like encoding, sampled throughout the network for a single architecture). For selectivity and decodability, and less so for representational strength, we observed that IT-like layers most aptly captured the neural differences (Fig S11A-C)."

Results - Task-optimized ANNs reproduce IT's task-training-induced differences

"Within the IT-like models, how specific is the ability to generalize to the IT-mapped layer? To test this, we repeated our analysis comparing the IT-mapped layer to an earlier and a later control layer within one base model group (Fig S11). For the majority of object-invariant properties, we indeed find that IT-mapped layers tend to better predict IT's differences along untrained dimensions than the control layers (Fig S11D)."

Results - IT-like models best predict differences along untrained dimensions in IT

3. In the original submission I found the mismatch of the binary choice training to behavior/physiology puzzling, and now with more discussion and results, it seems even more puzzling. If I understand correctly, the extreme changes in behavioral consistency (Fig 6F), the ones far greater than would be expect from the IT data), are selectively happening for binary choice training? (If this is true, it might help to color the points in this presentation according the updating algorithm). Figure S15 shows that binary choice is distinctive for the other algorithms AND inconsistent with behavior. Finally, Figure S12 shows that decoding often completely fails (d'=0) with binary choice. Taken altogether, it seems like the most biological plausible updating is a complete disaster. Why? Does the sparseness and locality of reweighting mean changes get "trapped" in local minima? If i did a "slower" probabilistic reweigtting (or one with errors in assignment) would that help?

We thank the reviewer for revisiting this important point. We acknowledge that the results from binary-choice training can be puzzling, and we appreciate the opportunity to clarify the interpretation of the relevant figures. Based on the reviewer's description, we suspect that some confusion may arise from how several of the plots were interpreted. Below, we revisit the figures mentioned by the reviewer and clarify the conclusions we draw from them before returning to the broader question raised at the end.

First, it is correct that the extreme changes in behavioral consistency are indeed only observed in models trained with binary-choice training. As per the reviewer's suggestion,

we now marked these points from Fig 8F (former Fig 6F) and present this as an additional sub-panel in Fig S14A (former Fig S15, see below).

Second, it is also correct that Fig S14B shows that binary-choice training yielded behavioral patterns distinct from the other tested algorithms and the monkeys' behavior.

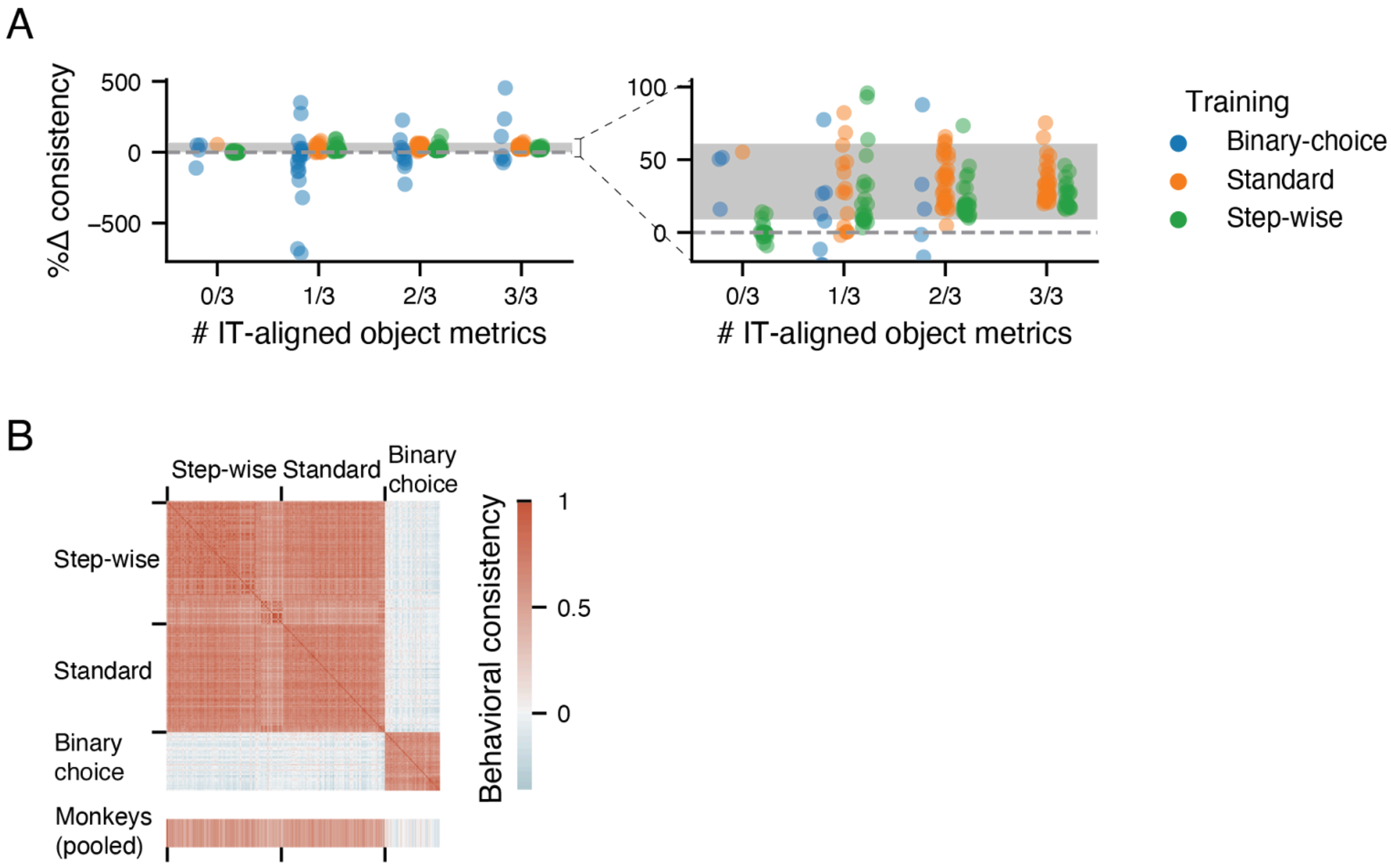

Figure S14. Binary-choice training produces distinct behavioral patterns from other training approaches and monkey behavior. (A) Binary-choice training predicts the most extreme differences in behavioral consistency. Replotted data from Fig 8D as a function of model training. All figure conventions follow those in Fig 8. (B) Comparison of image-specific behavioral patterns between trained models and monkeys. Noise-corrected correlations (i.e., consistency) between the image-level behavioral performance across 640 test images for all 208 models and the pooled monkey behavior. Individual models are ordered according to their training approach. While models trained with step-wise and standard training are highly consistent with each other after training, binary-choice-trained models were only consistent with themselves, but not with models from other training approaches. Image-wise performance patterns estimated from the behaviors of 3 trained monkeys are consistent with step-wise and standard training, but not with models trained with binary choice feedback. The bar height of the monkey data is increased to enhance visibility.

Third, Fig S12A *does not show IT-related decoding.* Instead, this plot shows the task performances of the finetuned models after task training. The overlay shows the monkey's behavioral performance, which we used as our first model screening criterion to ensure that any candidate IT-like model is on par with the monkey's performance. Fig S12A (pasted below for convenience) shows that 42 of the total 211 models passed this screening. For these models, we went on to test for a match with IT metrics. As a result of this screening process, the d'=0 models mentioned by the reviewer were never part of our evaluation and rejected from all consecutive analyses (including Fig 8 and Fig S14).

Fourth, Fig S12B shows how the models that passed the behavioral screening (Fig S12A) matched along the IT metrics. Here, we see that 7 of 42 models trained with binary-choice training do in fact mimic all three IT metrics (as shown as the dark blue

inset in the Training panel). While proportionally this is slightly worse than what we've seen for the other training approaches (standard: 39%, step-wise: 24%; binary-choice: 17%), in our eyes, this is not a complete disaster. There are, after all, a handful of models that matched all criteria.

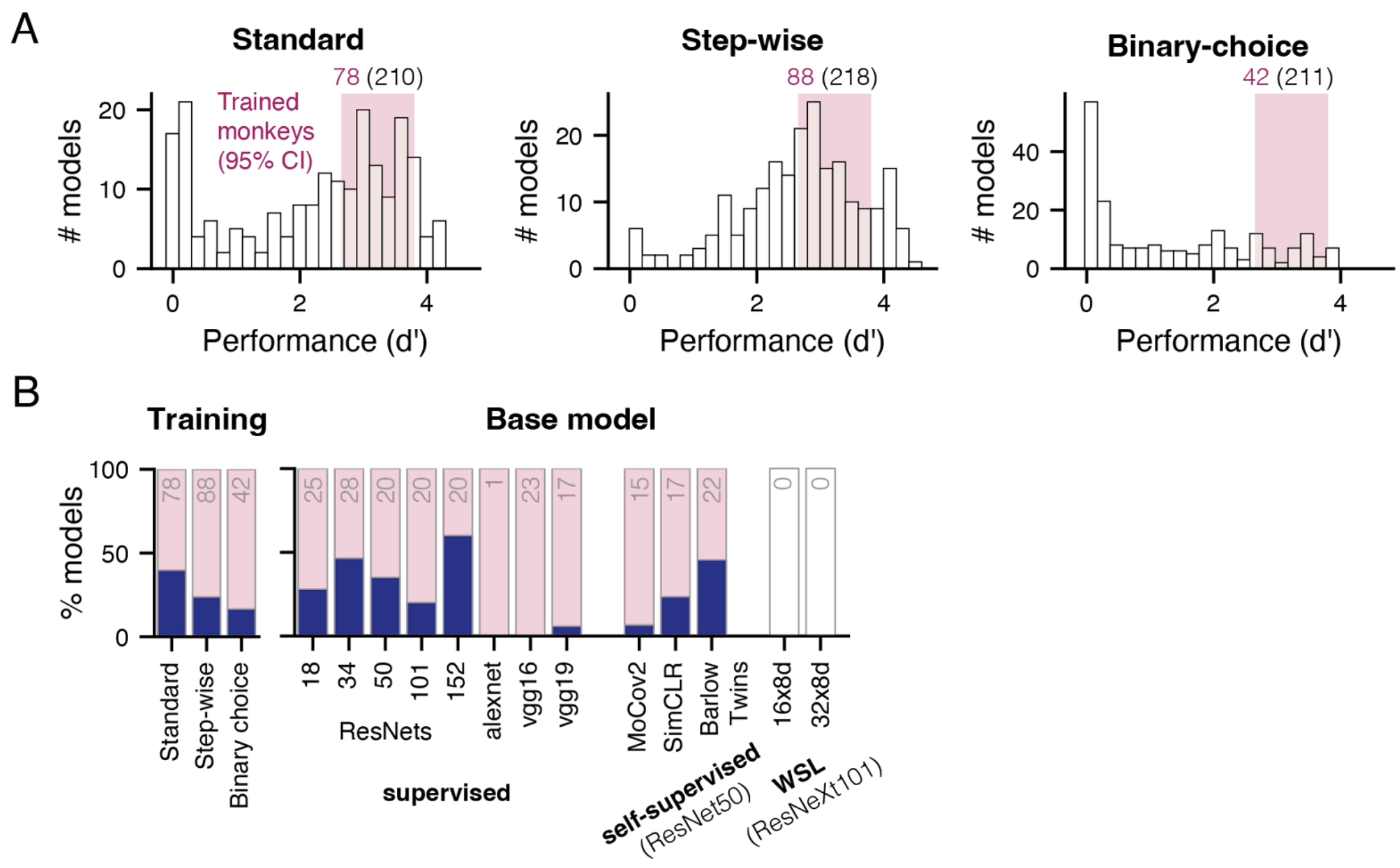

Figure S12. Model inclusion as a function of training parameters. (A) We only considered models as hypotheses of IT plasticity that matched the performance of the trained monkeys (magenta shaded areas depict the 95% CI of the pooled monkeys' behavior). Binary choice training produced the fewest equally performant models, followed by standard and stepwise training. Each training consisted of 20 model iterations (with different combinations of hyperparameters) for the 11 base models for which we could establish an IT-layer mapping. A few model trainings failed, thus producing fewer than 220 models. (B) Which training aspects were associated with producing IT-like models? Every training strategy and most base models could generate models that matched IT along all metrics. The annotated number refers to the number of models that passed behavioral screening or sufficient decoding to establish a mapping with IT responses. The filled blue bar only includes models that match all three metrics.

To summarize, based on the information presented up until Fig 8, we had no reason to believe that the IT-resembling models produced by binary-choice training would be qualitatively different from those produced by the other two training approaches. Returning to Fig 8, these sets of analyses served as a generalization test for the identified models. When predicting changes along identity-invariant properties, we did not observe that binary-choice-trained models differed from the rest. This can be seen in the figure below:

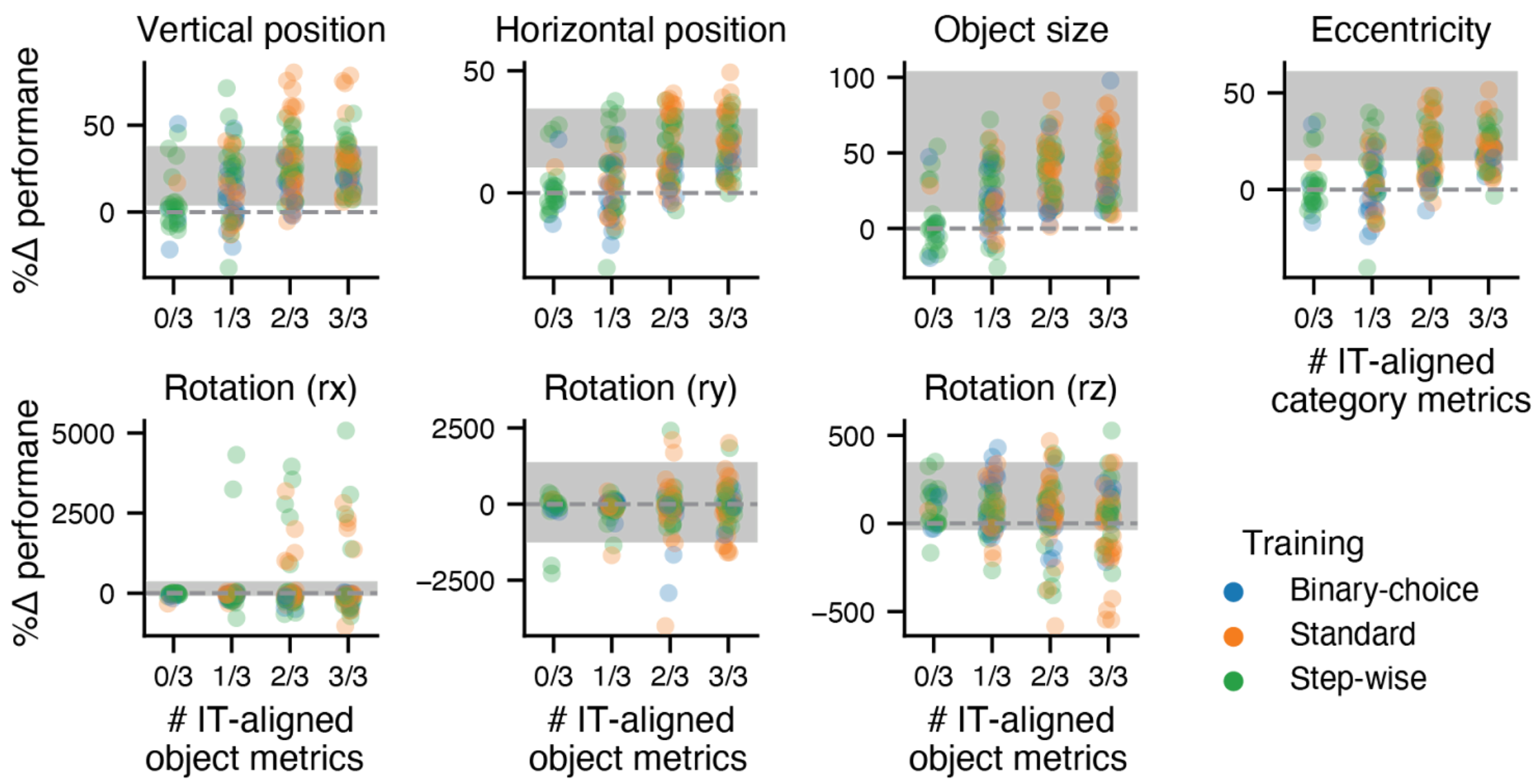

Figure R2. Most IT-like models best predict identity-invariant changes, irrespective of training type. Replotted data from Fig 8D as a function of model training. All figure conventions follow those in Fig 8.

In contrast, we noticed that binary-choice models stood out in their ability to predict changes in behavioral consistency. We visualized this in the newly added panel in Fig S14A. To investigate what drives these differences between training types, we performed behavioral consistency analyses comparing different models and monkey behavior (Fig. S14B). Here again, we observed that binary-choice training stood out from other training approaches and was also markedly dissimilar to monkey behavioral patterns. We think that this partly explains the results in Fig 8F.

Finally, the reviewer rightly asked why binary-choice training yields different behavioral patterns than the other training approaches. Our current interpretation is that these differences stem from how distractor information is harnessed during training. That is, in the binary-choice training, the information about the distractor classes was sparser compared to the other two approaches. We hypothesize that the difference in image-by-image patterns might arise because we only modeled the sensory aspects of the learning process, omitting, for example, reasoning about task structure. Such additional forms of learning enable inferences about how feedback can be interpreted: For example, if one has a good understanding of the task structure, one can know that on any given trial, there is a single correct target class and 7 possible distractor objects. Thus, even in the absence of a distractor class, the monkey can infer that choosing this (absent) class would have been incorrect. Our binary-choice feedback implementation did not include this structural inference step. Rather, we simply applied gradients to the target and one randomly selected distractor class per trial. It is easily conceivable that the trained monkeys (with extensive experience in this task) are capable of such abstraction and may have leveraged it during training.

The insight that processing error at the level of the task structure is essential for mimicking monkey image-specific behavior is an insight we gained while conducting this study. We believe it is a promising avenue for future work that deserves thorough theoretical investigation. Since such an in-depth investigation goes beyond the scope of this project, we now emphasize this direction in the discussion as an avenue for future work:

“Indeed, our observation that binary-choice training (learning about a single random

distractor class on a given trial) resulted in markedly different image-by-image performance patterns compared to other training approaches (which processed feedback about all distractor classes, Fig S14) already highlights the importance of inferences drawn from task structure for modeling primate behavior. Which computations are necessary for acquiring such task structure during the early phases of training is an exciting avenue for future work."

Discussion – Beyond IT: The Role of Downstream Networks in Object Learning

4. I believe the motivation in the introduction could flow a little better. Right now it identifies a nice "controversy" with some IT results suggesting plasticity and some suggesting none or very little. It would be helpful here to identify the particular choices of the experiment design here and why they might be relevant for reconciliation. For example, was relatively poorly physiological sampling a concern for some studies? Is the diversity of backgrounds used here vital? How about the number of categories? In short, a clearer delineation of previous limitations and how this study addresses those would be very helpful.

We thank the reviewer for this feedback. We now point out the advances in recording technology and our access to larger cohorts of macaques in the introduction:

"Prior studies addressing these hypotheses have relied on single or fewer electrodes in small samples of animals, limiting the ability to detect training-induced changes in IT that may be subtle, distributed across many neurons (Op de Beeck & Baker, 2010), and shared across individuals.

To address these limitations, we systematically tested these hypotheses using large-scale neural recordings from task-trained and naïve macaque IT cortex. Examining two cohorts of adult macaques (3 subjects per cohort), each implanted with multiple chronic recording arrays, we could not only re-examine prior findings across hundreds of recording sites but also quantify differences at the population level. Our results revealed modest but robust differences in IT representations linked to object discrimination training, supporting H1: IT recordings from trained animals demonstrated more object-selective responses at the level of individual recording sites and improved separability for the trained objects at the population level compared to training-naïve IT recordings."

Introduction

Reviewer #3 (Remarks to the Author):

The authors have responded to all of my concerns and even performed an additional control experiment, suggesting that the neural "training" effect is restricted to the trained stimuli (albeit based on a single-monkey comparison), which I appreciate. Upon rereading the manuscript, I have four remaining (minor) issues that should be addressed.

1. The authors now present data from individual animals (Figure S6) and conclude that the same trend is observed at the individual-subject level as in the pooled data. While this is the case for the d' metric, there is considerable variability across subjects for the other two metrics (the three data points of the naïve group are not consistently below those of the trained group). I therefore suggest softening the claim that these data "indicate robust training effects at the individual level."

We thank the reviewer for this careful observation. To reflect this more accurately, we have softened the corresponding statement in the Results section. The revised text now reads:

“Fig S6 demonstrates that the overall direction of effects is largely consistent with the group-level results shown in Fig 2, particularly for the decoding metric, though there is variability across subjects for the other metrics.”

Results – Task training enhances task-relevant information in IT

2. The inclusion of a table listing the recording sessions per monkey is informative. I do, however, have a question regarding the pooling of data across individual sessions: how confident are the authors that data from different sessions are statistically independent, given that recordings may have been obtained from the same units across multiple days using the chronic implants? Please comment on this issue.

We would like to clarify that our analyses do not assume statistical independence of units recorded on the same electrode across days. In fact, the rationale for pooling across sessions was precisely to aggregate responses from the same recording channels over time in order to obtain a more stable estimate of each channel’s response properties. Data from a given electrode were combined across days before entering the pseudo-population sampling procedure. Importantly, channels were never treated as independent observations across days. Instead, each channel contributed a single set of response estimates (after averaging across repetitions and sessions). Thus, potential resampling of the same neuronal population across days does not inflate the effective sample size. We want to clarify that the unit of resampling in our bootstrap procedure was the recording site, not the session.

3. Please provide the recording location(s) for the newly included Monkey P.

We have now added this information as an additional Supplemental Figure (Fig S8):

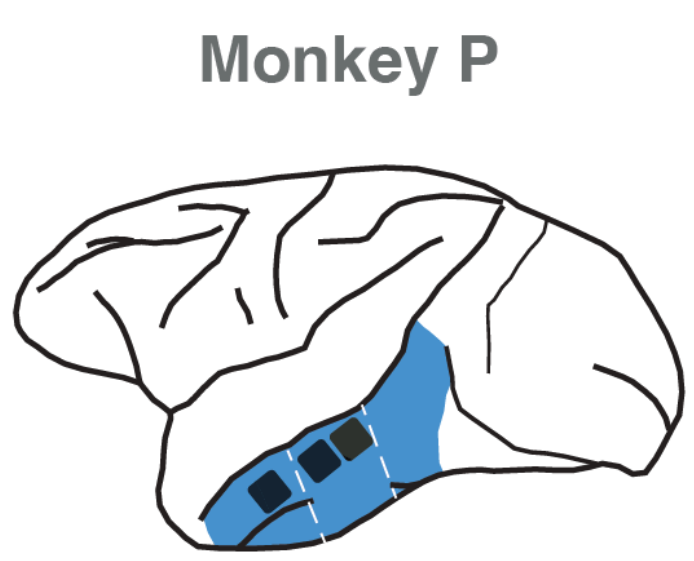

Figure S8. Micro-electrode array placements for Monkey P. Data from Monkey P were recorded for an additional control analysis presented in Fig 4.

***4. “*Monkeys completed hundreds of trials per session from the start of training, demonstrating familiarity with the task structure…” The authors should provide additional evidence that they were familiar with the task structure. That they completed hundreds of trials indicates that they were motivated to do/try something. I suppose they were trained before to perform the task with other stimuli. Please clarify.”**

We thank the reviewer for this important clarification. As suspected, the animals had previously been trained to perform the same match-to-sample task structure using different shapes and stimulus sets prior to the introduction of the training objects used in this study. Thus, at the onset of object training, the monkeys were already familiar with the task structure (trial initiation, sample presentation, two-alternative choice screen, reward contingencies), but were naïve to the specific object identities used here.

The fact that animals completed hundreds of trials per session from the start of training, together with their prior experience with the same task paradigm using other stimuli, supports our claim that they were familiar with the task structure and that the observed learning curves reflect object-specific learning rather than acquisition of the task rules themselves.

We have clarified this explicitly in the manuscript, which now reads:

"At the start of object training, subjects were already familiar with the match-to-sample task structure from prior training using different stimulus sets, but were naïve to the specific object identities used in the present study. Thus, while the animals had extensive experience with trial initiation, sample presentation, two-alternative choice screens, and reward contingencies, they had no prior exposure to the trained object categories (cf. Fig S2C). Subjects gradually improved their performance over the course of the training (Fig S2A, which likely reflected object-specific learning rather than acquisition of the task rules themselves), and they transitioned to the testing phase once their training performance saturated (for details on this criterion, see Rajalingham et al., 2015)."

Methods – Monkey training and behavioral tasks

Reviewer #4 (Remarks to the Author):

The authors have addressed my previous concerns by adding behavioral learning curves, additional analyses at the individual level, and clarifying the interpretation of the IT-behavior relationship. I have no remaining concerns and consider the manuscript suitable for publication.

Reviewer #5 (Remarks to the Author):

I co-reviewed this manuscript with one of the reviewers who provided the listed reports. This is part of the Nature Communications initiative to facilitate training in peer review and to provide appropriate recognition for Early Career Researchers who co-review manuscripts.
